# Supplementary material for: Community-based reconstruction and simulation of a full-scale model of the rat hippocampus CA1 region
Source: PLoS Biol. 2024 Nov 5;22(11):e3002861. doi: 10.1371/journal.pbio.3002861 (PMC11537418; doi:10.1371/journal.pbio.3002861)
Supplement: S1 Appendix — (PDF) [file pbio.3002861.s001.pdf]

S1 Appendix.

As an example of model improvement, we present here the steps to add a new cell type, i.e. a morpho-electrical type (me-type), to the network model (Figs S1 and S2). As explained in more details below, the process is composed of two major steps: the creating of single neuron model and the assembly of the network.

Creation of single neuron models

Requirements:

- 3D morphological reconstruction(s) of the morphological type (m-type).
- Electrophysiological recordings. They are commonly intracellular voltage recordings stimulated by a range of step current pulses to capture subthreshold and supratheshold spiking characteristics.
- (Optional) Additional datasets can be considered to validate the resulting models.

Processes:

1. Curate morphological reconstruction(s). This step includes, for example, the removal of zero-diameter branches, dangling branches, z-jumps, etc. (Section Morphology curation).
2. Clone and scale original morphologies to create a morphology library (Section Morphology library).
3. Curate the electrophysiological data. This step includes, for example, the correction for LJP, selection/exclusion of traces, etc.
4. Extract selected e-features from the electrophysiological recordings (Section Single neuron modeling).
5. Identify a set of ion channel models to be used (Section Ion channels).
6. Initialize a multicompartmental model combining one original (before cloning and scaling) morphology with the set of ion channel models.
7. Identify the electrical parameters that should be varied during the optimization and specify a range of values.
8. Run optimization to adjust ion channel parameterization to match the selected e-features.
9. Re-evaluate the previous steps may be required until a satisfactory model is obtained (Section Single neuron modeling).
10. (Optional) Further validate the accepted model(s) with additional datasets such as BPAP and PSP attenuation (Section Single neuron model validations).
11. The initial e-model(s) can be combined with the morphology library to produce a morpho-electrical

library with the same overall behavior of the initial e-model(s) (Section Library of neuron models).

Assembly of the network with the new cell type

Requirements:

- Neuronal density of new cell type in each layer.
- Morphological orientation of new cell type with respect to the three main axes of the hippocampus.
- List of all the modeled pathways from/to the cell type.
- Subcellular distribution of afferent synapses (soma, dendrites, AIS) for each pathway.
- (Optional) Bouton density for the cell type.
- (Optional) Number of synapses per connection for each pathway that includes the cell type.
- (Optional) Synaptic physiology parameters for each pathway that includes the cell type.
- (Optional) Additional datasets can be considered to validate the resulting network model.

Processes:

1. Add the new me-model(s) to the rest of me-model library.
2. Run the cell placement algorithm which includes the soma positioning to match the desired cell density layer profile (Section Soma placement), the cell orientation for each soma position (Section Cell orientation), and the selection of the best morphology for each soma position (Section Morphology selection).
3. Run the connectome algorithm to identify all potential synapses (Section Local synapse anatomy).
4. Estimate the pruning parameters. This step compares the connectome with all potential synapses with target values for bouton density and numbers of synapses per connection. If these values are not supplied for the new cell type, the algorithm uses values from other cell types.
5. Run the pruning algorithm which discards excess synapses based on the estimated pruning parameters (Section Local synapse anatomy).
6. Assign synaptic physiology parameters, which define the kinetics and short-term plasticity, to each of the remaining synapses. If these values are not supplied for the pathways that include the new cell type, generalization rules would apply parameters from the corresponding synaptic classes (Section Local synapse physiology).
7. (Optional) Additional datasets can be used to validate other aspects of the network model as, for example, divergence and convergence of different m-types, somatic PSP.
