# Supplementary material for: Community-based reconstruction and simulation of a full-scale model of the rat hippocampus CA1 region
Source: PLoS Biol. 2024 Nov 5;22(11):e3002861. doi: 10.1371/journal.pbio.3002861 (PMC11537418; doi:10.1371/journal.pbio.3002861)
Supplement: S1 Fig — Black rectangles represent the different building blocks, and the blue labels are the processes between blocks. (PDF) [file pbio.3002861.s002.pdf]

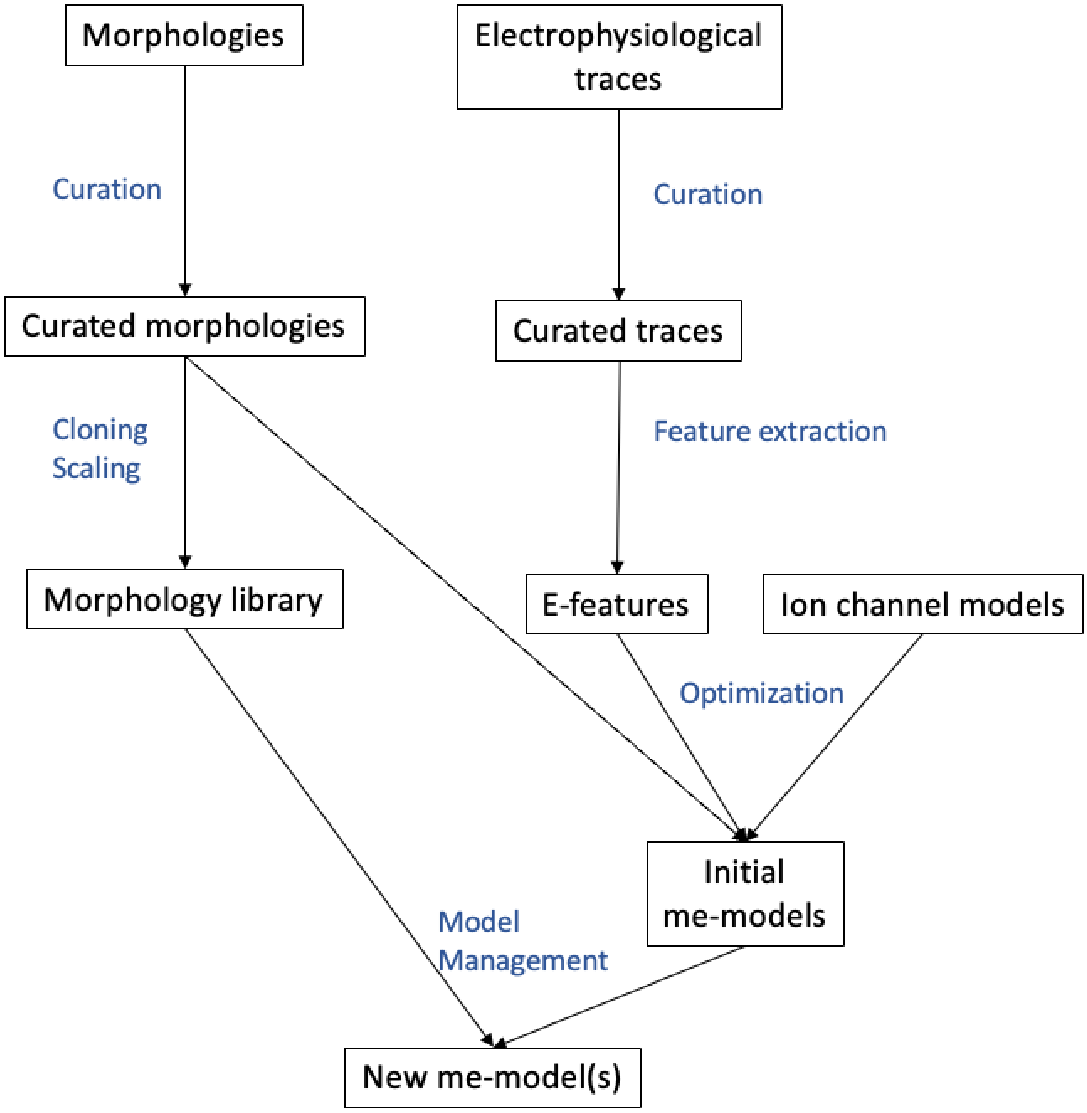

Figure S1: **Workflow for creating new single cell models**. Black rectangles represent the different building blocks, and the blue labels are the processes between blocks.
