# Supplementary material for: Community-based reconstruction and simulation of a full-scale model of the rat hippocampus CA1 region
Source: PLoS Biol. 2024 Nov 5;22(11):e3002861. doi: 10.1371/journal.pbio.3002861 (PMC11537418; doi:10.1371/journal.pbio.3002861)
Supplement: S2 Fig — Black rectangles represent the different building blocks, the green boxes are the configuration files, and the blue labels are the processes between blocks. (PDF) [file pbio.3002861.s003.pdf]

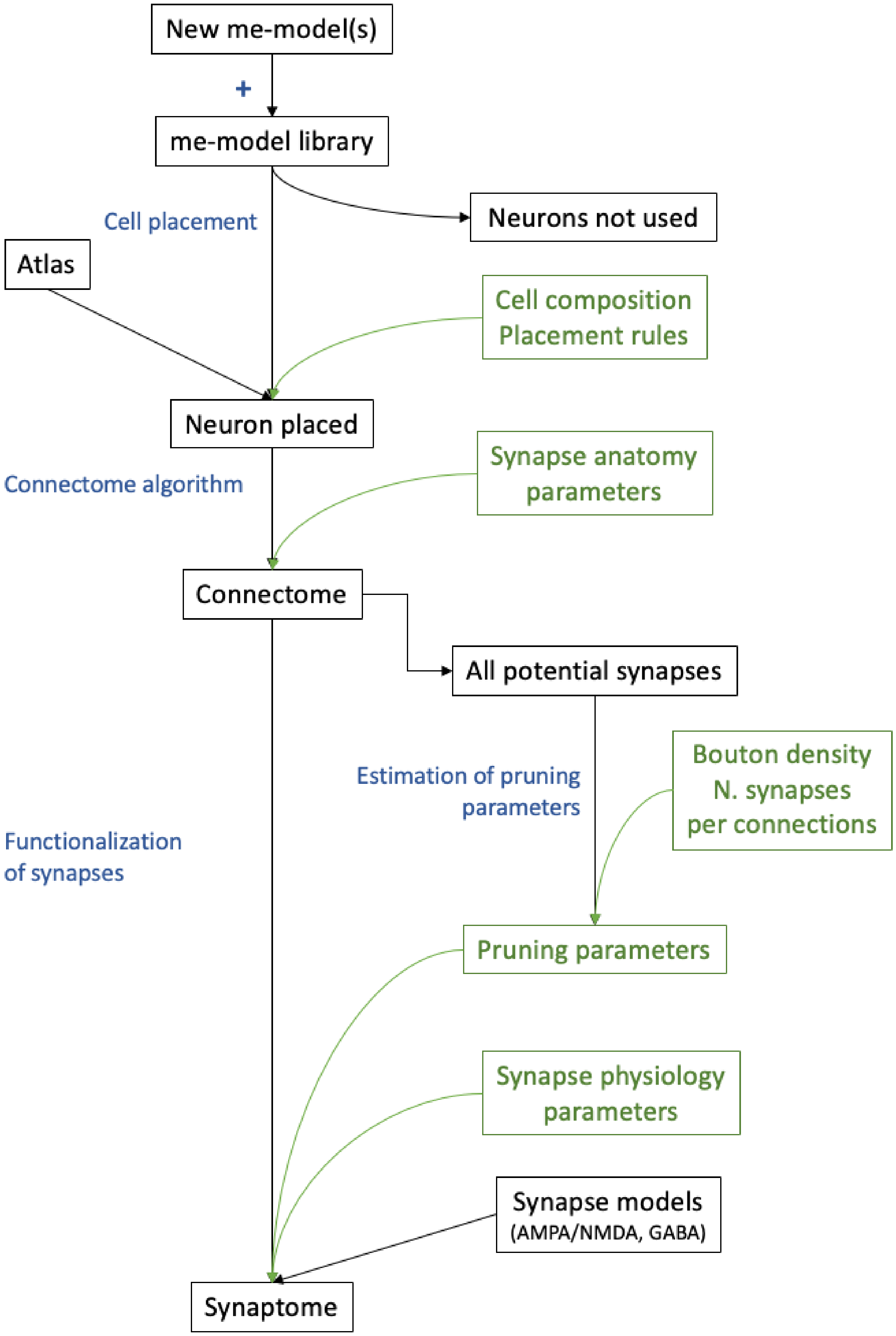

Figure S2: **Workflow for adding new single cell models to the network model**. Black rectangles represent the different building blocks, the green boxes are the configuration files, and the blue labels are the processes between blocks.
