# Supplementary material for: Community-based reconstruction and simulation of a full-scale model of the rat hippocampus CA1 region
Source: PLoS Biol. 2024 Nov 5;22(11):e3002861. doi: 10.1371/journal.pbio.3002861 (PMC11537418; doi:10.1371/journal.pbio.3002861)
Supplement: S3 Fig — Counts per year of the number of publications based on a Pubmed search for “hippocampus—cornu Ammonis—CA1—CA2—CA3” from 1900-present indicates the large size of the neuroscientific community researching hippocampus. (PDF) [file pbio.3002861.s004.pdf]

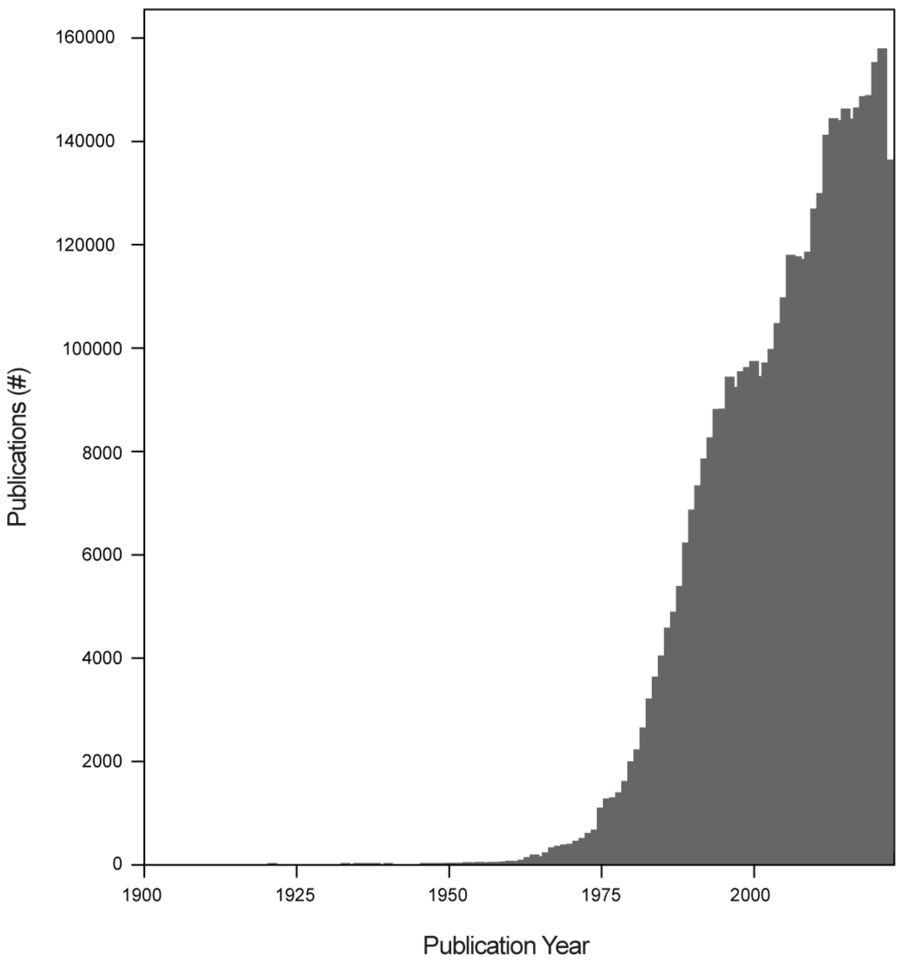

Figure S3: **Scientific publications on hippocampus progressively increase**. Counts per year of the number of publications based on a pubmed search for 'hippocampus|cornu Ammonis|CA1|CA2|CA3' from 1900-present indicates the large size of neuroscientific community researching hippocampus.
