# Supplementary material for: Community-based reconstruction and simulation of a full-scale model of the rat hippocampus CA1 region
Source: PLoS Biol. 2024 Nov 5;22(11):e3002861. doi: 10.1371/journal.pbio.3002861 (PMC11537418; doi:10.1371/journal.pbio.3002861)
Supplement: S4 Fig — Simplified workflow of the circuit building. Boxes represent the different building blocks, while blue labels are the operations between blocks. (PDF) [file pbio.3002861.s005.pdf]

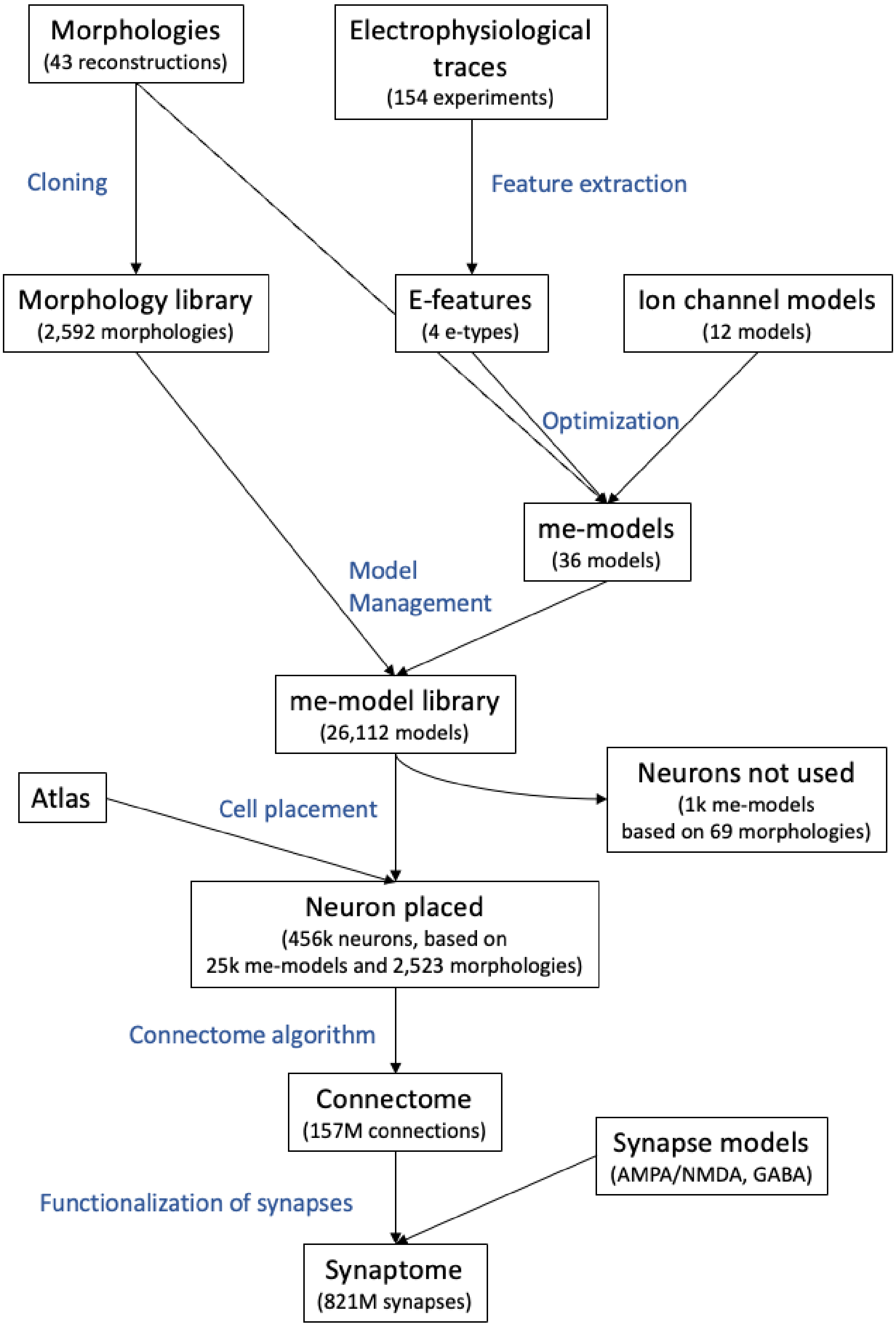

Figure S4: **Circuit building workflow**. Simplified workflow of the circuit building. Boxes represent the different building blocks, while blue labels are the operations between blocks.
