# Supplementary material for: Community-based reconstruction and simulation of a full-scale model of the rat hippocampus CA1 region
Source: PLoS Biol. 2024 Nov 5;22(11):e3002861. doi: 10.1371/journal.pbio.3002861 (PMC11537418; doi:10.1371/journal.pbio.3002861)
Supplement: S5 Fig — Validations of cloning methods with a similarity metric (differences between median values divided by variance). (A) The mean scores for 21 unique morphometrics averaged across m-types shown for basal and apical dendrites, and axons in each row. (B) The same scoring grouped by m-types instead of metrics for basal and apical dendrites. Since inhibitory neurons did not have apical dendrites, we show its score only for pyramidal cells. (C) Similar to B, but calculated for axons. Excitatory and inhibitory axons are grouped separately. (PDF) [file pbio.3002861.s006.pdf]

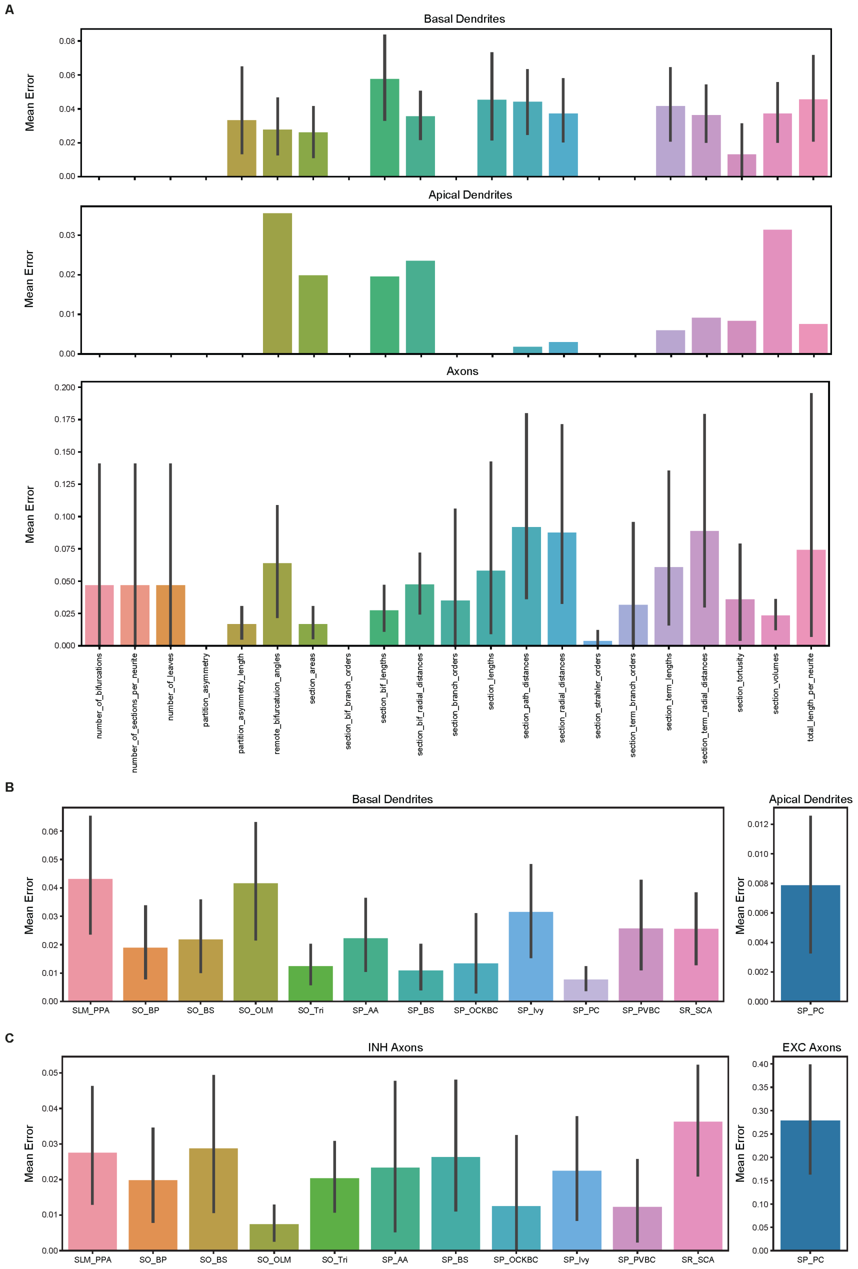


Figure S5: **Validation of cloning**. Validations of cloning methods with a similarity metric (differences between median values divided by variance). A. The mean scores for 21 unique morphometrics averaged across m-types shown for basal and apical dendrites, and axons in each row. B. The same scoring grouped by m-types instead of metrics for basal and apical dendrites. Since inhibitory neurons did not have apical dendrites, we show its score only for pyramidal cells. C. Similar to B, but calculated for axons. Excitatory and inhibitory axons are grouped separately.
