# Supplementary material for: Community-based reconstruction and simulation of a full-scale model of the rat hippocampus CA1 region
Source: PLoS Biol. 2024 Nov 5;22(11):e3002861. doi: 10.1371/journal.pbio.3002861 (PMC11537418; doi:10.1371/journal.pbio.3002861)
Supplement: S6 Fig — (A) Persistence diagrams of original (blue), repaired (green), and cloned (red) dendrites. Persistence diagrams encode the start (y-axis) and end (x-axis) radial distances of all dendritic trees in the respective populations of neurons. (B) Persistence diagrams of normalized radial distances (to one) from original (blue), repaired (green), and cloned (red) dendrites. (C) Persistence images are the Gaussian averages of the respective persistence diagrams (B) from original (top), repaired (middle) and cloned (bottom) dendrites. Calculations are computed for the all cell groups. For more detailed analyses, see S7 and S8 Figs. (PDF) [file pbio.3002861.s007.pdf]

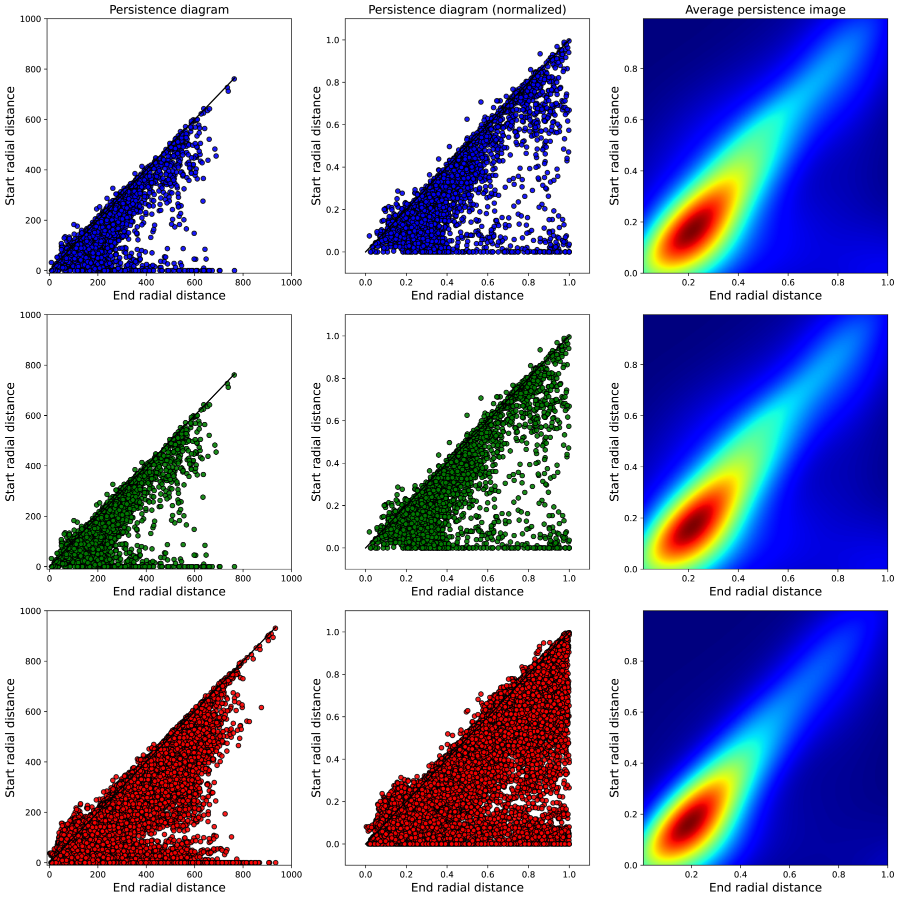

Figure S6: **Increasing morphological diversity**. A. Persistence diagrams of original (blue), repaired (green) and cloned (red) dendrites. Persistence diagrams encode the start (y-axis) and end (x-axis) radial distances of all dendritic trees in the respective populations of neurons. B. Persistence diagrams of normalized radial distances (to one) from original (blue), repaired (green) and cloned (red) dendrites. C. Persistence images are the Gaussian averages of the respective persistence diagrams (B) from original (top), repaired (middle) and cloned (bottom) dendrites. Calculations are computed for the all cell groups. For more detailed analyses, see Figs S7 and S8.
