# Supplementary material for: Community-based reconstruction and simulation of a full-scale model of the rat hippocampus CA1 region
Source: PLoS Biol. 2024 Nov 5;22(11):e3002861. doi: 10.1371/journal.pbio.3002861 (PMC11537418; doi:10.1371/journal.pbio.3002861)
Supplement: S7 Fig — (A) Persistent images averaged across each m-type for original morphologies and for their cloned counterparts with respect to the basal dendrites, and (B) their differences. (C) The same process applied for apical dendrites which was only present in the excitatory cell group (e.g., pyramidal cells). Note that the radial distance is synonymous with Euclidean distance and should not be mixed with hippocampal radial axis. (PDF) [file pbio.3002861.s008.pdf]

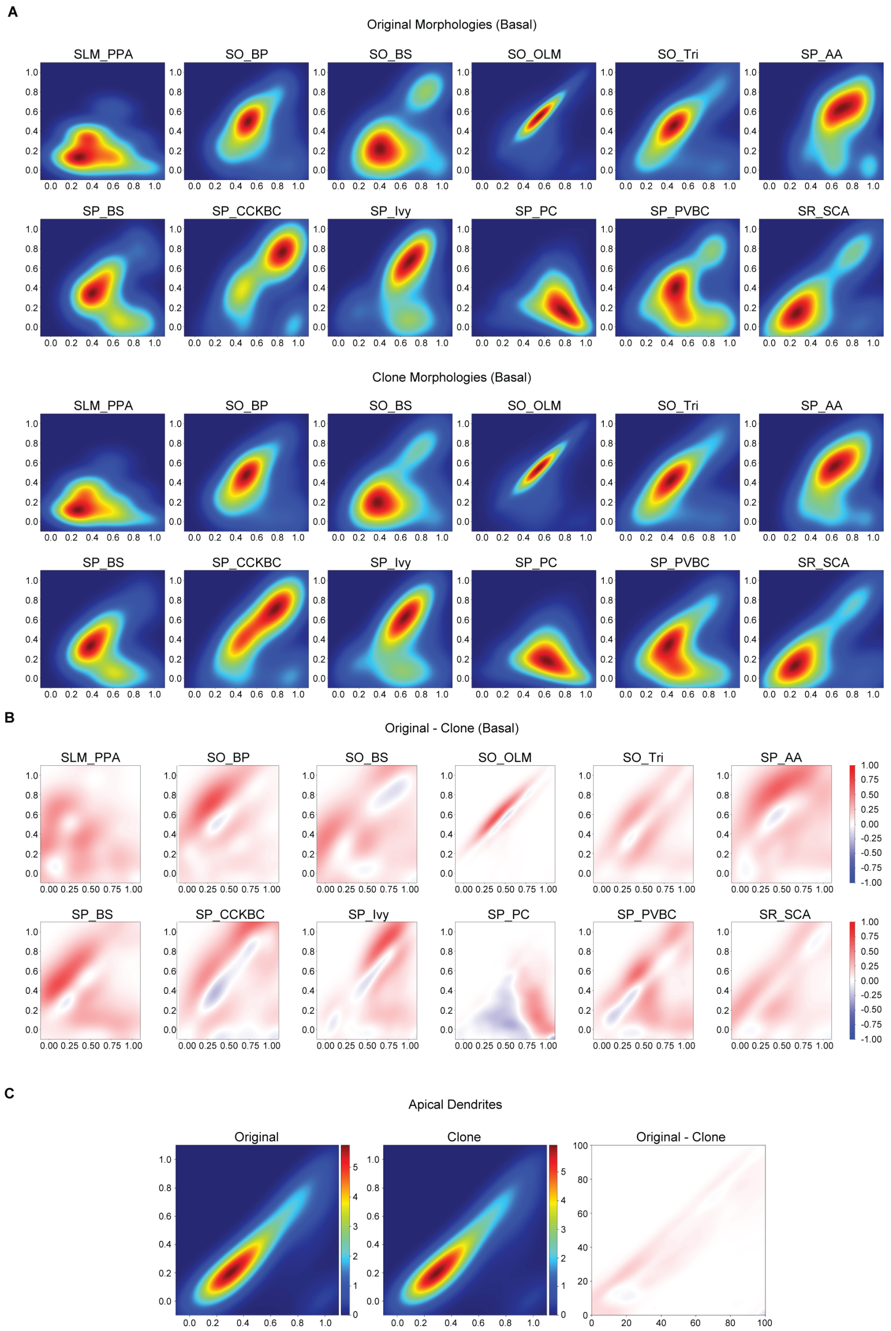


Figure S7: **Persistent images for the basal and apical dendrites of each m-type**. A. Persistent images averaged across each m-type for original morphologies and for their cloned counterparts with respect to the basal dendrites, and (B) their differences. C. The same process applied for apical dendrites which was only present in the excitatory cell group (e.g., pyramidal cells). Note that the radial distance is synonymous with Euclidean distance, and should not be mixed with hippocampal radial axis.
