# Supplementary material for: Community-based reconstruction and simulation of a full-scale model of the rat hippocampus CA1 region
Source: PLoS Biol. 2024 Nov 5;22(11):e3002861. doi: 10.1371/journal.pbio.3002861 (PMC11537418; doi:10.1371/journal.pbio.3002861)
Supplement: S8 Fig — Persistent images averaged across each cell type for (A) original axons, (B) cloned versions, and (C) their difference. (PDF) [file pbio.3002861.s009.pdf]

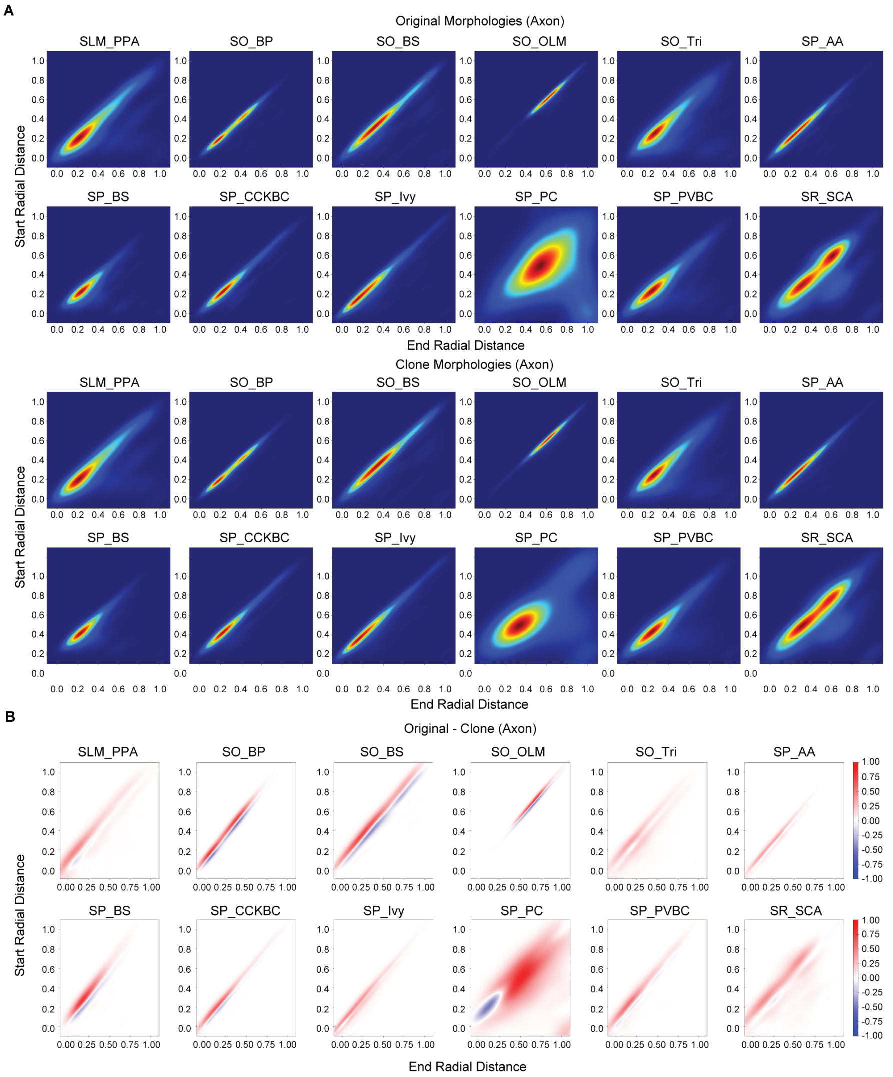

Figure S8: **Persistent images for the axons of each m-type**. Persistent images averaged across each cell type for A. original axons, B. cloned versions, and C. their difference.
