# Supplementary material for: Community-based reconstruction and simulation of a full-scale model of the rat hippocampus CA1 region
Source: PLoS Biol. 2024 Nov 5;22(11):e3002861. doi: 10.1371/journal.pbio.3002861 (PMC11537418; doi:10.1371/journal.pbio.3002861)
Supplement: S9 Fig — (A) Graphical illustration of the back-propagating action potential protocol. In a pyramidal cell model, the soma is stimulated to elicit an AP, which is then measured at different distances from the soma. (B) In silico measurements (black dots) are reported and compared with experimental data from Golding and colleagues (red dots and whiskers, indicating mean and standard deviation). (C) Graphical illustration of the postsynaptic potential attenuation protocol. Dendrites on the apical trunk of a pyramidal cell are stimulated with bi-exponential currents; then, PSP’s amplitudes are measured as it travels toward the soma. (D) In silico measurements of PSP attenuation (black dots) are expressed as a ratio between the PSP measured in the dendrite and the one measured in the soma. Pyramidal cell models are compared with experimental data from Magee and Cook. The 2 distributions have been fitted with exponential equations, resulting in the following space constants: τ model = 155.6 μm and τ experiment = 235.2 μm. (PDF) [file pbio.3002861.s010.pdf]

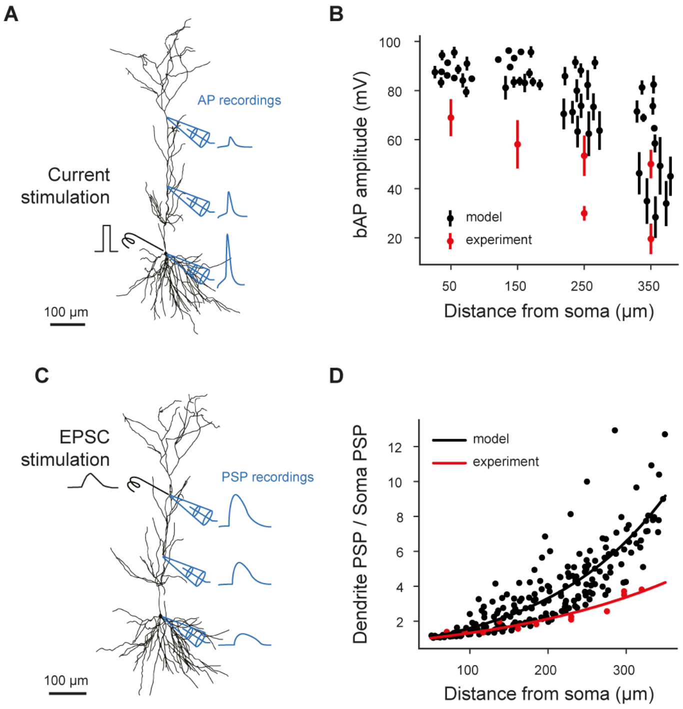

Figure S9: **Validation of single-cell models**. A. Graphical illustration of the back-propagating action potential protocol. In a pyramidal cell model, the soma is stimulated to elicit an AP, which is then measured at different distances from the soma. B. *In silico* measurements (black dots) are reported and compared with experimental data from Golding et al., 2001 (doi:10.1152/jn.2001.86.6.2998) (red dots and whiskers, indicating mean and standard deviation). C. Graphical illustration of the post-synaptic potential attenuation protocol. Dendrites on the apical trunk of a pyramidal cell are stimulated with bi-exponential currents; then, PSP's amplitudes are measured as it travels toward the soma. D. *In silico* measurements of PSP attenuation (black dots) are expressed as a ratio between the PSP measured in the dendrite and the one measured in the soma. Pyramidal cell models are compared with experimental data from Magee and Cook, 2000 (doi:10.1038/78800). The two distributions have been fitted with exponential equations, resulting in the following space constants: τmodel $= 155.6\,\mu\text{m}$ and τexperiment $= 235.2\,\mu\text{m}$.
