# Supplementary material for: Community-based reconstruction and simulation of a full-scale model of the rat hippocampus CA1 region
Source: PLoS Biol. 2024 Nov 5;22(11):e3002861. doi: 10.1371/journal.pbio.3002861 (PMC11537418; doi:10.1371/journal.pbio.3002861)
Supplement: S10 Fig — (A) Original atlas. (B) Smoothed atlas. (C) Upper and lower shells. (D) Centerline through the volume. (E) Planes normal to the centerline. (F) Longitudinal, transverse, and radial coordinates. (G) Orientation vectors. (H) Layer assignment. (PDF) [file pbio.3002861.s011.pdf]

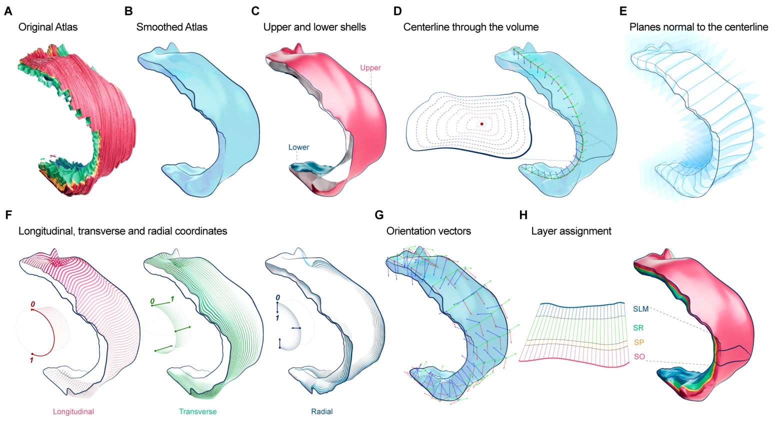

Figure S10: **CA1 atlas overview**. A. Original atlas. B. Smoothed atlas. C. Upper and lower shells. D. Centerline through the volume. E. Planes normal to the centerline. F. Longitudinal, transverse and radial coordinates. G. Orientation vectors. H. Layer assignment.
