# Supplementary material for: Community-based reconstruction and simulation of a full-scale model of the rat hippocampus CA1 region
Source: PLoS Biol. 2024 Nov 5;22(11):e3002861. doi: 10.1371/journal.pbio.3002861 (PMC11537418; doi:10.1371/journal.pbio.3002861)
Supplement: S11 Fig — A pyramidal cell (PC) is used to illustrate the cell placement in CA1 volume. (A) We set a density profile for each cell type. In the case of PC, we set an uniform density in stratum pyramidal (SP). (B) We randomly identify soma positions matching the given cell density. (C) For each soma position, we assign a morphology and orient it using the vector fields. For PC, we align the main axis of the dendrites with a vector (red arrow) parallel to the radial axis, while we align the axon to a vector (green arrow) parallel to the transverse axis. Furthermore, the more complex branch of the axon points to the Subiculum. (D) We select a morphology that respects a set of rules. In the case of PC, we specify 2 rules regarding the dendrites. (PDF) [file pbio.3002861.s012.pdf]

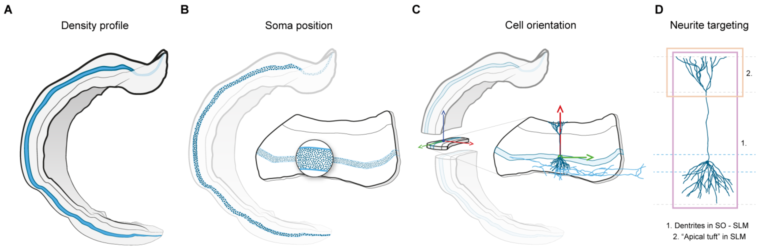

Figure S11: **Cell placement**. A pyramidal cell (PC) is used to illustrate the cell placement in CA1 volume. A. We set a density profile for each cell type. In the case of PC, we set an uniform density in stratum pyramidal (SP). B. We randomly identify soma positions matching the given cell density. C. For each soma position, we assign a morphology and orient it using the vector fields. For PC, we align the main axis of the dendrites with a vector (red arrow) parallel to the radial axis, while we align the axon to a vector (green arrow) parallel to the transverse axis. Furthermore, the more complex branch of the axon points to the Subiculum. D. We select a morphology that respects a set of rules. In the case of PC, we specify two rules regarding the dendrites.
