# Supplementary material for: Community-based reconstruction and simulation of a full-scale model of the rat hippocampus CA1 region
Source: PLoS Biol. 2024 Nov 5;22(11):e3002861. doi: 10.1371/journal.pbio.3002861 (PMC11537418; doi:10.1371/journal.pbio.3002861)
Supplement: S12 Fig — (A) Validation of the cell placement. A subset of cells from each m-type is displayed within each of the 100 slices of 100 μm thickness equally spanned along the longitudinal axis. (B) Cell composition is sampled in different subvolumes (9 cylinders of 300 μm of radius equally spanned along the longitudinal axis) and compared with desired composition from Bezaire and Soltesz (R = 0.999998, p < 0.0001). (C) Total cell density in CA1, in the layers SLM + SR, SP, pyramidal cell density in SP, total cell density in SO. Neuron density validation is intrinsic (PC in SP) and extrinsic (the rest). The density is sampled in different subvolumes (9 cylinders of 300 μm of radius equally spanned along the longitudinal axis). Experimental values can be found in S5 Table. (PDF) [file pbio.3002861.s013.pdf]

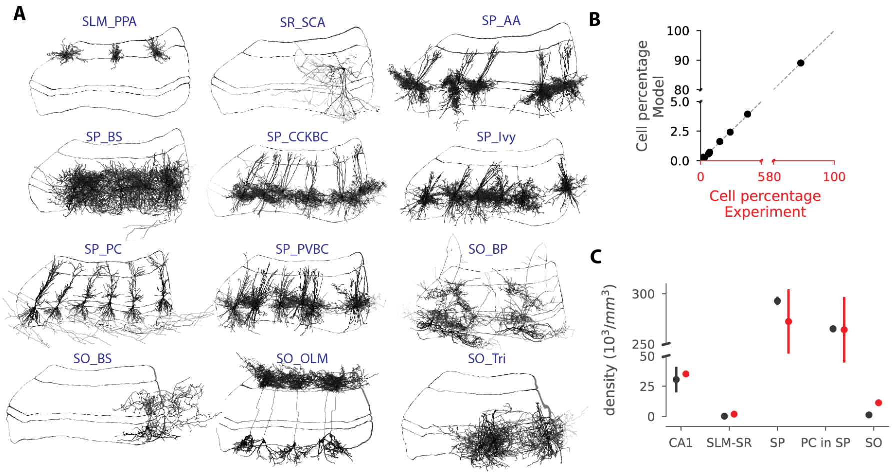

Figure S12: **Validation of cell placement**. A. Validation of the cell placement. A subset of cells from each m-type is displayed within each of the 100 slices of $100\,\mu\mathrm{m}$ thickness equally spanned along the longitudinal axis. B. Cell composition is sampled in different subvolumes (9 cylinders of $300\,\mu\mathrm{m}$ of radius equally spanned along the longitudinal axis) and compared with desired composition from Bezaire and Soltesz, 2013 (doi:10.1002/hipo.22141) ($R = 0.999998$, $p < 0.0001$). C. Total cell density in CA1, in the layers SLM + SR, SP, pyramidal cell density in SP, total cell density in SO. Neuron density validation is intrinsic (PC in SP) and extrinsic (the rest). The density is sampled in different subvolumes (9 cylinders of $300\,\mu\mathrm{m}$ of radius equally spanned along the longitudinal axis). Experimental values can be found in Table S5.
