# Supplementary material for: Community-based reconstruction and simulation of a full-scale model of the rat hippocampus CA1 region
Source: PLoS Biol. 2024 Nov 5;22(11):e3002861. doi: 10.1371/journal.pbio.3002861 (PMC11537418; doi:10.1371/journal.pbio.3002861)
Supplement: S13 Fig — We compare the available data on the number of synapses per connection of a given pathway to the corresponding number of appositions per connection. Data can be grouped in 2 sets and fit separately (purple line y = 0.1096x for I-I, red line y = 1.1690x for the rest). The fitting lines can be used to predict how much the appositions should be pruned to match or predict synapses per connection. Experimental values can be found in S9 Table. E: excitatory neuron, I: inhibitory neuron. (PDF) [file pbio.3002861.s014.pdf]

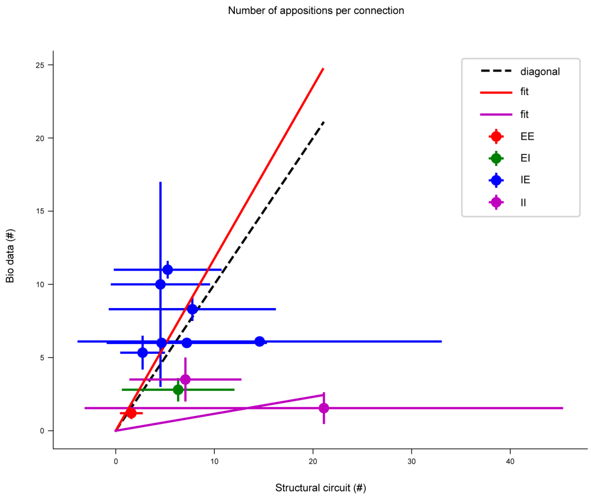


Figure S13: **Prediction of the number of synapses per connection from the number of appositions per connections**. We compare the available data on the number of synapses per connection of a given pathway to the corresponding number of appositions per connection. Data can be grouped in two sets and fit separately (purple line $y = 0.1096x$ for I-I, red line $y = 1.1690x$ for the rest). The fitting lines can be use to predict how much the appositions should be pruned to match or predict synapses per connections. Experimental values can be found in table S9. E: excitatory neuron, I: inhibitory neuron.
