# Supplementary material for: Community-based reconstruction and simulation of a full-scale model of the rat hippocampus CA1 region
Source: PLoS Biol. 2024 Nov 5;22(11):e3002861. doi: 10.1371/journal.pbio.3002861 (PMC11537418; doi:10.1371/journal.pbio.3002861)
Supplement: S14 Fig — (A) Mean and STD of bouton density values per individual m-type. (B) Mean synapses per connection for each m-type pair. Experimental values are given in square brackets. (C) Comparisons of mean and STD of synapses per connection for experimental data points and corresponding values in the model. Experimental values in panels A and C can be found respectively in S7 and S9 Tables. (PDF) [file pbio.3002861.s015.pdf]

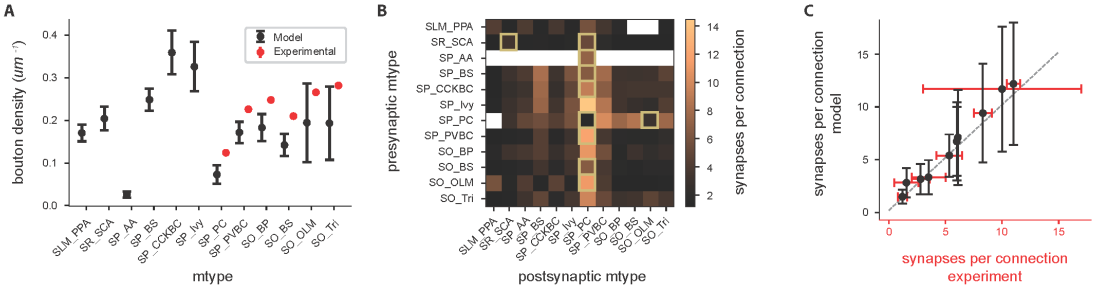

Figure S14: **Bouton density and synapses per connection.** A. Mean and std of bouton density values per individual m-type. B. Mean synapses per connection for each m-type pair. Experimental values are given in square brackets. C. Comparisons of mean and std of synapses per connection for experimental data points and corresponding values in the model. Experimental values in panels A and C can be found respectively in tables S7 and S9.
