# Supplementary material for: Community-based reconstruction and simulation of a full-scale model of the rat hippocampus CA1 region
Source: PLoS Biol. 2024 Nov 5;22(11):e3002861. doi: 10.1371/journal.pbio.3002861 (PMC11537418; doi:10.1371/journal.pbio.3002861)
Supplement: S15 Fig — (A) Connection probability with respect to the intersomatic distance within and between Excitatory (E) and Inhibitory (I) groups. (B) Mean connection probability for each type pair. Non-existing connections are left blank and experimental observations are shown with square brackets. (C) Comparison of experimental and model connection probabilities within E/I group pairs. Only pairs of neurons with a maximum inter-somatic distance of 500 μm were considered. Experimental values can be found in S8 Table. (PDF) [file pbio.3002861.s016.pdf]

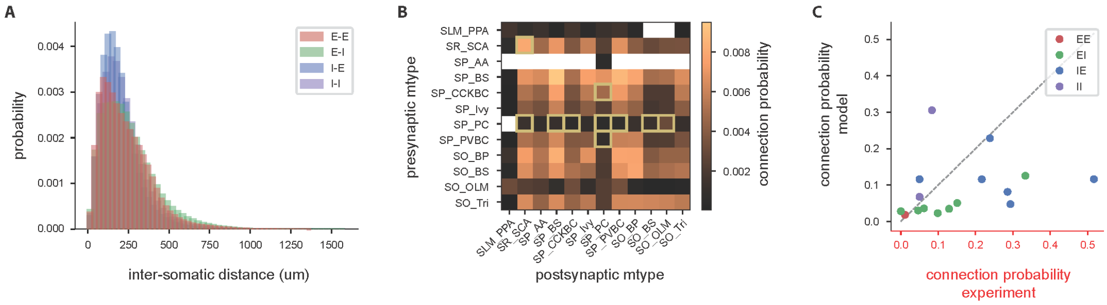

Figure S15: **Connection probability** A. Connection probability with respect to the intersomatic distance within and between Excitatory (E) and Inhibitory (I) groups. B. Mean connection probability for each m-type pair. Non-existing connections are left blank and experimental observations are shown with square brackets. C. Comparison of experimental and model connection probabilities within E/I group pairs. Only pairs of neurons with a maximum inter-somatic distance of $500\,\mu\mathrm{m}$ were considered. Experimental values can be found in Table S8.
