# Supplementary material for: Community-based reconstruction and simulation of a full-scale model of the rat hippocampus CA1 region
Source: PLoS Biol. 2024 Nov 5;22(11):e3002861. doi: 10.1371/journal.pbio.3002861 (PMC11537418; doi:10.1371/journal.pbio.3002861)
Supplement: S16 Fig — (A) Indegree distribution for neurons in this model. The inset shows the distribution on logarithmic scale. (B) Number of synapses made on an average postsynaptic cell from each afferent m-type group. Colorbar in log-normal scale. (C) Mean and STD of synapses made onto an average neuron for each m-type. (D) Number of synapses made onto each neurite type for excitatory and inhibitory classes (Megias and colleagues). (PDF) [file pbio.3002861.s017.pdf]

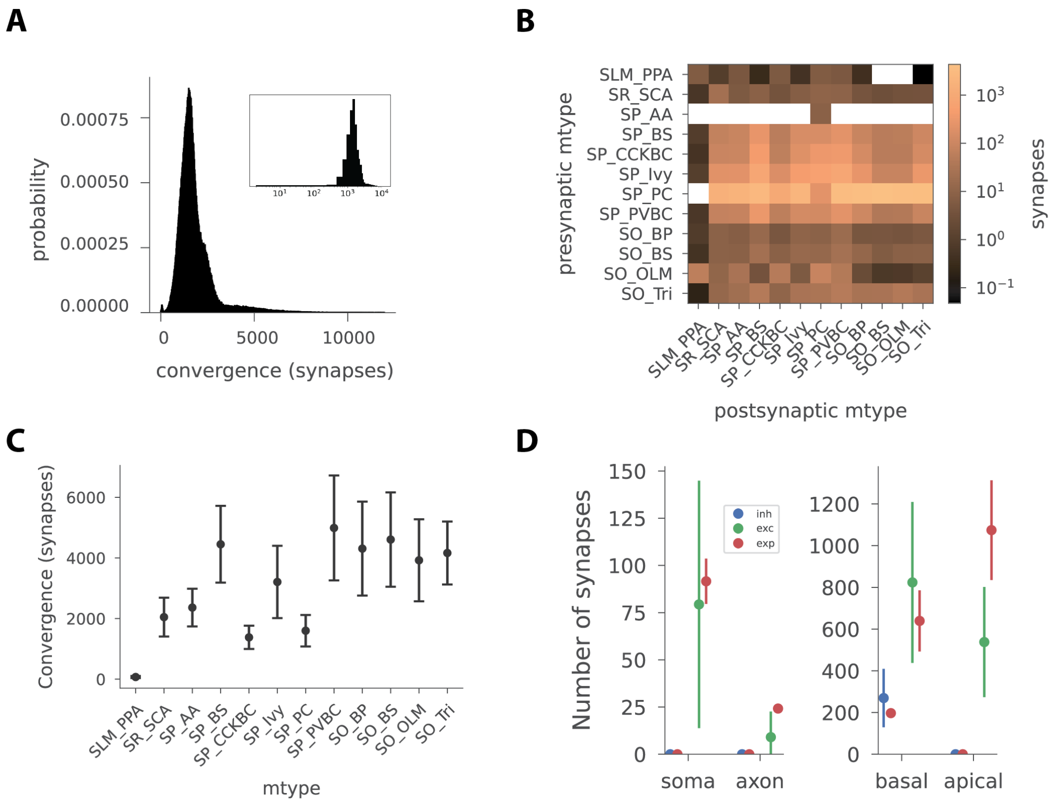

Figure S16: **Convergence on neurons and neuron groups.** A. Indegree distribution for neurons in this model. The inset shows the distribution on logarithmic scale. B. Number of synapses made on an average post-synaptic cell from each afferent m-type group. Colorbar in log-normal scale. C. Mean and std of synapses made onto an average neuron for each m-type. D. Number of synapses made onto each neurite type for excitatory and inhibitory classes (Megias et al., 2001. doi:10.1016/S0306-4522(00)00496- 6
