# Supplementary material for: Community-based reconstruction and simulation of a full-scale model of the rat hippocampus CA1 region
Source: PLoS Biol. 2024 Nov 5;22(11):e3002861. doi: 10.1371/journal.pbio.3002861 (PMC11537418; doi:10.1371/journal.pbio.3002861)
Supplement: S17 Fig — (A) Outdegree distribution for neurons in this model. The inset shows the distribution on logarithmic scale. (B) Number of synapses made by an average neuron from presynaptic m-type to postsynaptic group. (C) Comparison of divergence per m-type with the available experimental values. (D) Prediction of synapse divergence broken down into efferent synaptic group. SO_Tri divergence differs significantly from the experimental data. This could be explained by a morphological reconstruction which is not representative of the entire class or other factors that favor the connections between SO_Tri and interneurons beyond chance. (E) Percentage of synapses made onto individual layers or outside the CA1 mesh. For SR_SCA, the discrepancy between model and experiment can be explain by different morphological subtypes used in the 2 cases. In the experiment of Pawelzik and colleagues, the morphology is well confined in SR, while in the morphology used in the model also invades SP and SO. For D and E, hatched bars indicate the experimental values. Experimental values in panels C–E can be found respectively in S11 and S12 Tables. (PDF) [file pbio.3002861.s018.pdf]

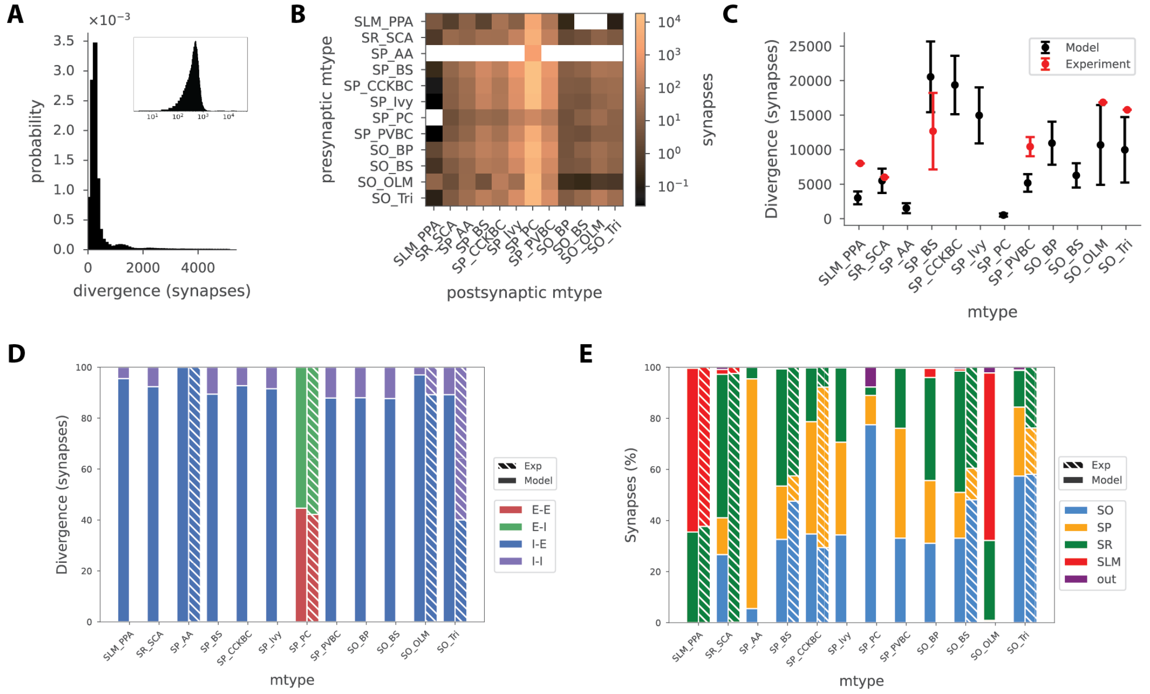

Figure S17: **Divergence of neurons and neuron groups.** A. Outdegree distribution for neurons in this model. The inset shows the distribution on logarithmic scale. B. Number of synapses made by an average neuron from presynaptic m-type to postsynaptic group. C. Comparison of divergence per m-type with the available experimental values. D. Prediction of synapse divergence broken down into efferent synaptic group. SO_Tri divergence differs significantly from the experimental data. This could be explained by a morphological reconstruction which is not representative of the entire class or other factors that favor the connections between SO_Tri and interneurons beyond chance. E. Percentage of synapses made onto individual layers or outside the CA1 mesh. For SR_SCA, the discrepancy between model and experiment can be explain by different morphological sub-types used in the two cases. In the experiment of Pawelzik et al., 2002 (doi:10.1002/cne.10118), the morphology is well confined in SR, while in the morphology used in the model also invades SP and SO. For D and E, hatched bars indicate the experimental values. Experimental values in panels C-E can be found respectively in tables S11-S12.
