# Supplementary material for: Community-based reconstruction and simulation of a full-scale model of the rat hippocampus CA1 region
Source: PLoS Biol. 2024 Nov 5;22(11):e3002861. doi: 10.1371/journal.pbio.3002861 (PMC11537418; doi:10.1371/journal.pbio.3002861)
Supplement: S18 Fig — (A) Example of in silico paired recording experiment between a PC (purple morphology) and a PVBC (light blue morphology), synapses between PC axon and PVBC dendrites in SO are depicted as yellow spheres. The presynaptic neuron is stimulated with a step current to elicit an action potential. Somatic recordings of EPSP and EPSC are depicted on the right. Each gray line represents one of the 35 trials, while the average response is shown in light blue. (B) Prediction of the PSP amplitudes (B) and PSC CVs (C) for the 130 possible pathways. (C) The combinations with bold borders indicate pathways that have been validated (panels C and D). Validation of PSP amplitudes (C) and PSC CVs (D). Dots represent mean values and whiskers the standard deviation of experimental (horizontal, in red) and model (vertical, in black) data. The dashed gray line indicates the diagonal (i.e., the target). Experimental values can be found in Ecker and colleagues. (PDF) [file pbio.3002861.s019.pdf]

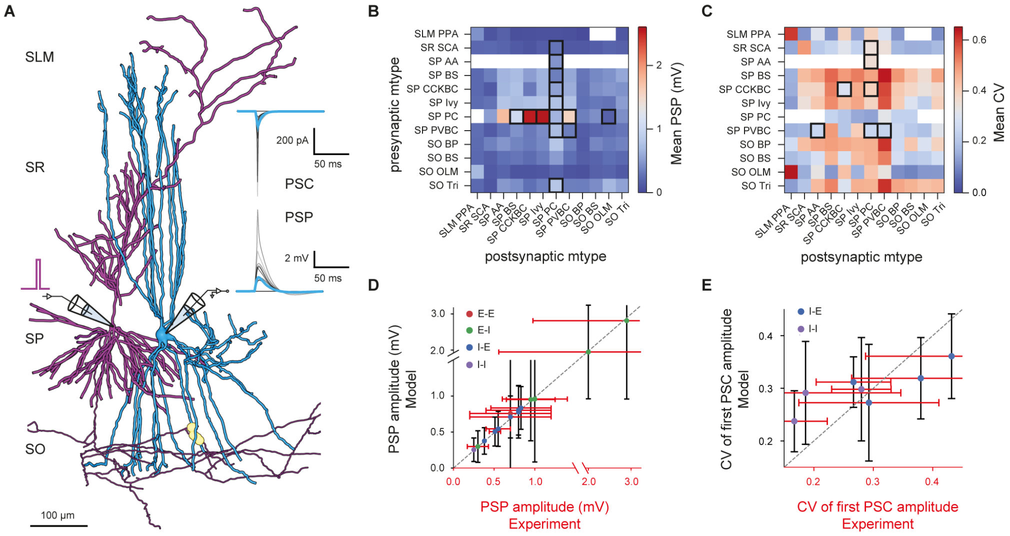

Figure S18: **Prediction and validation of synapses**. A. Example of *in silico* paired recording experiment between a PC (purple morphology) and a PVBC (light blue morphology), synapses between PC axon and PVBC dendrites in SO are depicted as yellow spheres. The presynaptic neuron is stimulated with a step current to elicit an action potential. Somatic recordings of EPSP and EPSC are depicted on the right. Each grey line represents one of the 35 trials, while the average response is shown in light blue. B. Prediction of the PSP amplitudes (B) and PSC CVs (C) for the 130 possible pathways. C. The combinations with bold borders indicate pathways that have been validated (panels C and D). Validation of PSP amplitudes (C) and PSC CVs (D). Dots represent mean values and whiskers the standard deviation of experimental (horizontal, in red) and model (vertical, in black) data. The dashed gray line indicates the diagonal (i.e., the target). Experimental values can be found in Ecker et al., 2020. (doi:10.1002/hipo.23220).
