# Supplementary material for: Community-based reconstruction and simulation of a full-scale model of the rat hippocampus CA1 region
Source: PLoS Biol. 2024 Nov 5;22(11):e3002861. doi: 10.1371/journal.pbio.3002861 (PMC11537418; doi:10.1371/journal.pbio.3002861)
Supplement: S19 Fig — (A) The distribution of the number of synapses per connection (1.0 ± 0.2 synapses/connection), the y-axis has a logarithmic scale. (B) The distribution of efferent synapses made by a single SC (34,135 ± 185 synapses). (C) Scheme of the workflow used for the fitting of SC → PC and SC → INT synapses. On the right, targets that are used for the 2 steps for the fitting of SC → PC and SC → INT. (D) Fitting results of SC → PC synapses. Each plot reports the distribution of one PSP feature (rise time, tau decay, and half-width in D1, D2, and D3, respectively) computed over the 10,000 pairs of pre and postsynaptic neurons. On top, experimental (in red) and model (in black) mean and standard deviation values are reported with a dot and a bar, respectively. (E) Fitting results of SC → INT synapses. E1 shows the average PC EPSPs in control conditions (black line) and when gabazine is applied (no GABA, gray line). The difference between the 2 is the IPSP induced by the feedforward inhibition (blue line). The inset shows the EPSP-IPSP latency, which is the difference between the onset of the IPSP and of the EPSP. E2. The EPSP-IPSP latency distribution of the 1,000 randomly selected PCs. Experimental values for panels D and E2 can be found respectively in S17 and S18 Tables. (PDF) [file pbio.3002861.s020.pdf]

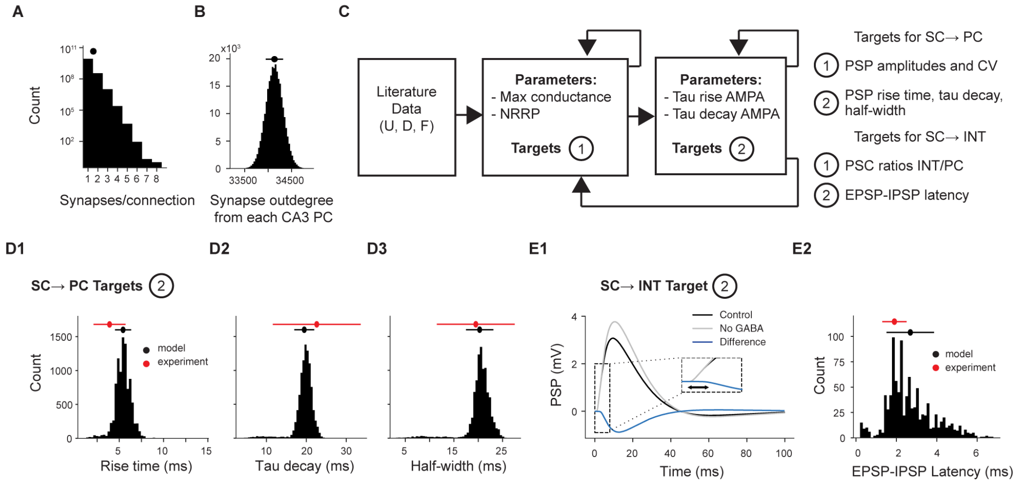

Figure S19: **Schaffer collaterals anatomy and physiology**. A. The distribution of the number of synapses per connection (1.0 ± 0.2 synapses/connection), the y-axis has a logarithmic scale. B. The distribution of efferent synapses made by a single SC (34,135 ± 185 synapses). C. Scheme of the workflow used for the fitting of SC → PC and SC → INT synapses. On the right, targets that are used for the two steps for the fitting of SC → PC and SC → INT. D. Fitting results of SC → PC synapses. Each plot reports the distribution of one PSP feature (rise time, tau decay, and half-width in D1, D2, and D3, respectively) computed over the 10,000 pairs of pre and postsynaptic neurons. On top, experimental (in red) and model (in black) mean and standard deviation values are reported with a dot and a bar, respectively. E. Fitting results of SC → INT synapses. E1 shows the average PC EPSPs in control conditions (black line) and when gabazine is applied (no GABA, gray line). The difference between the two is the IPSP induced by the feedforward inhibition (blue line). The inset shows the EPSP-IPSP latency, which is the difference between the onset of the IPSP and of the EPSP. E2. The EPSP-IPSP latency distribution of the 1000 randomly selected PCs. Experimental values for panels D and E2 can be found respectively in tables S17 and S18.
