# Supplementary material for: Community-based reconstruction and simulation of a full-scale model of the rat hippocampus CA1 region
Source: PLoS Biol. 2024 Nov 5;22(11):e3002861. doi: 10.1371/journal.pbio.3002861 (PMC11537418; doi:10.1371/journal.pbio.3002861)
Supplement: S20 Fig — (A) Relationship between spontanteous presynaptic release and postsynaptic events rates for pyramidal cells EPSPs (left) and IPSPs (right). (B) Relationship between calcium level and spontaneous presynaptic release rate for stratum pyramidale LFP responses peak frequency (left) and theta band power (right) shows weak theta power across all simulation experiments. (C–H) Example: 0.001 Hz presynaptic spontaneous release rate (cylinder circuit). (C–E) 2 mM calcium. (C) LFP and theta-band filtered LFP extracellular recordings from stratum pyramidale show irregular activity. (D) PSD shows multiple noisy peaks 1–20 Hz with the highest peak just below theta range. (E) Morlet complex wavelet spectrogram shows intermittent episodes of theta-band activity but these were associated with wide-range frequency response. (F–H) 1 mM calcium. (F) LFP and theta-band filtered LFP extracellular recordings show irregular activity but much smaller amplitude than for 2 mM calcium. (G) PSD shows multiple noisy peaks across a wider frequency range several orders of magnitude less than for 2 mM although the highest peak is just within theta-band. (H) Wavelet spectrogram shows a slightly more sustained period of theta and delta-band (1–3 Hz) activity but with more irregular, higher frequency events than 2 mM calcium. (PDF) [file pbio.3002861.s021.pdf]

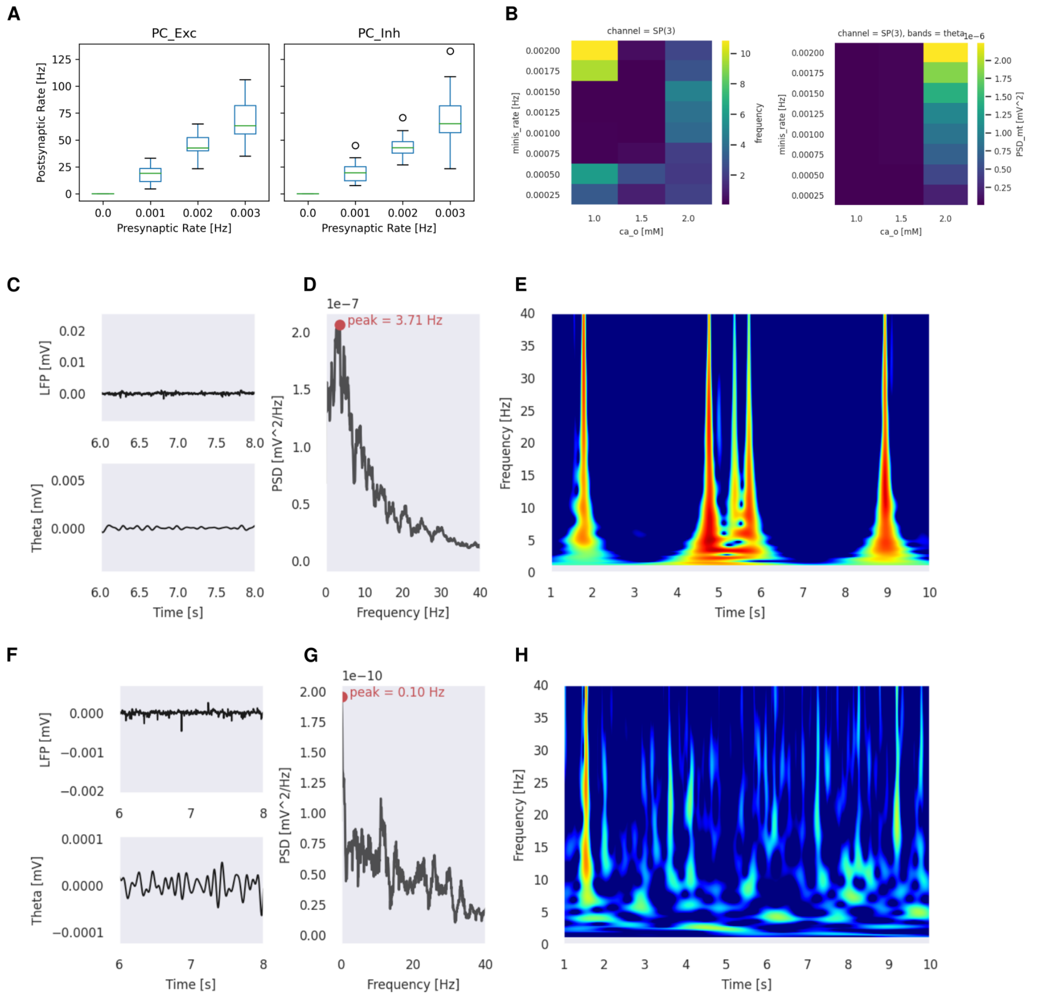

Figure S20: **Spontaneous synaptic release alone did not generate sustained theta oscillations in the CA1 model**. A. Relationship between spontanteous presynaptic release and postsynaptic events rates for pyramidal cells EPSPs (left) and IPSPs (right). B. Relationship between calcium level and spontaneous presynaptic release rate for stratum pyramidale LFP responses peak frequency (left) and theta band power (right) shows weak theta power across all simulation experiments. C-H. Example: 0.001 Hz presynaptic spontaneous release rate (cylinder circuit). C-E. $2\,\mathrm{mM}$ calcium. C. LFP and theta-band filtered LFP extracellular recordings from stratum pyramidale show irregular activity. D. Power spectral density (PSD) shows multiple noisy peaks 1-20 Hz with the highest peak just below theta range. E. Morlet complex wavelet spectrogram shows intermittent episodes of theta-band activity but these were associated with wide-range frequency response. F-H. $1\,\mathrm{mM}$ calcium. F. LFP and theta-band filtered LFP extracellular recordings show irregular activity but much smaller amplitude than for $2\,\mathrm{mM}$ calcium. G. PSD shows multiple noisy peaks across a wider frequency range several orders of magnitude less than for $2\,\mathrm{mM}$ although the highest peak is just within theta-band. H. Wavelet spectrogram shows a slightly more sustained period of theta and delta-band (1-3 Hz) activity but with more irregular, higher frequency events than $2\,\mathrm{mM}$ calcium.
