# Supplementary material for: Community-based reconstruction and simulation of a full-scale model of the rat hippocampus CA1 region
Source: PLoS Biol. 2024 Nov 5;22(11):e3002861. doi: 10.1371/journal.pbio.3002861 (PMC11537418; doi:10.1371/journal.pbio.3002861)
Supplement: S21 Fig — For each panel: LFP and theta filtered LFP traces (far left), PSD (middle left), wavelet spectrogram (middle right), CSD (far right) (A). Poisson rate 0.05 Hz. PSD shows broad and noisy peak power located in beta frequency band (13–25 Hz) (B). Poisson rate 0.10 Hz. (C). Poisson rate 0.60 Hz. PSD shows little power within theta and beta band. (PDF) [file pbio.3002861.s022.pdf]

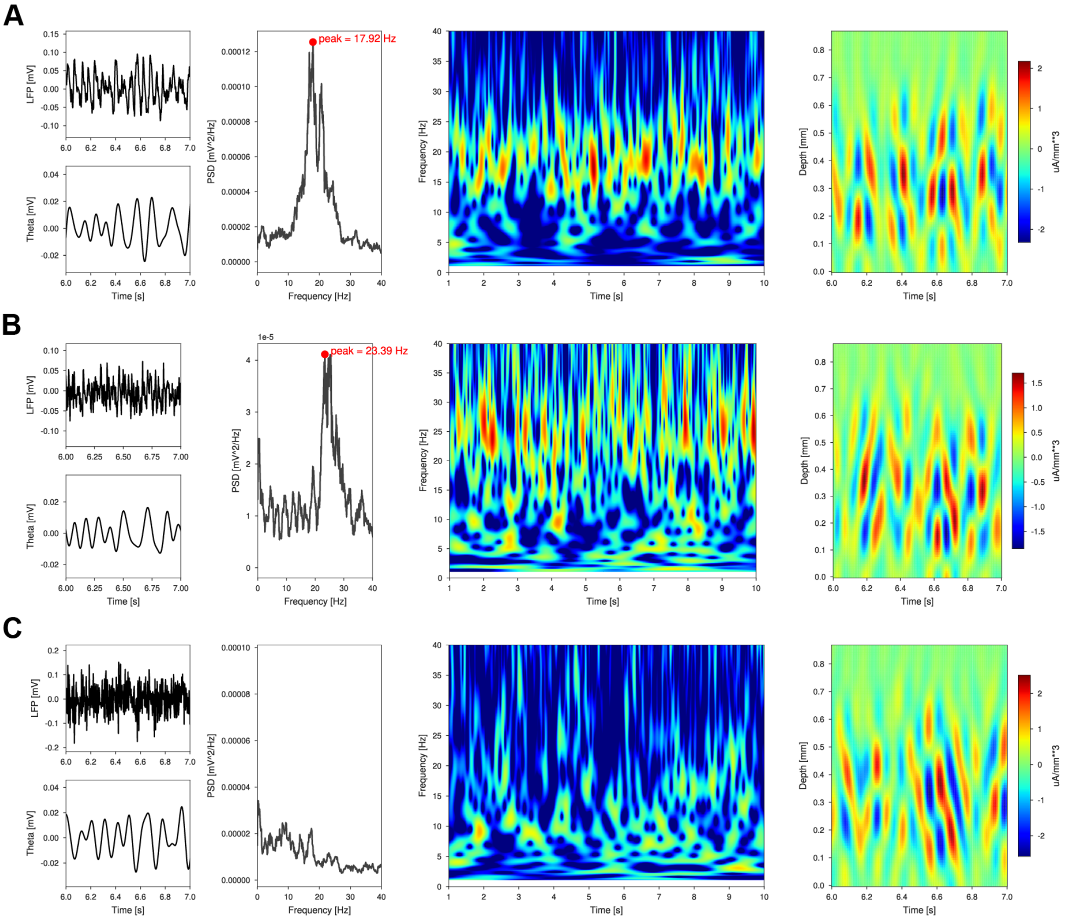

Figure S21: **Extrinsic random synaptic activity at low levels generated noisy beta but not theta oscillatory activity in the CA1 model**. For each panel: LFP and theta filtered LFP traces (far left), PSD (middle left), wavelet spectrogram (middle right), CSD (far right) A. Poisson rate 0.05 Hz. PSD shows broad and noisy peak power located in beta frequency band (13-25 Hz) B. Poisson rate 0.10 Hz. C. Poisson rate 0.60 Hz. PSD shows little power within theta and beta band.
