# Supplementary material for: Community-based reconstruction and simulation of a full-scale model of the rat hippocampus CA1 region
Source: PLoS Biol. 2024 Nov 5;22(11):e3002861. doi: 10.1371/journal.pbio.3002861 (PMC11537418; doi:10.1371/journal.pbio.3002861)
Supplement: S22 Fig — (A) Sample histograms of CA3 input spike time input (top) and output poststimulus response of CA1 pyramidal and interneurons for a range of CA3 Poisson rates. (B) Spiking input–output relationship between CA3 input spike rate and mean output spiking rates of pyramidal cells (left) and interneuron types (right). (C) Pyramidal cell cross-correlation histograms (CCH) for different CA3 input spike rates both lack evidence of oscillatory response to randomly timed extrinsic afferent EPSPs. (PDF) [file pbio.3002861.s023.pdf]

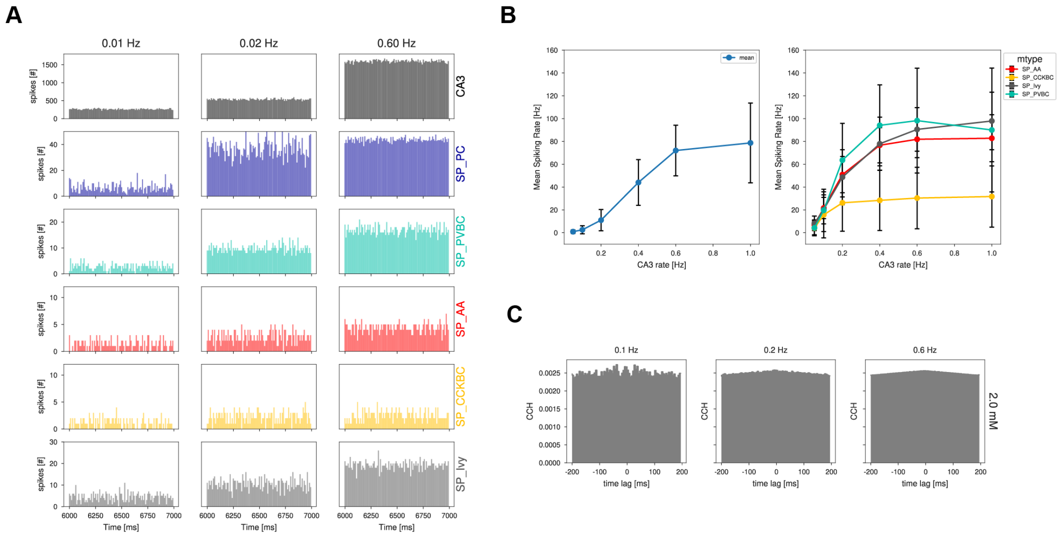

Figure S22: **Extrinsic random synaptic activity fails to induce oscillatory spiking response in the CA1 circuit**. A. Sample histograms of CA3 input spike time input (top) and output post-stimulus response of CA1 pyramidal and interneurons for a range of CA3 Poisson rates. B. Spiking input-output relationship between CA3 input spike rate and mean output spiking rates of pyramidal cells (left) and interneuron types (right). C. Pyramidal cell cross-correlation histograms (CCH) for different CA3 input spike rates both lack evidence of oscillatory response to randomly timed extrinsic afferent EPSPs.
