# Supplementary material for: Community-based reconstruction and simulation of a full-scale model of the rat hippocampus CA1 region
Source: PLoS Biol. 2024 Nov 5;22(11):e3002861. doi: 10.1371/journal.pbio.3002861 (PMC11537418; doi:10.1371/journal.pbio.3002861)
Supplement: S23 Fig — (A) At 120% rheobase, for 1 mM calcium sparse and uncoordinated spiking but at oscillatory peaks occur at 2 mM in pyramidal cell cross-correlation histograms (CCH) in all sizes of circuit. (B) For 2 mM calcium, increasing level of tonic depolarization degrades the strength of oscillation for all sizes of circuit suggesting the resonance effect is limited to a narrow range of tonic depolarization. (PDF) [file pbio.3002861.s024.pdf]

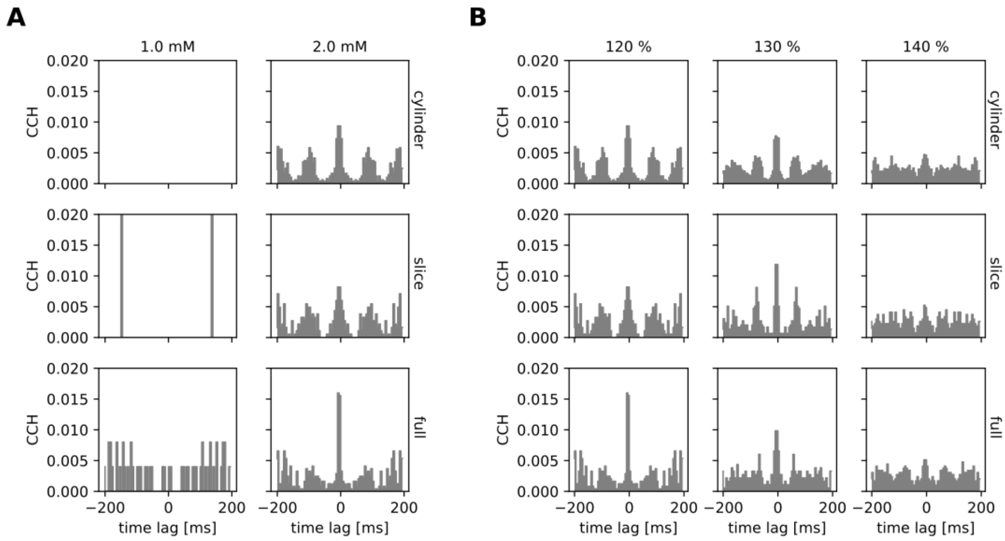

Figure S23: **Tonic depolarisation induces spiking oscillations at $2\,\mathrm{mM}$ but not $1\,\mathrm{mM}$ calcium for a narrow percentage of relative rheobase across circuit scales.** A. At 120% rheobase, for $1\,\mathrm{mM}$ calcium sparse and uncoordinated spiking but at oscillatory peaks occur at $2\,\mathrm{mM}$ in pyramidal cell cross-correlation histograms (CCH) in all sizes of circuit. B. For $2\,\mathrm{mM}$ calcium, increasing level of tonic depolarisation degrades the strength of oscillation for all sizes of circuit suggesting the resonance effect is limited to a narrow range of tonic depolarisation.
