# Supplementary material for: Community-based reconstruction and simulation of a full-scale model of the rat hippocampus CA1 region
Source: PLoS Biol. 2024 Nov 5;22(11):e3002861. doi: 10.1371/journal.pbio.3002861 (PMC11537418; doi:10.1371/journal.pbio.3002861)
Supplement: S24 Fig — Example: 2 mM calcium, 120% rheobase depolarization (recording electrode in SP). For each panel: LFP and theta filtered LFP traces (far left), PSD (middle left), Wavelet Spectrogram (middle right), CSD (far right). (A) Cylinder circuit. (B) Slice circuit. (C) Full circuit. (PDF) [file pbio.3002861.s025.pdf]

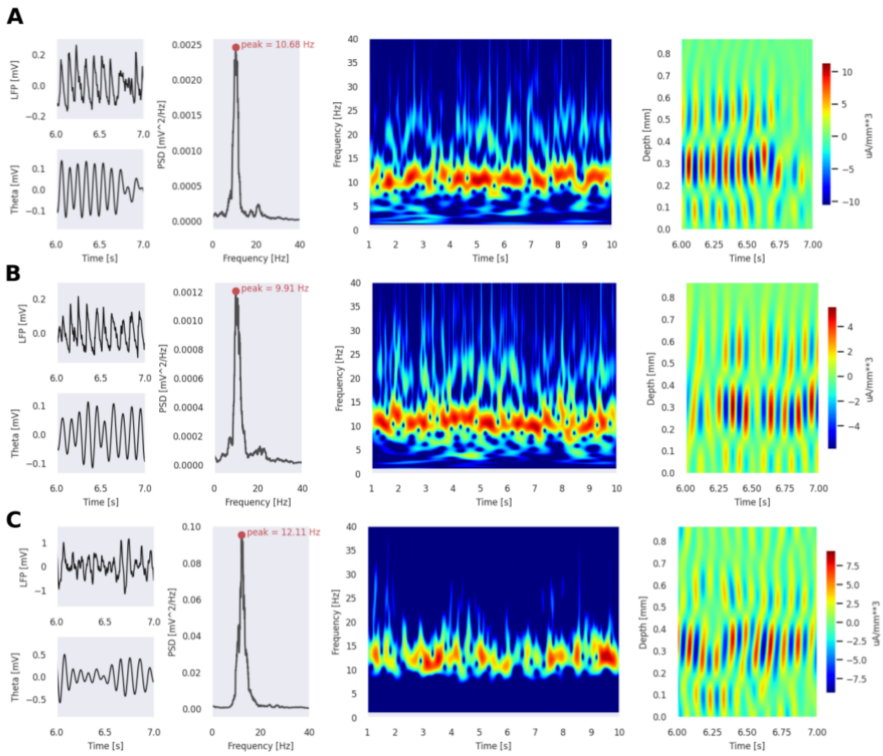

Figure S24: **Tonic depolarisation generates theta-band oscillations across circuit scales at 2 mM extracellular Calcium concentration**.Example: 2 mM Calcium, 120% rheobase depolarisation (recording electrode in SP). For each panel: LFP and theta filtered LFP traces (far left), PSD (middle left), Wavelet Spectrogram (middle right), CSD (far right). A. Cylinder circuit. B. Slice circuit. C. Full circuit.
