# Supplementary material for: Community-based reconstruction and simulation of a full-scale model of the rat hippocampus CA1 region
Source: PLoS Biol. 2024 Nov 5;22(11):e3002861. doi: 10.1371/journal.pbio.3002861 (PMC11537418; doi:10.1371/journal.pbio.3002861)
Supplement: S25 Fig — Top panel: 1 s example traces of 1–80 Hz filtered LFP from stratum pyramidal SP (3) electrode with estimated locations of theta peaks and troughs shown. Bottom panel: estimated rise times (trough to peak time) (far left), estimated decay times (peak to trough time) (middle left), calculated theta wave asymmetry index (middle right), and scatter plot of rise and decay times (n = 71 total waves). Example: 8 Hz signal frequency, 0.4 Hz cell frequency, 2 mM calcium, full circuit. (PDF) [file pbio.3002861.s026.pdf]

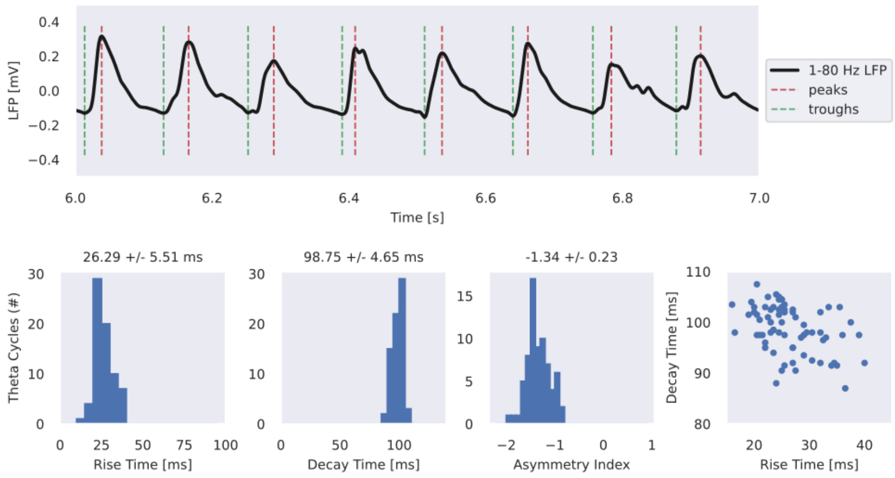

Figure S25: **LFP theta waves are highly asymmetric for an extrinsic excitatory oscillatory stimulus.** Top panel: 1 second example traces of 1-80 Hz filtered LFP from stratum pyramidal SP (3) electrode with estimated locations of theta peaks and troughs shown. Bottom panel: estimated rise times (trough to peak time) (far left), estimated decay times (peak to trough time) (middle left), calculated theta wave asymmetry index (middle right), and scatter plot of rise and decay times (n $=$ 71 total waves). Example: 8 Hz signal frequency, 0.4 Hz cell frequency, $2\,\mathrm{mM}$ calcium, full circuit.
