# Supplementary material for: Community-based reconstruction and simulation of a full-scale model of the rat hippocampus CA1 region
Source: PLoS Biol. 2024 Nov 5;22(11):e3002861. doi: 10.1371/journal.pbio.3002861 (PMC11537418; doi:10.1371/journal.pbio.3002861)
Supplement: S26 Fig — Example: 2 mM calcium, 0.4 Hz cell frequency, and 8 Hz modulation frequency. (PDF) [file pbio.3002861.s027.pdf]

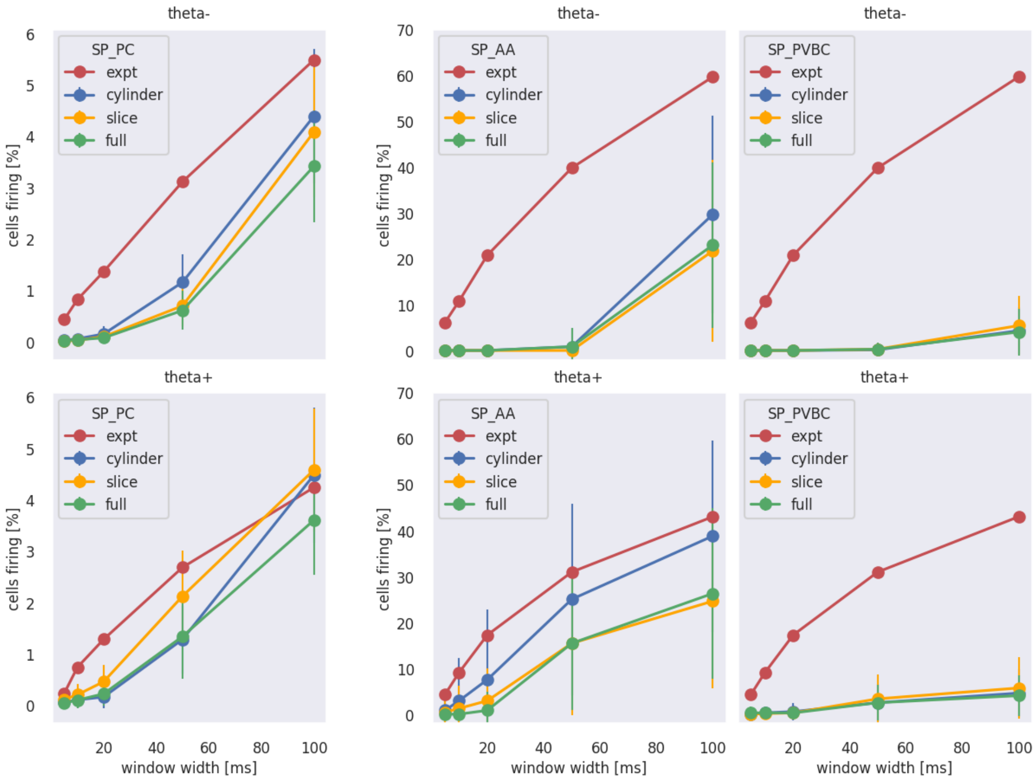

Figure S26: **Population synchrony of pyramidal cells does not match experimental theta trough ('theta-') and fast-spiking interneurons recruitment lower than experimental levels although better for SP_AA than SP_PVBC independent of circuit scale**. Example: $2\,\mathrm{mM}$ calcium, 0.4 Hz cell frequency and 8 Hz modulation frequency.
