# Supplementary material for: Community-based reconstruction and simulation of a full-scale model of the rat hippocampus CA1 region
Source: PLoS Biol. 2024 Nov 5;22(11):e3002861. doi: 10.1371/journal.pbio.3002861 (PMC11537418; doi:10.1371/journal.pbio.3002861)
Supplement: S27 Fig — Top panel: 1 s example traces of 1–80 Hz filtered LFP from str pyramidal SP(3) electrode with estimated locations of theta peaks and troughs shown. Bottom panel: estimated rise times (trough to peak time) (far left), estimated decay times (peak to trough time) (middle left), calculated theta wave asymmetry index (middle right), and scatter plot of rise and decay times (n = 79 total waves). Example: 8 Hz signal frequency, 1 μM ACh, depolarisation = 120%, 2 mM calcium, cylinder circuit. (PDF) [file pbio.3002861.s028.pdf]

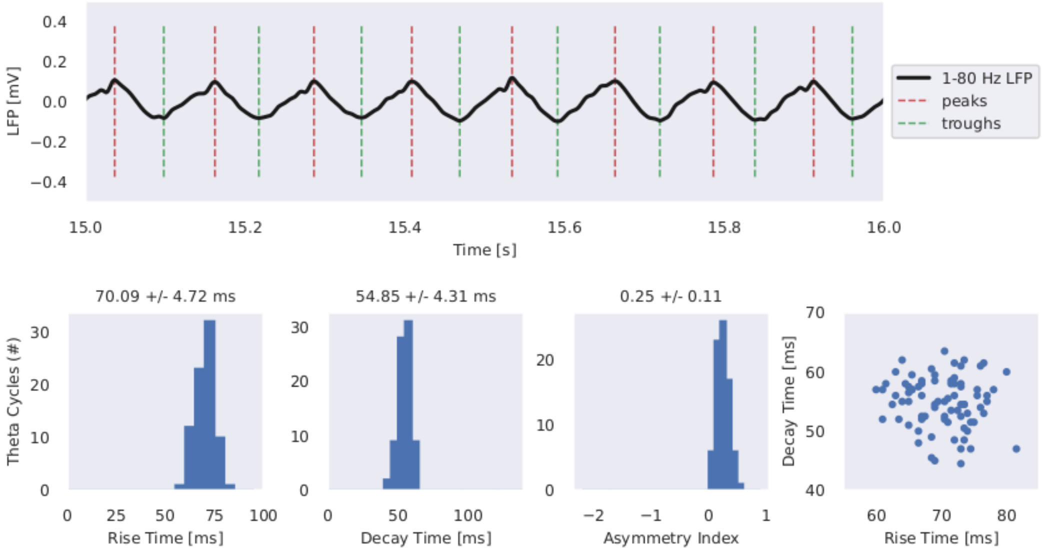

Figure S27: **LFP theta waves are nearly symmetric for an extrinsic inhibitory oscillatory stimulus**.Top panel: 1 second example traces of 1-80 Hz filtered LFP from str pyramidal SP(3) electrode with estimated locations of theta peaks and troughs shown. Bottom panel: estimated rise times (trough to peak time) (far left), estimated decay times (peak to trough time) (middle left), calculated theta wave asymmetry index (middle right), and scatter plot of rise and decay times (n = 79 total waves). Example: 8 Hz signal frequency, $1\,\mu\mathrm{M}$ ACh, depolarisation = 120%, $2\,\mathrm{mM}$ Calcium, cylinder circuit.
