# Supplementary material for: Community-based reconstruction and simulation of a full-scale model of the rat hippocampus CA1 region
Source: PLoS Biol. 2024 Nov 5;22(11):e3002861. doi: 10.1371/journal.pbio.3002861 (PMC11537418; doi:10.1371/journal.pbio.3002861)
Supplement: S28 Fig — Example: 120% depolarisation, 1 μM ACh. (PDF) [file pbio.3002861.s029.pdf]

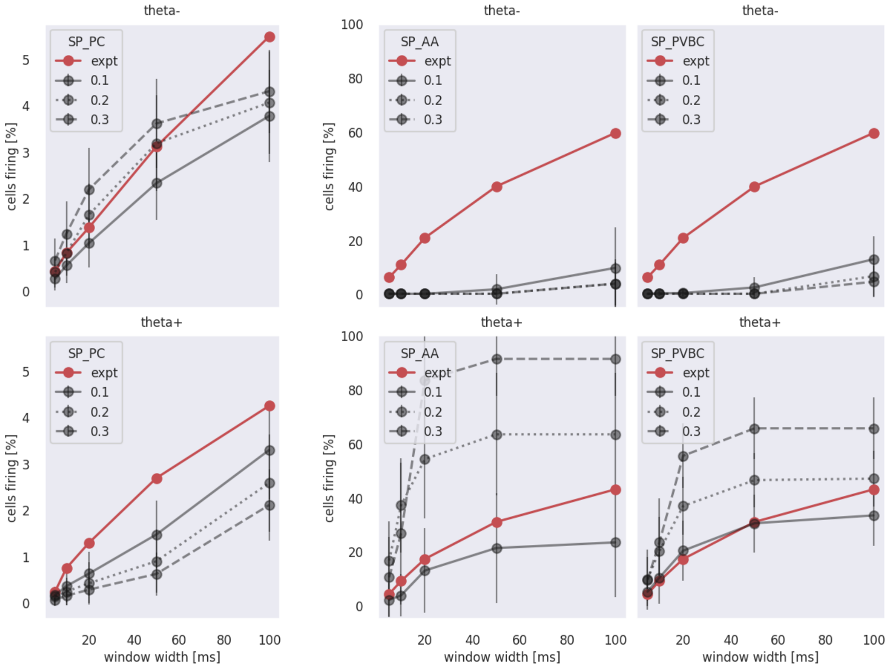

Figure S28: **Population synchrony of pyramidal cells matches experimental theta trough ("theta-") and fast-spiking interneurons SP_AA and SP_PVBC experimental matches theta peak ("theta+") for increased stimulus amplitudes**. Example: 120% depolarisation, $1\,\mu\mathrm{M}$ ACh.
