# Supplementary material for: Community-based reconstruction and simulation of a full-scale model of the rat hippocampus CA1 region
Source: PLoS Biol. 2024 Nov 5;22(11):e3002861. doi: 10.1371/journal.pbio.3002861 (PMC11537418; doi:10.1371/journal.pbio.3002861)
Supplement: S29 Fig — Gamma oscillation (31 Hz) robustly propagates to CA1 when there is a sufficient amount of SC input. (A) Shows wavelet spectrograms of LFPs recorded in simulations where CA1 neurons were driven by oscillating input via different numbers of Schaffer collaterals, but no CCh was applied. (B) Shows the wavelet spectrograms of the same simulations, but with simulating the effect of 10 μM CCh. A wide range of the number of SC inputs is able to induce strong gamma oscillation in CA1 both in the case of CCh application and without CCh, but CCh seems to increase the number of inputs needed for stable gamma oscillation, probably due to its weakening effect on synapses. (PDF) [file pbio.3002861.s030.pdf]

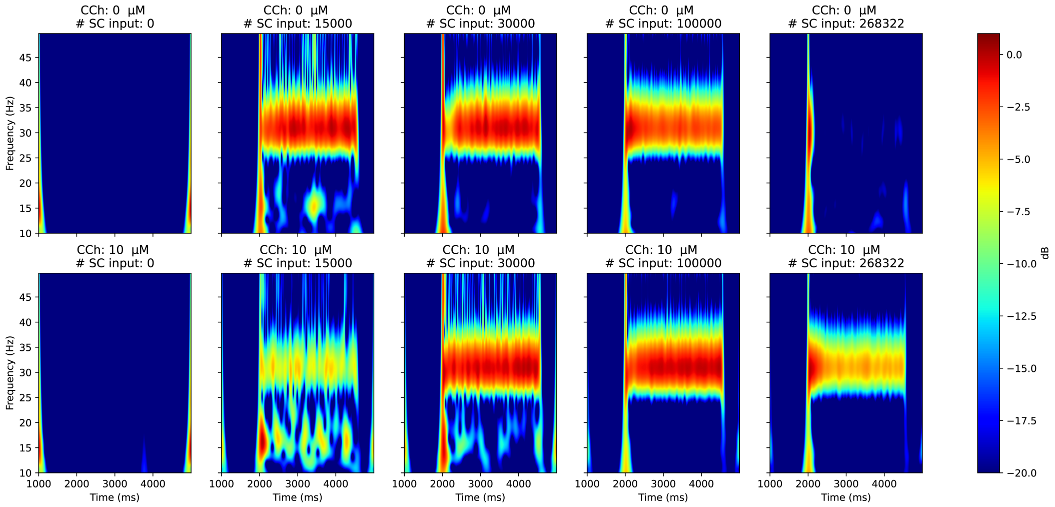

Figure S29: **Propagation of gamma oscillation from CA3 to CA1**. Gamma oscillation (31 Hz) robustly propagates to CA1 when there is a sufficient amount of SC input. (A) shows wavelet spectrograms of LFPs recorded in simulations where CA1 neurons were driven by oscillating input via different numbers of Schaffer Collaterals, but no CCh was applied. (B) shows the wavelet spectrograms of the same simulations, but with simulating the effect of $10\,\mu\mathrm{M}$ CCh. A wide range of the number of SC inputs is able to induce strong gamma oscillation in CA1 both in the case of CCh application and without CCh, but CCh seems to increase the number of inputs needed for stable gamma oscillation, probably due to its weakening effect on synapses.
