# Supplementary material for: Community-based reconstruction and simulation of a full-scale model of the rat hippocampus CA1 region
Source: PLoS Biol. 2024 Nov 5;22(11):e3002861. doi: 10.1371/journal.pbio.3002861 (PMC11537418; doi:10.1371/journal.pbio.3002861)
Supplement: S1 Table — (PDF) [file pbio.3002861.s031.pdf]

| Abbreviation | Full name |
|---|---|
| ACh | acetylcholine |
| AMPA | $\alpha$-amino-3-hydroxy-5-methyl-4-isoxazolepropionic acid |
| bAC | bursting accommodating |
| BP | back-projecting m-type neuron |
| BPAP | back-propagating action potential |
| CA | cornu ammonis |
| CA1 | field CA1 |
| CA3 | field CA3 |
| CCh | carbachol |
| cACpyr | classical accommodating for pyramidal cells |
| cAC | classical accommodating for interneurons |
| CB1R | cannabinoid receptor type 1 |
| CCK+ | cholecystokinin-positive |
| cNAC | classical non-accommodating |
| CSD | current source density |
| CV | coefficient of variation |
| CWT | continuous wavelet transform |
| DG | dentate gyrus |
| e-feature | electrophysiological feature |
| e-type | electrical type |
| EPSC | excitatory postsynaptic current |
| EPSP | excitatory postsynaptic potential |
| GABA | gamma-aminobutyric acid or $\gamma$-aminobutyric acid |
| GABA_A *R* | GABA_A receptor |
| HCN | hyperpolarization-activated cyclic nucleotide–gated channel |
| I_depol | depolarizing current |
| I_h | nonspecific hyperpolarization-activated cation current |
| I-O | input-output |
| INT | interneurons |
| IPSP | Inhibitory postsynaptic potential |
| KYNA | kynurenic acid |
| LFP | local field potential |
| m-type | morphological type |
| me-type | morpho-electrical type |
| minis/mPSP | miniature postsynaptic potentials |
| MOOC | massive online open course |
| MS | medial septum |
| MS OFF | medial septum inactivated stimulus condition |
| MS ON | medial septum activated stimulus condition |
| mM | millimolar = $10^{-3}$ |

| | |
|---|---|
| $\mu$M | micromolar = $10^{-6}$ |
| N.B. | nota bene |
| NMDA | N-methyl-d-aspartate |
| N_RRP | number (size) of the readily releasable pool (#vesicles) |
| nS | nanoSiemens |
| OLM | oriens-lacunosum moleculare m-type neuron |
| PC | pyramidal cell |
| PP | perforant path |
| PPA | perforant path-associated cell |
| PSC | postsynaptic current |
| PSD | power spectral density |
| PSP | postsynaptic potential |
| PV+ | parvalbumin-positive |
| PVBC | parvalbumin-positive basket m-type neuron |
| REM | rapid eye movement |
| SC | Schaffer collateral |
| SCA | Schaffer collateral associated neuron |
| SD rat | Sprague Dawley rat |
| SLM | stratum lacunosum moleculare |
| SLM_PPA | perforant path-associated m-type neuron with soma located in SLM |
| SO | stratum oriens |
| SO_BP | back-projecting m-type neuron with soma located in SO |
| SO_BS | bistratified m-type neuron with soma located in SO |
| SO_OLM | oriens-lacunosum moleculare m-type neuron with soma located in SO |
| SO_Tri | trilaminar m-type neuron with soma located in SO |
| SP | stratum pyramidale |
| SP_AA | axoaxonic m-type neuron with soma located in SP |
| SP_BS | bistratified m-type neuron with soma located in SP |
| SP_CCKBC | cholecystokinin-positive basket cell m-type neuron with soma in SP |
| SP_Ivy | ivy m-type neuron with soma located in SP |
| SP_PC | pyramidal m-type neuron with soma located in SP |
| SP_PVBC | parvalbumin-positive basket m-type neuron with soma located in SP |
| SR | stratum radiatum |
| SR_SCA | Schaffer collateral associated m-type neuron with soma located in SR |
| SST | somatostatin |
| STD | stardard deviation |
| STP | short-term plasticity |
| STTC | spike time tiling coefficient |
| Tri | trilaminar m-type neuron |
| U_SE | release probability of neurotransmitters |
| W rat | Wistar rat |

Table S1: **List of abbreviations and acronyms**
