# Supplementary material for: Community-based reconstruction and simulation of a full-scale model of the rat hippocampus CA1 region
Source: PLoS Biol. 2024 Nov 5;22(11):e3002861. doi: 10.1371/journal.pbio.3002861 (PMC11537418; doi:10.1371/journal.pbio.3002861)
Supplement: S2 Table — For reasons of space, this is a non-exhaustive list of features. (PDF) [file pbio.3002861.s032.pdf]

	This paper	Yu et al (2020)	Bezaire et al (2016)	Cutsuridis et al (2010)	Traub et al (2000)	Traub et al (1992)
Regions	CA1	EC, DG, CA3	CA1	CA1	CA3	CA3
Circuit scales	full-scale	reduced full-scale	full-scale	microcircuit	microcircuit	microcircuit
Atlas	3D layered atlas	2D flat map	layered 3D slab	no	no	no
Neuron types	pyramidal	granule cells	pyramidal	pyramidal	pyramidal	pyramidal
	axoaxonic	pyramidal	axoaxonic	axoaxonic	axoaxonic	interneurons
	basket (CCK+)	basket	basket (CCK+)	basket	basket	
	basket (PV+)		basket (PV+)	bistratified	bistratified	
	bistratified		bistratified	OLM	OLM	
	ivy		ivy			
	OLM		neurogliaform			
	trilaminar		OLM			
	PPA		SCA			
	SCA					
Short-term synaptic plasticity	yes	no	no	no	no	no
Neurotransmitters	AMPA	AMPA	AMPA	AMPA	AMPA	AMPA
	NMDA	NMDA		NMDA		
	GABA-A	GABA-A	GABA-A	GABA-A	GABA-A	GABA-A
			GABA-B	GABA-B		GABA-B
Spontaneous synaptic release (minis)	yes	no	no	no	no	no
Neuromodulation	yes	no	no	no	yes	yes
LFP	yes	no	yes	no	no	no

Table S2: **Key feature comparison of realistic large-scale hippocampal network models with multicompartmental HH model neurons.** For reasons of space, this is a non-exhaustive list of features.
