# Supplementary material for: Community-based reconstruction and simulation of a full-scale model of the rat hippocampus CA1 region
Source: PLoS Biol. 2024 Nov 5;22(11):e3002861. doi: 10.1371/journal.pbio.3002861 (PMC11537418; doi:10.1371/journal.pbio.3002861)
Supplement: S3 Table — Values were obtained combining multiple data sets. See section Cell composition of Methods for details. (PDF) [file pbio.3002861.s033.pdf]

| MType | Percentage | N. cells | Density $\mu m^{-3}$ |
|---|---|---|---|
| SLM_PPA | 0.215 | 1008 | 313.652 |
| SR_SCA | 0.176 | 823 | 120.285 |
| SP_PC | 89 | 416842 | 264000 |
| SP_Ivy | 3.870 | 18127 | 11480.167 |
| SP_PVBC | 2.429 | 11378 | 7206.052 |
| SP_BS | 0.738 | 3457 | 2189.180 |
| SP_AA | 0.646 | 3025 | 1915.533 |
| SP_CCKBC | 1.581 | 7407 | 4691.101 |
| SO_OLM | 0.720 | 3374 | 700.382 |
| SO_BP | 0.083 | 391 | 81.142 |
| SO_Tri | 0.308 | 1440 | 298.944 |
| SO_BS | 0.233 | 1090 | 226.343 |
| Total | 100 | 468362 | |

Table S3: **Cell composition, counts and densities.** Values were obtained combining multiple datasets. See Section Cell composition of Methods for details.
