# Supplementary material for: Community-based reconstruction and simulation of a full-scale model of the rat hippocampus CA1 region
Source: PLoS Biol. 2024 Nov 5;22(11):e3002861. doi: 10.1371/journal.pbio.3002861 (PMC11537418; doi:10.1371/journal.pbio.3002861)
Supplement: S4 Table — Percentage of electrical types (e-types) for each morphological type (m-type). Last column indicates the number of traces used to estimate the percentages. See section Morpho-electrical compositions of Methods for more details. BS indicates both SP_BS and SO_BS. (PDF) [file pbio.3002861.s034.pdf]

M-type	cNAC	cAC	bAC	N. traces
SLM_PPA	0.00%	0.00%	100.00%	1
SR_SCA	0.00%	100.00%	0.00%	2
SP_AA	0.00%	0.00%	100.00%	3
BS	75.00%	0.00%	25.00%	10
SP_CCKBC	0.00%	100.00%	0.00%	-
SP_Ivy	33.33%	0.00%	66.67%	3
SP_PC	0.00%	100.00%	0.00%	106
SP_PVBC	70.00%	0.00%	30.00%	10
SO_BP	0.00%	100.00%	0.00%	3
SO_OLM	0.00%	100.00%	0.00%	11
SO_Tri	0.00%	100.00%	0.00%	4

Table S4: **Morpho-electrical composition.** Percentage of electrical types (e-types) for each morphological type (m-type). Last column indicates the number of traces used to estimate the percentages. See Section Morpho-electrical compositions of Methods for more details. BS indicates both SP_BS and SO_BS.
