# Supplementary material for: Community-based reconstruction and simulation of a full-scale model of the rat hippocampus CA1 region
Source: PLoS Biol. 2024 Nov 5;22(11):e3002861. doi: 10.1371/journal.pbio.3002861 (PMC11537418; doi:10.1371/journal.pbio.3002861)
Supplement: S5 Table — (PDF) [file pbio.3002861.s035.pdf]

| Cell type | Region | Species[1] | Age | Mean ($10^3/mm^3$) | N. animals | STD | SEM | Reference |
|---|---|---|---|---|---|---|---|---|
| All | CA1 | W rat | 9-10 w | 35.2 | 5 | 1.1 | 0.5 | [1] |
| SO | SO | W rat | 9-10 w | 11.3 | 5 | 2.0 | 0.9 | [1] |
| SP | SP | W rat | 9-10 w | 272.4 | 5 | 32.0 | 14.3 | [1] |
| SR-SLM | SR+SLM | W rat | 9-10 w | 1.9 | 5 | 0.7 | 0.3 | [1] |
| SP_PC | SP | W rat | 9-10 w | 264 | 5 | 32.6 | 14.6 | [1] |

Table S5: **Neuron density validation.**

[1] SD rat: Sprague Dawley rat, W rat: Wistar rat, LE rat: Long–Evans rat, G pig: Guinea pig.
