# Supplementary material for: Community-based reconstruction and simulation of a full-scale model of the rat hippocampus CA1 region
Source: PLoS Biol. 2024 Nov 5;22(11):e3002861. doi: 10.1371/journal.pbio.3002861 (PMC11537418; doi:10.1371/journal.pbio.3002861)
Supplement: S6 Table — (PDF) [file pbio.3002861.s036.pdf]

Mtype	Dendrites	Tuft	Axon
SLM_PPA	SLM		2/3 SR-SLM
SR_SCA	SO-SLM		SO-SLM
SP_PC	SO-SLM	SLM	
SP_Ivy	SO-SR		2/3 SO-1/3 SR
SP_BS	SO-SR		SO-SR
SP_*BC	SO-SLM	SLM	2/3 SO-1/4 SR
SP_AA	SO-SLM	SLM	1/2 SO-SP
SO_Tri	SO-SR		SO-SR
SO_OLM			SLM
SO_BS	SO		SO-SR
SO_BP	SO-SLM		SO-SLM

Table S6: **Placement "optional" rules.**
