# Supplementary material for: Community-based reconstruction and simulation of a full-scale model of the rat hippocampus CA1 region
Source: PLoS Biol. 2024 Nov 5;22(11):e3002861. doi: 10.1371/journal.pbio.3002861 (PMC11537418; doi:10.1371/journal.pbio.3002861)
Supplement: S7 Table — (PDF) [file pbio.3002861.s037.pdf]

Mtype	Region	Species[1]	Weight	Mean $(100 \mu m^{-1})$	N. cells	STD	SEM	Reference
SO_BS	CA1	SD rat	250-350 g	21.0	1[2]	5.6[3]	5.6	[1]
SO_BP	CA1	SD rat	250-350 g	24.8	1	4.13	4.13	[2][4]
SP_PC	CA1	W rat	180-200 g	12.41	4	6.02	3.01	[3] [4][5]
SO_Tri	CA1	SD rat	250-350 g	28.2	1	4.9[3]	4.9	[1]
SP_PVBC	CA1	SD rat	250-350 g	22.6	4	3.9[3]	1.95	[1]
SO_OLM	CA1	SD rat	250-350 g	26.6	2	4.0[3]	2.83	[1]

Table S7: **Bouton density.**

[1] SD rat: Sprague Dawley rat, W rat: Wistar rat, LE rat: Long–Evans rat, G pig: Guinea pig.

[2] The authors define the sample size (n) probably as the number of sampled segments rather than the number of animals

[3] the authors do not specify if this is std or SEM. Anyway, in a previous publication [2] they used std. We can assume they are std

[4] the authors do not mention species and age in the paper. Anyway, a later paper [1] mentions the result so we assume they use the same method

[5] Bouton density for PC was computed as a weighted mean of the bouton density per branch order [3]. Then we analyzed the axon, calculated the total length of each branch order, and used this to weight the bouton density.

References

[1] Sik A, Penttonen M, Ylinen A, Buzsáki G. Hippocampal CA1 interneurons: an in vivo intracellular labeling study;15(10):6651–6665. doi:10.1523/JNEUROSCI.15-10-06651.1995.

[2] Sik A, Tamamaki N, Freund TF. Complete Axon Arborization of a Single CA3 Pyramidal Cell in the Rat Hippocampus, and its Relationship With Postsynaptic Parvalbumin-containing Interneurons;5(12):1719–1728. doi:10.1111/j.1460-9568.1993.tb00239.x.

[3] Esclapez M, Hirsch JC, Ben-Ari Y, Bernard C. Newly formed excitatory pathways provide a substrate for hyperexcitability in experimental temporal lobe epilepsy;408(4):449–460. doi:10.1002/(SICI)1096-9861(19990614)408:4<449::AID-CNE1>3.0.CO;2-R.

[4] Bezaire MJ, Soltesz I. Quantitative assessment of CA1 local circuits: Knowledge base for interneuron-pyramidal cell connectivity: Quantitative Assessment Of Ca1 Local Circuits;23(9):751–785. doi:10.1002/hipo.22141.
