# Supplementary material for: Community-based reconstruction and simulation of a full-scale model of the rat hippocampus CA1 region
Source: PLoS Biol. 2024 Nov 5;22(11):e3002861. doi: 10.1371/journal.pbio.3002861 (PMC11537418; doi:10.1371/journal.pbio.3002861)
Supplement: S8 Table — (PDF) [file pbio.3002861.s038.pdf]

Pre	Post	Species[1]	Age	Weight	Slice thickness (μm)	Distance	n	N	p	Reference
PC	PC	SD rat	-	100-180 g	400-500	-	11	989	0.011	[1]
PC	OLM	SD rat	-	90-150 g	450-500	-	12	36	0.333	[2]
PVBC	PC	SD rat	-	120-200 g	450	-	49	167	0.293	[3]
PC	PVBC	SD rat	-	120-200 g	450	-	16	124	0.129	[3]
BS	PC	SD rat	-	120-200 g	450	-	2	6	0.333	[3]
PC	BS	SD rat	-	120-200 g	450	-	2	4	0.500	[3]
CCKBC	PC	SD rat	-	120-200 g	450	-	21	88	0.239	[3]
PC	CCKBC	SD rat	-	120-200 g	450	-	8	81	0.099	[3]
CCKBS	PC	SD rat	-	120-200 g	450	-	5	36	0.139	[3]
PC	CCKBS	SD rat	-	120-200 g	450	-	6	35	0.171	[3]
PC	SCA	SD rat	-	120-200 g	450	-	0	32	0	[3]
Ivy	PC	W rat	-	140-200 g	450	-	3	5	0.600	[4]
Ivy	Ivy	W rat	-	140-200 g	450	-	1	4	0.250	[4]
INT	PC	W rat	-	140-200 g	450	-	6	21	0.286	[4]
BC	PC	SD rat	-	120-200 g	450-500	-	57	263	0.217	[5]
BC	PC	SD rat	-	120-200 g	-	50-100	46	89	0.517	[5]
BC	PC	SD rat	-	120-200 g	-	$>$150-200	6	120	0.050	[5]
PC	BS	SD rat	-	90-180 g	450-500	-	8	53	0.151	
PC	BC	SD rat	-	90-180 g	450-500	-	9	195	0.046	[6]
PC	SP_INT	SD rat	-	90-180 g	450-500	-	23	371	0.062	[6]
SCA	SCA	W rat	18-23 d	-	300-330	-	20	240	0.083	[7]
CCKBC	PC	SD rat	16-20 d	-	350	-			$<$0.100	[8]
SO	SLM	SD rat	18-22 d	-	300-350	-	1	20	0.050	[9]

Table S8: **Connection probabilities per m-type pair and other related parameters.**

[1] SD rat: Sprague Dawley rat, W rat: Wistar rat, LE rat: Long–Evans rat, G pig: Guinea pig.

References

[1] Deuchars J, Thomson AM. CA1 pyramid-pyramid connections in rat hippocampus in vitro: Dual intracellular recordings with biocytin filling;74(4):1009–1018. doi:10.1016/0306-4522(96)00251-5.

[2] Ali AB, Thomson AM. Facilitating pyramid to horizontal oriens-alveus interneurone inputs: dual intracellular recordings in slices of rat hippocampus;507(1):185–199. doi:10.1111/j.1469-7793.1998.185bu.x.

[3] Pawelzik H, Hughes DI, Thomson AM. Physiological and morphological diversity of immunocytochemically defined parvalbumin- and cholecystokinin-positive interneurones in CA1 of the adult rat hippocampus;443(4):346–367. doi:10.1002/cne.10118.

[4] Fuentealba P, Begum R, Capogna M, Jinno S, Márton LF, Csicsvari J, et al. Ivy Cells: A Population of Nitric-Oxide-Producing, Slow-Spiking GABAergic Neurons and Their Involvement in Hippocampal Network Activity;57(6):917–929. doi:10.1016/j.neuron.2008.01.034.

[5] Ali AB, Bannister AP, Thomson AM. IPSPs elicited in CA1 pyramidal cells by putative basket cells in slices of adult rat hippocampus: Basket cell IPSPs in CA1 pyramidal cells;11(5):1741–1753. doi:10.1046/j.1460-9568.1999.00592.x.

[6] Ali AB, Deuchars J, Pawelzik H, Thomson AM. CA1 pyramidal to basket and bistratified cell EPSPs: dual intracellular recordings in rat hippocampal slices. J Physiol. 1998;507 (Pt 1):201–217.

[7] Ali AB. CB1 modulation of temporally distinct synaptic facilitation among local circuit interneurons mediated by N-type calcium channels in CA1;105(3):1051–1062. doi:10.1152/jn.00831.2010.

[8] Neu A, Földy C, Soltesz I. Postsynaptic origin of CB1-dependent tonic inhibition of GABA release at cholecystokinin-positive basket cell to pyramidal cell synapses in the CA1 region of the rat hippocampus;578:233–247. doi:10.1113/jphysiol.2006.115691.

[9] Elfant D, Pál BZ, Emptage N, Capogna M. Specific inhibitory synapses shift the balance from feedforward to feedback inhibition of hippocampal CA1 pyramidal cells;27(1):104–113. doi:10.1111/j.1460-9568.2007.06001.x.
