# Supplementary material for: Community-based reconstruction and simulation of a full-scale model of the rat hippocampus CA1 region
Source: PLoS Biol. 2024 Nov 5;22(11):e3002861. doi: 10.1371/journal.pbio.3002861 (PMC11537418; doi:10.1371/journal.pbio.3002861)
Supplement: S9 Table — (PDF) [file pbio.3002861.s039.pdf]

From	To	Region	Species[1]	Age	Weight	mean	n. conns	STD	SEM	Reference
SP_BS	SP_PC	CA1	-	-	-	6	1	-	-	[1]
SP_PVBC	PV	CA1	SD rat	-	250-350 g	1.55	64	1.08	0.14	[2]
SP_PC	SO_OLM	CA1	W rat	14-21 d	-	2.83	6	1.94	0.79	[3]
SO_OLM	SP_PC	CA1	W rat	10-17 d	-	10	2	9.90	7	[4]
AA	GC	CA3	W rat	Young	-	8	1	-	-	[5]
AA	SP_PC	CA1	W rat	Young	-	5.89	9	-	-	[5]
SP_PC	SP_PC	CA1	SD rat	-	100 -180 g	1.17	6	0.41	0.17	[6]
SP_CCKBC	SP_PC	CA1	SD rat<br>Mouse	14-21 d<br>$>$ 21 d	-	8.3	14	0.8	0.21	[7]
SR_SCA	SP_PC	CA1	W rat	-	$>$ 120 g	5.33	3	1.15	0.67	[8]
SP_PVBC	SP_PC	CA1	SD rat<br>Mouse	14-21 d<br>$>$ 21 d	-	11	15	0.6	0.15	[7]
SR_SCA	SR_SCA	CA1	W rat	18-21 d	-	3.5	9	1.5	0.5	[9]

Table S9: **Number of synapses per connection.**

[1] SD rat: Sprague Dawley rat, W rat: Wistar rat, LE rat: Long–Evans rat, G pig: Guinea pig.

References

[1] Buhl EH, Halasy K, Somogyi P. Diverse sources of hippocampal unitary inhibitory postsynaptic potentials and the number of synaptic release sites;368(6474):823–828. doi:10.1038/368823a0.

[2] Sik A, Penttonen M, Ylinen A, Buzsáki G. Hippocampal CA1 interneurons: an in vivo intracellular labeling study;15(10):6651–6665. doi:10.1523/JNEUROSCI.15-10-06651.1995.

[3] Biro AA. Quantal Size Is Independent of the Release Probability at Hippocampal Excitatory Synapses;25(1):223–232. doi:10.1523/JNEUROSCI.3688-04.2005.

[4] Maccaferri G, David J, Roberts B, Szucs P, Cottingham CA, Somogyi P. Cell surface domain specific postsynaptic currents evoked by identified GABAergic neurones in rat hippocampus *in vitro*;524(1):91–116. doi:10.1111/j.1469-7793.2000.t01-3-00091.x.

[5] Buhl EH, Han ZS, Lorinczi Z, Stezhka VV, Karnup SV, Somogyi P. Physiological properties of anatomically identified axo-axonic cells in the rat hippocampus;71(4):1289–1307. doi:10.1152/jn.1994.71.4.1289.

[6] Deuchars J, Thomson AM. CA1 pyramid-pyramid connections in rat hippocampus in vitro: Dual intracellular recordings with biocytin filling;74(4):1009–1018. doi:10.1016/0306-4522(96)00251-5.

[7] Földy C, Lee SH, Morgan RJ, Soltesz I. Regulation of fast-spiking basket cell synapses by the chloride channel ClC-2;13(9):1047–1049. doi:10.1038/nn.2609.

[8] Vida I, Halasy K, Szinyei C, Somogyi P, Buhl EH. Unitary IPSPs evoked by interneurons at the stratum radiatum-stratum lacunosum-moleculare border in the CA1 area of the rat hippocampus *in vitro*;506(3):755–773. doi:10.1111/j.1469-7793.1998.755bv.x.

[9] Ali AB. CB1 modulation of temporally distinct synaptic facilitation among local circuit interneurons mediated by N-type calcium channels in CA1;105(3):1051–1062. doi:10.1152/jn.00831.2010.
