# Supplementary material for: Community-based reconstruction and simulation of a full-scale model of the rat hippocampus CA1 region
Source: PLoS Biol. 2024 Nov 5;22(11):e3002861. doi: 10.1371/journal.pbio.3002861 (PMC11537418; doi:10.1371/journal.pbio.3002861)
Supplement: S10 Table — (PDF) [file pbio.3002861.s040.pdf]

M-type	Region	Species[1]	Age	Weight	mean	n. cells	std	SEM	Reference
SP_PVBC	CA1	SD rat	-	250-350 g	10436	4	1393.26	696.63	[1]
SP_BC	CA1	W rat	7-8 w	-	10828	1	0.00	0.00	[2]
SR_SCA	CA1	W rat	-	>120 g	5998	1	0.00	0.00	[3]
SR_CCKBC	CA1	W rat	-	>120 g	7964	1	0.00	0.00	[3]
SP_BS	CA1	SD / W rat	7-8 w	250-350 g	12676	2	5549.37	3924.00	[1]; [2]
SO_OLM	CA1	SD rat	-	250-350 g	16847	1	0.00	0.00	[1]
SLM_PPA	CA1	W rat	-	>120 g	8015	1	0.00	0.00	[3]
SO_Tri	CA1	SD rat	-	250-350 g	15767	1	0.00	0.00	[1]

Table S10: **Experimentally available data for divergence of synapses per m-type.**

[1] SD rat: Sprague Dawley rat, W rat: Wistar rat, LE rat: Long–Evans rat, G pig: Guinea pig.

References

[1] Sik A, Penttonen M, Ylinen A, Buzsáki G. Hippocampal CA1 interneurons: an in vivo intracellular labeling study;15(10):6651–6665. doi:10.1523/JNEUROSCI.15-10-06651.1995.

[2] Halasy K, Buhl EH, Lbrinczi Z, Tamas G, Somogyi P. Synaptic Target Selectivity and Input of GABAergic Basket and Bistratified Interneurons in the CAI Area of the Rat Hippocampus; p. 24.

[3] Vida I, Halasy K, Szinyei C, Somogyi P, Buhl EH. Unitary IPSPs evoked by interneurons at the stratum radiatum-stratum lacunosum-moleculare border in the CA1 area of the rat hippocampus *in vitro*;506(3):755–773. doi:10.1111/j.1469-7793.1998.755bv.x.
