# Supplementary material for: Community-based reconstruction and simulation of a full-scale model of the rat hippocampus CA1 region
Source: PLoS Biol. 2024 Nov 5;22(11):e3002861. doi: 10.1371/journal.pbio.3002861 (PMC11537418; doi:10.1371/journal.pbio.3002861)
Supplement: S11 Table — (PDF) [file pbio.3002861.s041.pdf]

M-type	Species[1]	Age	Weight	PC	INT	n	Reference
PC	SD rat	-	250-350 g	42.151	57.849	130	[1]
OLM	W rat	2 m	-	89.157	10.843	34	[2]
Tri	W rat	-	300-400 g	40	60	52	[3]
AA	-	-	-	100	0	-	AA.VV.

Table S11: **Available data on the divergence of synapses for different m-types to excitatory and inhibitory groups within CA1.**

[1] SD rat: Sprague Dawley rat, W rat: Wistar rat, LE rat: Long–Evans rat, G pig: Guinea pig.

References

[1] Takács VT, Klausberger T, Somogyi P, Freund TF, Gulyás AI. Extrinsic and local glutamatergic inputs of the rat hippocampal CA1 area differentially innervate pyramidal cells and interneurons;22(6):1379–1391. doi:10.1002/hipo.20974.

[2] Katona I, Sperlágh B, Sık A, Käfalvi A, Vizi ES, Mackie K, et al. Presynaptically Located CB1 Cannabinoid Receptors Regulate GABA Release from Axon Terminals of Specific Hippocampal Interneurons. The Journal of Neuroscience. 1999;19(11):4544–4558. doi:10.1523/jneurosci.19-11-04544.1999.

[3] Ferraguti F, Klausberger T, Cobden P, Baude A, Roberts JDB, Szucs P, et al. Metabotropic Glutamate Receptor 8-Expressing Nerve Terminals Target Subsets of GABAergic Neurons in the Hippocampus. The Journal of Neuroscience. 2005;25(45):10520–10536. doi:10.1523/jneurosci.2547-05.2005.
