# Supplementary material for: Community-based reconstruction and simulation of a full-scale model of the rat hippocampus CA1 region
Source: PLoS Biol. 2024 Nov 5;22(11):e3002861. doi: 10.1371/journal.pbio.3002861 (PMC11537418; doi:10.1371/journal.pbio.3002861)
Supplement: S12 Table — Data for SO_Tri comes from the Sprague Dawley rat data set (see section Experimental procedures). (PDF) [file pbio.3002861.s042.pdf]

	SO	SP	SR	SLM	N.neurons	N. boutons
SLM_PPA	0.000	0.000	37.700	62.300	1	891
SO_BS	48.100	12.400	39.500	0.000	1	2027
SO_Tri	58.120	18.110	23.770	0.000	1	4627
SP_BS	47.600	9.850	42.550	0.000	2	8070
SP_CCKBC	29.400	62.850	7.750	0.000	2	3036
SR_SCA	0.000	0.200	97.400	2.400	1	4137
SR_CCKBC	9.100	57.700	31.750	1.450	2	1878
SR_Tri	27.000	23.000	50.000	0.000	1	649

Table S12: **Laminar distribution.** Data for SO_Tri comes from the Sprague Dawley rat dataset (see Section Experimental procedures), while the rest from [1].

References

[1] Pawelzik H, Hughes DI, Thomson AM. Physiological and morphological diversity of immunocytochemically defined parvalbumin- and cholecystokinin-positive interneurones in CA1 of the adult rat hippocampus;443(4):346–367. doi:10.1002/cne.10118.
