# Supplementary material for: Community-based reconstruction and simulation of a full-scale model of the rat hippocampus CA1 region
Source: PLoS Biol. 2024 Nov 5;22(11):e3002861. doi: 10.1371/journal.pbio.3002861 (PMC11537418; doi:10.1371/journal.pbio.3002861)
Supplement: S13 Table — (PDF) [file pbio.3002861.s043.pdf]

Rule	From	To	Rule type[1]	U (mean ± STD)	D (ms) (mean ± STD)	F (ms) (mean ± STD)	N_RRP	Hill scaling	CV Validation pathway used
1	SP_PC	SP_PC	E2	0.65±0.1	671±17	17±5	2	2.79	-
2	SP_PC	SO_OLM	E1	0.09±0.12	138±211	670±830	1	2.79	-
3	SP_PC	SO_Tri<br>SO_BS<br>SO_BP	E1	0.09±0.12	138±211	670±830	1	1.94	-
4	SP_PC	SP_AA	E2	0.23±0.09	410±190	10±11	1	1.09	-
5	SP_PC	SP_BS	E2	0.23±0.09	410±190	10±11	1	1.94	-
6	SP_PC	SP_CCKBC	E2	0.23±0.09	410±190	10±11	1	1.09	-
7	SP_PC	SP_Ivy	E2	0.5±0.02	671±17	17±5	1	1.94	-
8	SP_PC	SP_PVBC	E2	0.23±0.09	410±190	10±11	1	1.09	-
9	SP_PC	SR_SCA<br>SLM_PPA	E2	0.23±0.09	410±190	10±11	1	1.94	-
10	INH	INH	I2	0.26±0.05	930±360	1.6±0.6	1	1.94	-
11	SP_AA	SP_PC	I2	0.1±0.01	1278±760	10±6.7	1	1.94	SP_AA→SP_PC
12	SP_BS	SP_PC	I2	0.13±0.03	1122±156	9.3±0.7	1	1.94	-
13	SP_PVBC	SP_PC	I2	0.16±0.02	965±185	8.6±4.3	9	1.94	SP_PVBC→SP_PC
14	SO_OLM<br>SO_BS<br>SO_BP	SP_PC	I2	0.3±0.08	1250±520	2±14	1	1.94	-
15	SO_Tri	SP_PC	I2	0.3±0.08	1250±520	2±14	1	1.94	-
16	SLM_PPA	SP_PC	I3	0.16±0.01	168±15	13±0.5	1	1.94	-
17	SP_CCKBC	SP_PC	I3	0.16±0.04	153±120	12±3.5	1	1.94	SP_CCKBC→SP_PC
18	SR_SCA	SP_PC	I3	0.15±0.03	185±32	14±5.8	1	1.94	SR_SCA→SP_PC
19	SP_Ivy	SP_PC	I3	0.32±0.14	144±80	62±31	1	1.94	-

20	SP_PVBC	SP_AA	I2	0.24±0.15	1730±530	3.5±1.5	1	1.94	SP_PVBC→SP_AA
	SP_CCKBC	SP_CCKBC							
21	SR_SCA	SR_SCA	I1	0.11±0.03	115±100	1542±700	3	1.94	SP_CCKBC→SP_CCKBC
	SLM_PPA	SLM_PPA							
22	SP_PVBC	SP_PVBC	I2	0.26±0.05	930±360	1.6±0.6	9	1.94	SP_PVBC→SP_PVBC

Table S13: **Presynaptic dynamics parameters.**

[1]Rule types: E1: excitatory facilitating, E2: excitatory depressing, I1: inhibitory facilitating, I2: inhibitory depressing, I3: inhibitory pseudo linear.
