# Supplementary material for: Community-based reconstruction and simulation of a full-scale model of the rat hippocampus CA1 region
Source: PLoS Biol. 2024 Nov 5;22(11):e3002861. doi: 10.1371/journal.pbio.3002861 (PMC11537418; doi:10.1371/journal.pbio.3002861)
Supplement: S14 Table — (PDF) [file pbio.3002861.s044.pdf]

Rule	From	To	Rule type[1]	gsyn (nS) (mean ± STD)	τ_{decay} fast (ms) (mean ± STD)	NMDA/AMPA ratio (mean ± STD)	τ_{decay} NMDA (ms) (mean ± STD)	PSP validation pathway used
1	SP_PC	SP_PC	E2	0.65±0.1	3±0.2	1.22	148.5	SP_PC→SP_PC
2	SP_PC	SO_OLM	E1	1.0±0.05	1.7±0.14	0.28	148.5	SP_PC→SO_OLM
3	SP_PC	SO_Tri<br>SO BS<br>SO BP	E1	1.0±0.05	1.7±0.14	0.28	148.5	SP_PC→SO_OLM
4	SP_PC	SP_AA	E2	2.93±0.7	4.12±0.5	0.28	148.5	MEAN RULES 5-8
5	SP_PC	SP_BS	E2	1.88±0.1	4.12±0.5	0.28	148.5	SP_PC→SP_BS
6	SP_PC	SP_CCKBC	E2	3.63±0.4	4.12±0.5	0.86	298.75	SP_PC→SP_CCKBC
7	SP_PC	SP_Ivy	E2	3.64±0.4	4.12±0.5	0.28	148.5	SP_PC→SP_Ivy
8	SP_PC	SP_PVBC	E2	2.56±0.05	4.12±0.4	0.28	148.5	SP_PC→SP_PVBC
9	SP_PC	SR_SCA<br>SLM_PPA	E2	2.93±0.7	4.12±0.5	0.86	298.75	MEAN RULES 5-8
10	INH	INH	I2	3.74±0.3	4±0.8	-	-	SP_PVBC→SP_PVBC
11	SP_AA	SP_PC	I2	2.01±0.1	11.2±0.9	-	-	SP_AA→SP_PC
12	SP_BS	SP_PC	I2	1.92±0.1	16.1±1.1	-	-	SP_BS→SP_PC
13	SP_PVBC	SP_PC	I2	1.87±0.2	5.94±0.47	-	-	SP_PVBC→SP_PC
14	SO_OLM<br>SO BS<br>SO BP	SP_PC	I2	1.61±0.3	8.3±2.2	-	-	SO_Tri→SP_PC
15	SO_Tri	SP_PC	I2	1.61±0.3	7.75±0.9	-	-	SO_Tri→SP_PC
16	SLM_PPA	SP_PC	I3	2.2±0.15	8.8±0.25	-	-	MEAN RULES 17-18
17	SP_CCKBC	SP_PC	I3	1.7±0.3	9.35±1.0	-	-	SP_CCKBC→SP_PC
18	SR_SCA	SP_PC	I3	2.69±0.3	8.3±0.44	-	-	SR_SCA→SP_PC

19	SP_Ivy	SP_PC	I3	0.65±0.05	16±2.5	-	-	SP_Ivy→SP_PC
20	SP_PVBC<br>SP_CCKBC	SP_AA<br>SP_CCKBC	I2	3.74±0.3	2.67±0.13	-	-	SP_PVBC→SP_PVBC
21	SR_SCA<br>SLM_PPA	SR_SCA<br>SLM_PPA	I1	3.74±0.3	4.5±0.55	-	-	SP_PVBC→SP_PVBC
22	SP_PVBC	SP_PVBC	I2	3.74±0.3	2.67±0.13	-	-	SP_PVBC→SP_PVBC

Table S14: **Postsynaptic dynamics parameters.**

[1]Rule types: E1: excitatory facilitating, E2: excitatory depressing, I1: inhibitory facilitating, I2: inhibitory depressing, I3: inhibitory pseudo linear.
