# Supplementary material for: Community-based reconstruction and simulation of a full-scale model of the rat hippocampus CA1 region
Source: PLoS Biol. 2024 Nov 5;22(11):e3002861. doi: 10.1371/journal.pbio.3002861 (PMC11537418; doi:10.1371/journal.pbio.3002861)
Supplement: S15 Table — N.: number of animals, n.: number of synapses. (PDF) [file pbio.3002861.s045.pdf]

From	To	Experimental Feature	Min	Max	Mean	SD	Species[1]	Weight	Region	N.	n.	Reference
SC	EXC	Number of afferent synapses	13059	28697	20878	-	W rat	300 g	CA1	7	-	[1]
SC	INH	Number of afferent synapses	7952	17476	12714	-	W rat	>110 g	CA1	4	70	[1]
SC	PV+ INTs	Number of synapses per connection	1	3	1.19	0.48	SD rat	200-300 g	CA3	-	274	[2]
SC	All	Number of efferent synapses	15295	27440	21368	-	SD rat	200-300 g	CA1	6	-	[1]

Table S15: **Schaffer collaterals anatomy experimental data.** N.: number of animals, n.: number of synapses.

[1] SD rat: Sprague Dawley rat, W rat: Wistar rat, LE rat: Long–Evans rat, G pig: Guinea pig.

References

[1] Bezaire MJ, Soltesz I. Quantitative assessment of CA1 local circuits: Knowledge base for interneuron-pyramidal cell connectivity: Quantitative Assessment Of Ca1 Local Circuits;23(9):751–785. doi:10.1002/hipo.22141.

[2] Sik A, Tamamaki N, Freund TF. Complete Axon Arborization of a Single CA3 Pyramidal Cell in the Rat Hippocampus, and its Relationship With Postsynaptic Parvalbumin-containing Interneurons;5(12):1719–1728. doi:10.1111/j.1460-9568.1993.tb00239.x.
