# Supplementary material for: Community-based reconstruction and simulation of a full-scale model of the rat hippocampus CA1 region
Source: PLoS Biol. 2024 Nov 5;22(11):e3002861. doi: 10.1371/journal.pbio.3002861 (PMC11537418; doi:10.1371/journal.pbio.3002861)
Supplement: S16 Table — N.: number of animals. (PDF) [file pbio.3002861.s046.pdf]

| From | To | Average synapse number | Percentage of afferent synapses | Species[1] | Weight | Region | N. | Reference |
|---|---|---|---|---|---|---|---|---|
| SC | SLM | 55 | 0.3% | SD rat | 200-300 g | CA1 | 6 | [1] |
| SC | SR | 14515 | 67.9% | SD rat | 200-300 g | CA1 | 6 | [1] |
| SC | SP | 1507 | 7.1% | SD rat | 200-300 g | CA1 | 6 | [1] |
| SC | SO | 5291 | 24.7% | SD rat | 200-300 g | CA1 | 6 | [1] |

Table S16: **Schaffer collaterals layer profile.** N.: number of animals. Averages are taken from Table 20 of [1]

[1] SD rat: Sprague Dawley rat, W rat: Wistar rat, LE rat: Long–Evans rat, G pig: Guinea pig.

## References

[1] Bezaire MJ, Soltesz I. Quantitative assessment of CA1 local circuits: Knowledge base for interneuron-pyramidal cell connectivity: Quantitative Assessment Of Ca1 Local Circuits;23(9):751–785. doi:10.1002/hipo.22141.
