# Supplementary material for: Community-based reconstruction and simulation of a full-scale model of the rat hippocampus CA1 region
Source: PLoS Biol. 2024 Nov 5;22(11):e3002861. doi: 10.1371/journal.pbio.3002861 (PMC11537418; doi:10.1371/journal.pbio.3002861)
Supplement: S17 Table — (PDF) [file pbio.3002861.s047.pdf]

SC$\rightarrow$Exc	Experimental Feature	Value (Mean)	SD	SEM	UoM	Species[1]	Age	Weight	R.	n.	Reference
PSP Magnitude	PSPs amplitudes	0.14	0.106	0.01	mV	G Pig	-	600-900 g	CA1	72	[1]
	PSPs CV	0.76	-	-	-	G Pig	-	600-900 g	CA1	72	[1]
PSP Kinetics	PSPs rise time	3.9	1.8	0.21	ms	G Pig	-	600-900 g	CA1	72	[1]
	PSPs half-width	19.5	8	0.94	ms	G Pig	-	600-900 g	CA1	72	[1]
	PSP tau decay	22.6	11	1.3	ms	G Pig	-	600-900 g	CA1	72	[1]
NMDA Kinetics	NMDA/AMPA ratio	1.23	0.03	0.01	-	Mouse	17-23 d	-	CA1	12	[2]
	NMDA tau rise	2.93	-	-	ms	SD rat	6-12 w	-	CA1	52	[3]
	NMDA tau decay	148.5	-	-	ms	SD rat	6-12 w	-	CA1	52	[3]
Short term plasticity	U	0.14	0.08	0.03	-	W rat	14-28 d	-	CA1	8	[4]
	D	186	71	25.1	ms	W rat	14-28 d	-	CA1	8	[4]
	F	129	68	24.04	ms	W rat	14-28 d	-	CA1	8	[4]

Table S17: **Schaffer collaterals physiology experimental data for SC$\rightarrow$Exc synapses.** UoM: Units of Measurement, R.: region, n.: number of cells.

[1] SD rat: Sprague Dawley rat, W rat: Wistar rat, LE rat: Long–Evans rat, G pig: Guinea pig.

References

[1] Sayer RJ, Friedlander MJ, Redman SJ. The time course and amplitude of EPSPs evoked at synapses between pairs of CA3/CA1 neurons in the hippocampal slice;10(3):826–836.

[2] Le Roux N, Cabezas C, Böhm UL, Poncer JC. Input-specific learning rules at excitatory synapses onto hippocampal parvalbumin-expressing interneurons;591(7):1809–1822. doi:10.1113/jphysiol.2012.245852.

[3] Andrásfalvy BK, Magee JC. Distance-Dependent Increase in AMPA Receptor Number in the Dendrites of Adult Hippocampal CA1 Pyramidal Neurons;21(23):9151–9159. doi:10.1523/JNEUROSCI.21-23-09151.2001.

[4] Wierenga CJ, Wadman WJ. Excitatory Inputs to CA1 Interneurons Show Selective Synaptic Dynamics;90(2):811–821. doi:10.1152/jn.00865.2002.
