# Supplementary material for: Community-based reconstruction and simulation of a full-scale model of the rat hippocampus CA1 region
Source: PLoS Biol. 2024 Nov 5;22(11):e3002861. doi: 10.1371/journal.pbio.3002861 (PMC11537418; doi:10.1371/journal.pbio.3002861)
Supplement: S18 Table — (PDF) [file pbio.3002861.s048.pdf]

| SC→Inh | Experimental Feature | Value (Mean) | SD | SEM | UoM | Species[1] | Age | R. | n. | Reference |
|---|---|---|---|---|---|---|---|---|---|---|
| PSC | PSCs ratios SC-INH/SC-PC | 1.09 | 1.14 | 0.29 | - | W rat | 4-6 w | CA1 | 16 | [1] |
| Magnitude | PSCs ratios SC-INH/SC-PC | 8.15 | 6 | 1.5 | - | W rat | 4-6 w | CA1 | 16 | [1] |
| PSP Kinetics | EPSP-IPSP latency | 1.9 | 0.6 | 0.2 | ms | W rat | 10 d | CA1 | 9 | [2] |
| NMDA | NMDA/AMPA ratio | 0.16 | 0.07 | 0.02 | - | Mouse | 17-23 d | CA1 | 12 | [3] |
| Kinetics | NMDA tau rise | 2.93 | - | - | ms | SD rat | 6-12 w | CA1 | 52 | [4] |
| | NMDA tau decay | 154.6 | 81.9 | 25.9 | ms | Mouse | 14-24 d | CA1 | 10 | [5] |
| Short term | U | 0.11 | 0.04 | 0.01 | - | W rat | 14-28 d | CA1 | 20 | [6] |
| plasticity | D | 307 | 233 | 52.1 | ms | W rat | 14-28 d | CA1 | 20 | [6] |
| | F | 195 | 134 | 29.96 | ms | W rat | 14-28 d | CA1 | 20 | [6] |

Table S18: **Schaffer collaterals physiology experimental data for SC→Inh synapses.** UoM: Units of Measurement, R.: region, n.: number of cells.

[1] SD rat: Sprague Dawley rat, W rat: Wistar rat, LE rat: Long–Evans rat, G pig: Guinea pig.

## References


[1] Glickfeld LL, Scanziani M. Distinct timing in the activity of cannabinoid-sensitive and cannabinoid-insensitive basket cells;9(6):807–815. doi:10.1038/nn1688.

[2] Pouille F, Scanziani M. Enforcement of temporal fidelity in pyramidal cells by somatic feed-forward inhibition;293(5532):1159–1163. doi:10.1126/science.1060342.

[3] Le Roux N, Cabezas C, Böhm UL, Poncer JC. Input-specific learning rules at excitatory synapses onto hippocampal parvalbumin-expressing interneurons;591(7):1809–1822. doi:10.1113/jphysiol.2012.245852.

[4] Andrásfalvy BK, Magee JC. Distance-Dependent Increase in AMPA Receptor Number in the Dendrites of Adult Hippocampal CA1 Pyramidal Neurons;21(23):9151–9159. doi:10.1523/JNEUROSCI.21-23-09151.2001.

[5] Cornford JH, Mercier MS, Leite M, Magloire V, Häusser M, Kullmann DM. Dendritic NMDA receptors in parvalbumin neurons enable strong and stable neuronal assemblies;8:e49872. doi:10.7554/eLife.49872.

[6] Wierenga CJ, Wadman WJ. Excitatory Inputs to CA1 Interneurons Show Selective Synaptic Dynamics;90(2):811–821. doi:10.1152/jn.00865.2002.
