# Supplementary material for: Community-based reconstruction and simulation of a full-scale model of the rat hippocampus CA1 region
Source: PLoS Biol. 2024 Nov 5;22(11):e3002861. doi: 10.1371/journal.pbio.3002861 (PMC11537418; doi:10.1371/journal.pbio.3002861)
Supplement: S19 Table — (PDF) [file pbio.3002861.s049.pdf]

Resting Membrane Potential (RMP)

Neuron Type	Dose (μM)	Drug[1]	Application	Region	Layer	Species[2]	Age Weight	Vm ctr (mV)	Vm ACh (mV)	ΔVm (mV)	Current (nA)	N. cells	Reference
PC	10	CCh	focal	CA1	SP	Mouse	Adult	-79	-75.4	3.6	0.12	13	[1]
FSBC (PVBC)	5	CCh	bath	CA3	SP	Mouse	15-23 d	-65	-58.9	6.1	0.17	8	[2]
AAC	5	CCh	bath	CA3	SP	Mouse	15-23 d	-65	-61.4	3.6	0.07	11	[2]
RSBC	5	CCh	bath	CA3	SP	Mouse	15-23 d	-65	-58.7	6.3	0.13	7	[2]
INTs	10	CCh ACh	focal	CA1	all	Mouse	18-25 d	-70	-55.5	14.5	0.37	102	[3]
PC	3	CCh	bath	CA1	SP	SD rat	Adult	-66	-61	5	0.16	4	[4]
PC	10	CCh	bath	CA1	SP	Mouse	10-40 d	-61.3	-56	5.3	0.17	12	[5]
PC	1	CCh	bath	CA1	SP	W rat	13-15 d	-75	-72.4	2.6	0.09	8	[6]
PC	50	CCh	TBC	CA1	SP	SD rat	11-28 d	-72	-65	7	0.23	9	[7]
PC	100	CCh	bath	SUB	all	SD rat	200 300 g	-62.2	-53.2	9	0.3	9	[8]

Firing Rate (FR)

Neuron Type	Dose (μM)	Drug[7]	Application	Region	Layer	Species[1]	Age Weight	FR ctr (Hz)	FR ACh (Hz)	ΔFR (Hz)	Current (nA)	N. cells	Reference
OLM	10	Musc ACh	bath	CA1	SO	Mouse	14-21 d	20	25	5	0.02	43	[9]
Other ADP	10	Musc ACh	bath	CA1	SO	Mouse	14-21 d	20	37	17	0.12	43	[9]
PC	3	CCh	bath	CA1	SP	SD rat	Adult	6	9	3	0.06	4	[4]

CCKSCA	10	Musc	bath		CA1	SR	Mouse	15-20 d	12.7	32	19.3	0.07	21	[10]

Table S19: **Curated dataset on neuronal excitability changes caused by cholinergic modulation.**

[1]ACh: Acetylcholine, CCh: Carbachol, Musc: Muscarine

[2]SD rat: Sprague Dawley rat, W rat: Wistar rat, LE rat: Long–Evans rat, G pig: Guinea pig.

References

[1] Dasari S, Gulledge AT. M1 and M4 Receptors Modulate Hippocampal Pyramidal Neurons;105(2):779–792. doi:10.1152/jn.00686.2010.

[2] Szabó GG, Holderith N, Gulyás AI, Freund TF, Hájos N. Distinct synaptic properties of perisomatic inhibitory cell types and their different modulation by cholinergic receptor activation in the CA3 region of the mouse hippocampus: Synaptic properties of perisomatic interneurons;31(12):2234–2246. doi:10.1111/j.1460-9568.2010.07292.x.

[3] McQuiston AR, Madison DV. Muscarinic Receptor Activity Has Multiple Effects on the Resting Membrane Potentials of CA1 Hippocampal Interneurons;19(14):5693–5702. doi:10.1523/JNEUROSCI.19-14-05693.1999.

[4] Sheridan RD, Sutor B. Presynaptic M1 muscarinic cholinoceptors mediate inhibition of excitatory synaptic transmission in the hippocampus in vitro;108(3):273–278. doi:10.1016/0304-3940(90)90653-Q.

[5] Palacios-Filardo J, Udakis M, Brown GA, Tehan BG, Congreve MS, Nathan PJ, et al. Acetylcholine prioritises direct synaptic inputs from entorhinal cortex to CA1 by differential modulation of feedforward inhibitory circuits;12(1):5475. doi:10.1038/s41467-021-25280-5.

[6] Buchanan KA, Petrovic MM, Chamberlain SEL, Marrion NV, Mellor JR. Facilitation of Long-Term Potentiation by Muscarinic M1 Receptors Is Mediated by Inhibition of SK Channels;68(5):948–963. doi:10.1016/j.neuron.2010.11.018.

[7] Williams JH, Kauer JA. Properties of carbachol-induced oscillatory activity in rat hippocampus;78(5):2631–2640. doi:10.1152/jn.1997.78.5.2631.

[8] Kawasaki H, Avoli M. Excitatory effects induced by carbachol on bursting neurons of the rat subiculum;219(1):1–4. doi:10.1016/S0304-3940(96)13175-X.

[9] Lawrence JJ, Statland JM, Grinspan ZM, McBain CJ. Cell type-specific dependence of muscarinic signalling in mouse hippocampal stratum oriens interneurones: mAChR modulation of str. oriens interneurones;570(3):595–610. doi:10.1113/jphysiol.2005.100875.

[10] Cea-del Rio CA, Lawrence JJ, Erdelyi F, Szabó G, McBain CJ. Cholinergic modulation amplifies the intrinsic oscillatory properties of CA1 hippocampal cholecystokinin-positive interneurons: Cholinergic modulation and CA1 cholecystokinin-positive interneurons;589(3):609–627. doi:10.1113/jphysiol.2010.199422.
