# Supplementary material for: Community-based reconstruction and simulation of a full-scale model of the rat hippocampus CA1 region
Source: PLoS Biol. 2024 Nov 5;22(11):e3002861. doi: 10.1371/journal.pbio.3002861 (PMC11537418; doi:10.1371/journal.pbio.3002861)
Supplement: S20 Table — (PDF) [file pbio.3002861.s050.pdf]

From	To	Dose (μM)	Drug[1]	Application	Region	Layer	Species[2]	Age Weight	PSP PSC	Ratio ACh/ctr	N. conn	Reference
SC	PC	10	CCh	focal	CA1	SP	Mouse	Adult	EPSP	38%	9	[1]
PC/SC	PC	0.01	CCh	bath	CA1	SLM SR	SD rat	5-10 w	EPSP	100%	5	[2]
PC/SC	PC	0.1	CCh	bath	CA1	SLM SR	SD rat	5-10 w	EPSP	96%	5	[2]
PC/SC	PC	1	CCh	bath	CA1	SLM SR	SD rat	5-10 w	EPSP	81%	5	[2]
PC/SC	PC	10	CCh	bath	CA1	SLM SR	SD rat	5-10 w	EPSP	74%	5	[2]
PC/SC	PC	100	CCh	bath	CA1	SLM SR	SD rat	5-10 w	EPSP	47%	13	[2]
PC/SC	PC	500	CCh	bath	CA1	SLM SR	SD rat	5-10 w	EPSP	0%	5	[2]
PC/SC	PC	0.01	CCh	bath	CA1	SLM SR	SD rat	5-10 w	EPSP	100%	5	[2]
PC/SC	PC	0.1	CCh	bath	CA1	SLM SR	SD rat	5-10 w	EPSP	98%	5	[2]
PC/SC	PC	1	CCh	bath	CA1	SLM SR	SD rat	5-10 w	EPSP	67%	5	[2]
PC/SC	PC	10	CCh	bath	CA1	SLM SR	SD rat	5-10 w	EPSP	43%	5	[2]
PC/SC	PC	100	CCh	bath	CA1	SLM SR	SD rat	5-10 w	EPSP	13%	13	[2]

PC/SC	PC	500	CCh	bath	CA1	SLM SR	SD rat	5-10 w	EPSP	0%	5	[2]
MF	PC	1	Musc	bath	CA3	-	Rat	100 200 g	EPSP	100%	19	[3]
MF	PC	1	Musc	bath	CA3	-	Rat	100 200 g	EPSC	89%	14	[3]
MF	PC	10	Musc	bath	CA3	-	Rat	100 200 g	EPSP	77%	7	[3]
MF	PC	10	Musc	bath	CA3	-	Rat	100 200 g	EPSC	66%	7	[3]
FSBC	PC	5	CCh	bath	CA3	SP	Mouse	15-23 d	IPSC	30%	16	[4]
AAC	PC	5	CCh	bath	CA3	SP	Mouse	15-23 d	IPSC	27%	16	[4]
RSBC	PC	5	CCh	bath	CA3	SP	Mouse	15-23 d	IPSC	6%	13	[4]
SC	PC	5	CCh	bath	CA1	SP	W rat	14-18 d	EPSC	32%	11	[5]
SCC	PC	0.1	CCh	bath	CA1	SP	SD rat	Adult	EPSP	96%	12	[6]
SCC	PC	0.3	CCh	bath	CA1	SP	SD rat	Adult	EPSP	83%	12	[6]
SCC	PC	1	CCh	bath	CA1	SP	SD rat	Adult	EPSP	60%	12	[6]
SCC	PC	3	CCh	bath	CA1	SP	SD rat	Adult	EPSP	30%	12	[6]
SCC	PC	10	CCh	bath	CA1	SP	SD rat	Adult	EPSP	6%	12	[6]

Table S20: **Curated dataset on synaptic transmission changes caused by cholinergic modulation.**

[1] ACh: Acetylcholine, CCh: Carbachol, Musc: Muscarine

[2] SD rat: Sprague Dawley rat, W rat: Wistar rat, LE rat: Long–Evans rat, G pig: Guinea pig.

References

[1] Dasari S, Gulledge AT. M1 and M4 Receptors Modulate Hippocampal Pyramidal Neurons;105(2):779–792. doi:10.1152/jn.00686.2010.

[2] Hasselmo M, Schnell E. Laminar selectivity of the cholinergic suppression of synaptic transmission in rat hippocampal region CA1: computational modeling and brain slice physiology;14(6):3898–3914. doi:10.1523/JNEUROSCI.14-06-03898.1994.

[3] Williams S, Johnston D. Muscarinic depression of synaptic transmission at the hippocampal mossy fiber synapse;64(4):1089–1097. doi:10.1152/jn.1990.64.4.1089.

[4] Szabó GG, Holderith N, Gulyás AI, Freund TF, Hájos N. Distinct synaptic properties of perisomatic inhibitory cell types and their different modulation by cholinergic receptor activation in the CA3 region of the mouse hippocampus: Synaptic properties of perisomatic interneurons;31(12):2234–2246. doi:10.1111/j.1460-9568.2010.07292.x.

[5] Sevilla DF, Cabezas C, Prada ANO, Sánchez-Jiménez A, Buño W. Selective muscarinic regulation of functional glutamatergic Schaffer collateral synapses in rat CA1 pyramidal neurons;545(1):51–63. doi:10.1113/jphysiol.2002.029165.

[6] Sheridan RD, Sutor B. Presynaptic M1 muscarinic cholinoceptors mediate inhibition of excitatory synaptic transmission in the hippocampus in vitro;108(3):273–278. doi:10.1016/0304-3940(90)90653-Q.
