# Supplementary material for: Community-based reconstruction and simulation of a full-scale model of the rat hippocampus CA1 region
Source: PLoS Biol. 2024 Nov 5;22(11):e3002861. doi: 10.1371/journal.pbio.3002861 (PMC11537418; doi:10.1371/journal.pbio.3002861)
Supplement: S21 Table — (PDF) [file pbio.3002861.s051.pdf]

Species[1]	Age Weight	Dose (μM)	Drug[2 3]	Region	Layer	Slice Thickness (μm)	ACSF (mM) Ca	Mg	K	Measurement	Effects	N.	Reference
G pig	2-3 m	50	CCh	CA1 and CA3	SP	500	2	1.6	5	Intracellular recording of PC	CCh-induced rhythmic bursts were recorded in both CA3 and CA1 PC of intact hippocampal slices. Where the CA3 and CA1 regions had been separated by a razor blade cut, rhythmic bursts were still observed in CA3 but not in CA1 neurons.	5	[1]
SD rat	11-28 d	50	CCh	CA1 and CA3	SR	400	2.5	1.3	2.5	Extracellular recording in SR	After a cut between CA3 and CA1, CCh induced robust oscillations in CA3, but not in CA1.	4	[2]
W rat	15-25 d	20	CCh	CA1 and CA3	SR	450	2	2	3	Extracellular recording in SR	CCh-induced 40-Hz oscillations are generated within the CA3 area and propagate to CA1 in intact slice. After the cut between CA3 and CA1, oscillations in CA3 persisted, whereas activity was observed in neither CA1 nor dentate gyrus.	4	[3]
SD rat	10-30 d	4-13	CCh	CA1 and CA3	SP	400	2	2	5	Extracellular recording in SP	Low concentrations of CCh (4-13 μM) produced a regular pattern of synchronous discharges in the delta range (0.5-2 Hz). This rhythmic pattern originated in CA3, as assessed by isolating CA3 from CA1.	16	[4]

SD rat	10-30 d	13-60	CCh	CA1 and CA3	SP	400	2	2	5	Extracellular recording in SP	Higher concentrations of CCh (13-60 μM) produced short episodes of synchronous population discharges at a regular frequency (5-10 Hz). Separating CA1 from CA3, oscillations were found in isolated CA3, but not in isolated CA1, and dual field recordings in CA3 and CA1 revealed a positive latency (10-15 ms) in CA1.	3	[4]
SD rat	10-30 d	8-25	CCh	CA1 and CA3	SP	400	2	2	5	Extracellular recording in SP	Concentrations of CCh in the 8-25 μM range produced faster population discharges (40-50 Hz) in the gamma band in both CA1 and CA3. This oscillation was present in CA3 minislices, but was never observed in CA1 minislices.	6	[4]
SD rat	200-300 g	1-100	CCh	CA1	all	400	2	1	3	Extracellular recordings	The power spectra show a single distinguishable power peak in the gamma frequency band (45 Hz) in CA1 mini-slice. Maximal γ power was recorded at the SO-SP border.	78	[5]
SD rat	200-300 g	10	CCh	CA1 and CA3	SP	400	2	1	3	Extracellular recording in SP	In intact hippocampal slices CCh induced γ in both area CA3 and CA1.The γ in CA1a was phase-locked to the slow γ in CA3b. In these slices the power spectrum in CA1a had a single peak in the gamma frequency band with a dominant frequency of 33 Hz.	53	[5]

LE rat	3-8 m 350-500 g	-	Tail pinches (correlated with ACh release)	CA1	all	-	-	-	-	Extracellular recordings	ACh release was highly correlated with the appearance of both spontaneous and induced theta oscillations (such release lagged behind theta initiation by 25-60 s)	-	[6]
ChAT-Cre mouse	2-6 m	-	Optogenetic stimulation	CA1 and CA3	all	-	-	-	-	Extracellular recordings	Cholinergic stimulation completely blocked sharp wave ripples and strongly suppressed the power of both slow oscillations (0.5–2 Hz in anesthetized, 0.5–4 Hz in behaving animals) and supratheta (6–10 Hz in anesthetized, 10–25 Hz in behaving animals) bands. The same stimulation robustly increased both the power and coherence of theta oscillations (2–6 Hz) in urethane-anesthetized mice	-	[7]
CA1 mouse	15-23 d	5-10	CCh	CA1 and CA3	SP	450	2	2	2.5	Extracellular recordings	CCh-induced 31-Hz oscillations are generated within the CA3 area and propagate to CA1 in intact slice.	31	[8]

Table S21: **Network effects of ACh.**

[1] SD rat: Sprague Dawley rat, W rat: Wistar rat, LE rat: Long–Evans rat, G pig: Guinea pig.

[2] ACh: Acetylcholine, CCh: Carbachol, Musc: Muscarine

[3] All bath application except for [1] that used bath and focal application.

References

[1] Bianchi R, Wong RK. Carbachol-induced synchronized rhythmic bursts in CA3 neurons of guinea pig hippocampus in vitro;72(1):131–138. doi:10.1152/jn.1994.72.1.131.

[2] Williams JH, Kauer JA. Properties of carbachol-induced oscillatory activity in rat hippocampus;78(5):2631–2640. doi:10.1152/jn.1997.78.5.2631.

[3] Fisahn A, Pike FG, Buhl EH, Paulsen O. Cholinergic induction of network oscillations at 40 Hz in the hippocampus in vitro;394(6689):186–189. doi:10.1038/28179.

[4] Fellous JM, Sejnowski TJ. Cholinergic induction of oscillations in the hippocampal slice in the slow (0.5-2 Hz), theta (5-12 Hz), and gamma (35-70 Hz) bands;10(2):187–197. doi:10.1002/(SICI)1098-1063(2000)10:2<187::AID-HIPO8>3.0.CO;2-M.

[5] Pietersen ANJ, Ward PD, Hagger-Vaughan N, Wiggins J, Jefferys JGR, Vreugdenhil M. Transition between fast and slow gamma modes in rat hippocampus area CA1 in vitro is modulated by slow CA3 gamma oscillations;592(4):605–620. doi:10.1113/jphysiol.2013.263889.

[6] Zhang H, Lin SC, Nicolelis MAL. Spatiotemporal Coupling between Hippocampal Acetylcholine Release and Theta Oscillations In Vivo. The Journal of Neuroscience. 2010;30(40):13431–13440. doi:10.1523/jneurosci.1144-10.2010.

[7] Vandecasteele M, Varga V, Berényi A, Papp E, Barthó P, Venance L, et al. Optogenetic activation of septal cholinergic neurons suppresses sharp wave ripples and enhances theta oscillations in the hippocampus. Proceedings of the National Academy of Sciences. 2014;111(37):13535–13540. doi:10.1073/pnas.1411233111.

[8] Zemankovics R, Veres JM, Oren I, Hajos N. Feedforward Inhibition Underlies the Propagation of Cholinergically Induced Gamma Oscillations from Hippocampal CA3 to CA1;33(30):12337–12351. doi:10.1523/JNEUROSCI.3680-12.2013.
