# Supplementary material for: Community-based reconstruction and simulation of a full-scale model of the rat hippocampus CA1 region
Source: PLoS Biol. 2024 Nov 5;22(11):e3002861. doi: 10.1371/journal.pbio.3002861 (PMC11537418; doi:10.1371/journal.pbio.3002861)
Supplement: S22 Table — TTX used to inactivate fast sodium channels in all experiments except Hajos and Mody. D: Days, M: Months. (PDF) [file pbio.3002861.s052.pdf]

Pre Mtype	Post Mtype	Rat Age	[Ca2+]	Type	n	Frequency		Reference	Notes
						Mean	SEM		
Excitatory	Pyramidal	21-32 D	2,5	mEPSC	8	3.120	0.320	[1]	
Inhibitory	Pyramidal	20-28 D	2	sIPSC	3	5.567	2.541	[2]	KYNA blocks AMPA/NMDA
Inhibitory	Inhibitory	20-28 D		sIPSC	24	2.433	0.309	[2]	
Excitatory	Pyramidal		2	sEPSC	16	0.660	0.260	[3]	Soma
Excitatory	Pyramidal		2	mEPSC	5	0.130	0.040	[3]	Soma + TTX
Excitatory	Pyramidal		2	sEPSC	13	1.790	0.370	[3]	Dendrites
Excitatory	Pyramidal		2	mEPSC	11	0.300	0.030	[3]	Dendrites + TTX
Excitatory	Pyramidal	1.5-6 M	2	sEPSC	8	0.530	0.160	[4]	Soma in SP
Excitatory	Pyramidal	1.5-6 M	2	mEPSC	8	0.020	0.010	[4]	Soma in SP + TTX
Excitatory	Pyramidal	1.5-6 M	2	sEPSC	9	2.010	0.420	[4]	Dendrites in SR
Excitatory	Pyramidal	1.5-6 M	2	mEPSC	9	0.480	0.100	[4]	Dendrites in SR + TTX
Excitatory	Pyramidal	1.5-6 M	2	sEPSC	6	0.430	0.100	[4]	Dendrites in SO
Excitatory	Pyramidal	1.5-6 M	2	mEPSC	6	0.040	0.020	[4]	Dendrites in SO + TTX
Excitatory	Pyramidal	1.5-6 M	2	sEPSC	5	1.960	0.560	[4]	Soma in SO
Excitatory	Pyramidal	1.5-6 M	2	mEPSC	5	0.150	0.070	[4]	Soma in SO + TTX
Inhibitory	Pyramidal	1.5-6 M	2	sIPSC	8	16.820	2.210	[4]	Soma in SP
Inhibitory	Pyramidal	2.5-6 M	2	mIPSC	8	13.040	4.700	[4]	Soma in SP + TTX
Inhibitory	Pyramidal	3.5-6 M	2	sIPSC	9	17.290	2.300	[4]	Dendrites in SR
Inhibitory	Pyramidal	4.5-6 M	2	mIPSC	9	1.980	0.580	[4]	Dendrites in SR + TTX
Inhibitory	Pyramidal	5.5-6 M	2	sIPSC	6	21.150	3.990	[4]	Dendrites in SO
Inhibitory	Pyramidal	6.5-6 M	2	mIPSC	6	3.650	0.630	[4]	Dendrites in SO + TTX
Inhibitory	Pyramidal	7.5-6 M	2	sIPSC	5	32.090	7.780	[4]	Soma in SO
Inhibitory	Pyramidal	8.5-6 M	2	mIPSC	5	11.410	2.060	[4]	Soma in SO + TTX

Table S22: **Summary of estimated rates of spontaneous synaptic release in rat CA1.** TTX used to inactivate fast sodium channels in all experiments except [2]. D:Days, M:Months.

References

[1] Ito HT, Schuman EM. Distance-dependent homeostatic synaptic scaling mediated by a-type potassium channels. Front Cell Neurosci. 2009;3:15. doi:10.3389/neuro.03.015.2009.

[2] Hájos N, Mody I. Synaptic communication among hippocampal interneurons: properties of spontaneous IPSCs in morphologically identified cells. J Neurosci. 1997;17(21):8427–8442.

[3] Esclapez M, Hirsch JC, Ben-Ari Y, Bernard C. Newly formed excitatory pathways provide a substrate for hyperexcitability in experimental temporal lobe epilepsy. J Comp Neurol. 1999;408(4):449–460. doi:10.1002/(sici)1096-9861(19990614)408:4<449::aid-cne1>3.0.co;2-r.

[4] Cossart R, Hirsch JC, Cannon RC, Dinoncourt C, Wheal HV, Ben-Ari Y, et al. Distribution of spontaneous currents along the somato-dendritic axis of rat hippocampal CA1 pyramidal neurons. Neuroscience. 2000;99(4):593–603. doi:10.1016/s0306-4522(00)00231-1.
