# Supplementary material for: Community-based reconstruction and simulation of a full-scale model of the rat hippocampus CA1 region
Source: PLoS Biol. 2024 Nov 5;22(11):e3002861. doi: 10.1371/journal.pbio.3002861 (PMC11537418; doi:10.1371/journal.pbio.3002861)
Supplement: S23 Table — (PDF) [file pbio.3002861.s053.pdf]

Mtype	Mean angle (deg)	Angular deviation (deg)	Mean firing rate (Hz)	Min firing rate (Hz)	Max firing rate (Hz)	N.	Species[1]	Reference
SP_PVBC	271	68	7.3	3.4	17.6	5	rat	[1]
SP_AA	185	55	17.1	9.1	25.1	2	rat	[1]
SP_PC	20	65	-	-	-	6	rat	[1]
SO_OLM	19	57	4.9	2.5	6.1	3	rat	[1]
SP_BS	1	60	5.9	0.5	21.7	5	rat	[2]
SP_CCKBC	173	-	9.4	4.6	12.7	4	rat	[3]
SP_Ivy	30.7	63.1	4.2	-	-	4	rat	[4]

Table S23: **Phase tuning of rat CA1 morphological type.**

[1]SD rat: Sprague Dawley rat, W rat: Wistar rat, LE rat: Long–Evans rat, G pig: Guinea pig.

References

[1] Klausberger T, Magill PJ, Márton LF, Roberts JDB, Cobden PM, Buzsáki G, et al. Brain-state- and cell-type-specific firing of hippocampal interneurons in vivo;421(6925):844–848. doi:10.1038/nature01374.

[2] Klausberger T, Márton LF, Baude A, Roberts JDB, Magill PJ, Somogyi P. Spike timing of dendrite-targeting bistratified cells during hippocampal network oscillations in vivo;7(1):41–47. doi:10.1038/nn1159.

[3] Klausberger T. Complementary Roles of Cholecystokinin- and Parvalbumin-Expressing GABAergic Neurons in Hippocampal Network Oscillations;25(42):9782–9793. doi:10.1523/JNEUROSCI.3269-05.2005.

[4] Fuentealba P, Begum R, Capogna M, Jinno S, Márton LF, Csicsvari J, et al. Ivy Cells: A Population of Nitric-Oxide-Producing, Slow-Spiking GABAergic Neurons and Their Involvement in Hippocampal Network Activity;57(6):917–929. doi:10.1016/j.neuron.2008.01.034.
