# Supplementary material for: Community-based reconstruction and simulation of a full-scale model of the rat hippocampus CA1 region
Source: PLoS Biol. 2024 Nov 5;22(11):e3002861. doi: 10.1371/journal.pbio.3002861 (PMC11537418; doi:10.1371/journal.pbio.3002861)
Supplement: S24 Table — (PDF) [file pbio.3002861.s054.pdf]

Neuron Type	Mean rate (Hz)	SE rate (Hz)	N.	SD rate (Hz)	Recording condition	Source
Pyramidal cells	1.4	0.1	246	1.6	in vivo, behaving	[1]
SP interneurons	16.3	1.52	55	11.3	in vivo, behaving	[1]
Alveus/Oriens (a/o) interneurons	11.9	1.5	68	12.4	in vivo, behaving	[1]

Table S24: **Long-term discharge rates of rat CA1 neurons *in vivo* during theta periods.**

References

[1] Csicsvari J, Hirase H, Czurkó A, Mamiya A, Buzsáki G. Oscillatory Coupling of Hippocampal Pyramidal Cells and Interneurons in the Behaving Rat. J Neurosci. 1999;19(1):274–287. doi:10.1523/JNEUROSCI.19-01-00274.1999.
