# Supplementary material for: Community-based reconstruction and simulation of a full-scale model of the rat hippocampus CA1 region
Source: PLoS Biol. 2024 Nov 5;22(11):e3002861. doi: 10.1371/journal.pbio.3002861 (PMC11537418; doi:10.1371/journal.pbio.3002861)
Supplement: S25 Table — cav: calcium-voltage scan. osc control: oscillatory, ms: medial septum, SC: Schaffer collateral, N: Neuron, CN: CoreNeuron. (PDF) [file pbio.3002861.s055.pdf]

Campaign	Circuit Size	SC	Report On	Duration (s)	Time(s)	Max Mem per Node	N. of Nodes	Total Mem (GB)	Simulator
cav	cylinder	No	Soma+LFP	10.0	20927.09	636.35	6	3.729	N
cav	full	No	Soma+LFP	10.0	15891.12	3201.16	300	937.84	CN
cav	slice	No	Soma+LFP	10.0	16195.65	603.95	16	9.437	N
minis	cylinder	Yes	Soma+LFP	10.0	34241.17	2574.45	16	40.226	N
ms-input	cylinder	No	Soma+LFP	20.0	6220.22	700.16	60	41.025	N
sasaki	slice	Yes	Soma	1.5	5318.58	2083.16	20	40.687	N
sc-oscillatory	cylinder	Yes	Soma+LFP	10.0	15403.8	1236.87	36	43.484	N
sc-oscillatory	cylinder	Yes	Soma+LFP	10.0	3772.47	1202.79	36	42.286	CN
sc-oscillatory	full	Yes	Soma+LFP	10.0	35914.36	5315.56	300	1557.293	CN
sc-oscillatory	slice	Yes	Soma+LFP	10.0	7598.11	1928.43	36	67.796	CN
sc-unstructured	cylinder	Yes	Soma+LFP	10.0	17444.86	1448.93	36	50.939	N
zemankovich	slice	Yes	Soma+LFP	5.0	15888.6	2208.81	36	77.653	N

Table S25: **Resources and simulators used in validating the current CA1 model.** cav: calcium-voltage scan. osc control: oscillatory; ms: medial septum, SC: Schaffer collateral; N: Neuron; CN: CoreNeuron.
