# Supplementary material for: Community-based reconstruction and simulation of a full-scale model of the rat hippocampus CA1 region
Source: PLoS Biol. 2024 Nov 5;22(11):e3002861. doi: 10.1371/journal.pbio.3002861 (PMC11537418; doi:10.1371/journal.pbio.3002861)
Supplement: Attachment — Submitted filename: Reply to reviewers.pdf [file pbio.3002861.s056.pdf]

Response to Reviewers

Thank you for your careful and constructive review of our research article. We greatly value the considerable time and effort you have put into providing such comprehensive and perceptive feedback. Given the manuscript's length and complexity, we recognize that reviewing it thoroughly was no small task. Nonetheless, your insights have been crucial in refining the quality and clarity of our work.

We believe we have addressed all the points raised in a thorough manner. For issues raised more than once, we have repeated our responses to ensure each comment is fully addressed. Thanks to your expertise and critique, we hope we have adequately improved the manuscript to meet your satisfaction.

Black text = Reviewers' comments

Green text = Authors' responses

Reviewer #1

Reviewer #1: This tour de force work of Romani et al. develops a 3D model of the CA1 region of the hippocampus, based on experimental data by the authors and others. This model includes morphologically and biophysically detailed models of CA1 pyramidal cells as well as several types of interneurons and ensures diversity by a carefully curated cloning procedure of the available morphologies. In addition, connections (including synapse number, synapse strength and short-term plasticity properties) between different types of neurons are extensively validated. Schaffer collateral inputs and cholinergic modulation were also included in the model. The authors have written a very well documented and transparent materials and methods section and comment throughout the results section what results the model succeeds or fails to reproduce. Importantly, the authors have developed a web-based resource to share the model and experimental data (hippocampushub.eu) and organize an open course for its usage, to facilitate its uptake by the research community. All in all this is a very important and detailed methodological work. Nevertheless, and although I highly appreciate the difficulty of the task the authors undertook and its challenges, I have major concerns regarding the conceptual advance of the presented model, as well as it general applicability (with respect to its flexibility). Following are my major concerns, followed by minor comments.

Major:
1. In several points throughout the text, the authors claim that the main aim of this work is to be used as a tool to address a wide range of research questions (for example, abstract, line

30: "The unique flexibility of the model allows scientists to address a range of scientific questions.", discussion: Lines 507-509: "Importantly, the reference circuit model is, by definition, general: it was not created to reproduce a narrow spectrum of use cases but to be capable of addressing a wide range of research questions." and lines 591-594: "Third, while the traditional approach of constructing circuits for a specific use-case has short-term advantages for demonstrating proof-of-concept, in the long-term a community reference model must be valid across a range of use-cases.").
Yet, it is hard to conceptualise how the model can support the investigation of other CA1 functions, apart from oscillations and rhythmic activity that the authors are using in this study. For example, one of the well-studied CA1 functions is spatial navigation in general, and place field formation in particular, that depends both on EC input (Brun VH, et al., Neuron 2008) and burst firing of pyramidal neurons (Bittner et al., Nature Neuroscience 2015). How can this model be used to study this CA1 function?

We thank the reviewer for this comment. Our intention in the passages quoted was to mean that we did not tune the model to reproduce specifically one or few use-cases, so the model promises to be general and usable in a wider spectrum of cases. This is what makes it the first draft for a reference circuit for CA1. However, the current text could better convey the idea that in order to investigate CA1 functions other than the ones we have included, additional work may be required, depending on the phenomenon of interest to be reproduced at a given level of biological detail. Now, we clarify that the model can *potentially* address a range of scientific questions (line 34). In the current version of the model, the set of questions is limited by the extent of the model and its inputs (lines 523-530), while to investigate additional scientific questions the model needs to be expanded (lines 653-673). To illustrate the latter point, we used the example place cells.

2. The above is relevant also to the conceptual advance of this work. Although this model includes several additional features that were not present in previous models of the CA1 region (e.g. of Bezaire et al. 2016, as also described in the discussion section), no additional features are incorporated that would allow for the general usability of the model, going beyond the rhythmic activity (also studied in the previous modeling works). Such advances could be, for example, burst firing of pyramidal neurons; in this work all pyramidal neurons are classic accommodating, whereas it is known that CA1 pyramidal cells support burst firing (e.g., Jarsky et al., The Journal of Comparative Neurology 2008). This is an important issue, as burst firing is an important mechanism exploited for CA1 function (e.g., Bittner et al., Nature Neuroscience 2015). Another such example would be the incorporation of the known dorso-ventral differences along the CA1 region (Fanselow, Dong, Neuron 2011).

The reviewer brings up valid points. We appreciate that additional features would be desirable in a circuit model. However, in this first release of the model, we had to make some simplifications, mainly dictated by the data in our possession. In the specific examples mentioned, the recordings from LNMC and UCL did not contain bursting activity and all the cells were samples from the dorsal CA1. With electrophysiological recordings that show bursting activity, it would not be a problem to optimize cells with this feature. In our model, for example, we already have interneurons with bursting behavior (bAC). The lack of data availability also forced us not to include different subtypes along the dorso-ventral axis. To

incorporate this feature, one would collect different morphologies along the longitudinal axis, electrophysiological recordings, build corresponding models, and place the cells respecting the difference in density. Placing the cells with a specific density profile is feasible within our framework (see the non-uniform distribution along the radial axis of SR_SCA and SLM_PPA which is mentioned in paragraph 4.15.1).

New data are continuously released and our framework could consume these data to produce better models. In the Discussion, we have included an example of how the model can be updated to include new or better components such as single-cell models (lines 659-661, Appendix 1).

3. Finally, and also relevant with the general or specific applicability of the model of the previous comments, the model does not capture some features of CA1 oscillatory activity (also commented by the authors). Specifically, Goutagny et al. (2009) have experimentally shown that CA1 theta oscillations can arise independent of CA3 inputs in the isolated hippocampus in vitro. This is in contrast with the results of the authors (Section CA1 generation, lines 336-350).
Along the same lines, both for MS and CA3 input, phase locking or firing rate considerably deviates from the experimentally reported values for most types of interneurons (Figure 7B, D, Fig. 9B, D). This points that, despite the detailed nature of this model, essential components are missing to reproduce experimental results with respect to theta oscillations. Contrary, the author's model can reliably sustain gamma oscillations, initiated by the SC input (Fig. S27) and Figure 10 is very informative regarding the properties of the simulated CA1 circuit.

Thank you for raising this issue. While we cannot exclude the possibility that isolated CA1 does generate and maintain theta oscillations as reported by Goutagny et al (2009), research using the same preparation by the same lab raises some doubts about this interpretation of the results. Fig S6 in Jackson et al (2014, *Nature Neuroscience* 17 (10): 1362–70) shows that inactivating the subiculum (SUB) in an isolated whole mouse hippocampal preparation, as used by Goutagny et al (2009), removes theta (5 Hz) from CA1 and the remaining 2.5 Hz oscillations in CA1 match those of intact CA3. These new results suggest that isolated CA1 may not be able to generate robust theta oscillations but instead its oscillations are induced by SUB and CA3, which is what we found in the circuit model for CA3 input. So the theta oscillations in isolated CA1 observed in Goutagny et al (2009) might be explained if SUB tissue was not fully removed when cutting to isolate CA1 from CA3 (cf Fig S10 of Goutagny et al, 2009). We now explain this point in Discussion lines 545-555.

We agree with the reviewer that the results shown in Fig. 7 and 9 are different from experimental results, and we now make this point in lines 555-559. However, we have to put our model into perspective. Following a strict data-driven approach, we do not ‘adjust’ the model to reproduce a certain experiment. By refraining from tuning the model, we believe we made the model a useful reference for the community in that it:

1) is general and reproducible
2) allows more thoughtful evaluation and integration of the data and assumptions

Minor Comments:

Figure S3 B-C: Please use the same color for all the same cell types (e.g. this is not the case for the cells SP_Ivy and SP_OCKBC) to improve the information content of the figure.

Thank you for bringing this to our attention. We have revised the color scheme to ensure uniformity for each cell type.

Figure S4: Can the authors elaborate why there are many dendrites with the same birth/death distance (diagonal in plots)? Also what is the physiological meaning of the points above the diagonal?

Certainly and we appreciate the chance to elaborate. The appearance of points near the diagonal in the persistence diagram signifies the prevalence of dendrites with relatively short branch lengths within the morphology. This phenomenon is elucidated in Figure 7F (top) of Kanari et al. (2022, Cell Rep. 39(1):110586.), where color coding on the morphological tree visually distinguishes these features.

The utilization of radial (Euclidean) distance as the metric accounts for instances where points are situated above the diagonal, indicating dendritic branches that terminate closer to the soma compared to their bifurcation points.

Our decision to employ Euclidean distance as the primary metric for constructing these diagrams stems from its direct relevance to understanding morphological characteristics in a spatial context. This choice allows for a straightforward interpretation of dendritic branching patterns in relation to their physical proximity to the soma, an aspect crucial for our analysis of neuronal architecture. Euclidean distance offers a clear, intuitive measure of spatial arrangement, facilitating the identification of morphological features that are significant for our study objectives.

Figure S7: There is a discrepancy between the experimental and modeling data both for the bAP amplitude and the PSP attenuation for CA1 pyramidal neurons, can the authors please comment on the reasons for this?

Regarding the point raised by the reviewer, we did not have access to any dendritic recordings which would help us to better constrain the behavior of the cells at the level of dendrites. We only had somatic recordings and used the published data on bAP and PSP as validation data. In this figure, we checked whether the models could well predict the behavior at the level of the dendrites despite the fact that we did not directly constrain the dendrites. Qualitatively, both bAP and PSP decreases as a function of the distance, and bAP amplitude appears to be bimodal at distal dendrites. However, quantitatively, we see differences, as also seen in Hay et al., (2011. PLoS Comput Biol. Jul;7(7):e1002107.). We commented on this part in the manuscript (see lines 165, 167-169).

Figure S8, Figure Legend: Panel description seems mixed up (e.g. "F. Layer Assignment" should be "H. Layer Assignment".) Same applies in text references of this figure (e.g. line 851, correct ref. is maybe S8F?).

We appreciate you pointing this out. We fixed the references to the panels.

Line 368: "Figure 6A-C, left columns" maybe the authors mean "Figure 6C-E, left columns"\

Thank you for catching this. We fixed the reference to the panels.

Fig 7B. Leftmost panel: Please add in figure legend what the black arrows and shaded regions show. For all results in figure 7, are they generated under in vivo like conditions (1mM Ca+2) or in vitro like conditions (2mM Ca+)?

Thank you for drawing our attention to this. All simulations were generated under *in vitro*-like conditions (2mM Ca+). We added the correct explanation in the caption of Fig. 7.

611-612: "… solution, young adult rats (Sprague Dawley, 90-180 g) rats…" please delete the second "rats".

Thank you. We removed the repetition.

Section 4.7/ Table S4: What are the respective percentages for the 154 recordings of the authors (and how many recordings per morphological type)?

We agree this part was not well documented. We added the number of traces in the table S4 and a clarification in the Method.

Fig. S13C: What are the different green or blue dots? If they correspond to different m-type pairs, please indicate the respective pairs. This is of importance, as for some data points, the experimental and model connection probability is very different.

The reviewer raises an important issue. Experimental data points in S13C can be found in Table S8. We mentioned it in the legend of S13C so it will be clear for the reader. Where necessary, we amended all the figure captions so they point the readers to the corresponding tables (Figures 3, 7, 9, S10-17).

S14B, S15B. These panels are identical (repetitions). I would also guess that they should follow the connection probability, described in S13B (if there is no connection, the number of synapses should be zero), and this is not the case.

Thank you for addressing the appearance of the panels.in S14B and S15B, which are similar but not identical. The reviewer is right, however, in saying that the different connectivity matrices (Fig. S12B, S13B, S14B, S15B) look inconsistent. The problem was due to differences in sampling that may not include rare connections. We re-analyzed the connectome using the entire CA1. In the case of connection probability validation (figure S13C), we took the opportunity to specify that the sampling was done in a way such that the inter-somatic distance among pairs of neurons is not greater than 500 µm.

Fig. S15D-E: It would be useful to comment on the reasons for the discrepancy with the experimental data for SO_tri (panel D) and for SR_SCA (panel E).

We appreciate your suggestions. In our model we did not constrain the target preference of the different m-types. Interestingly, the predicted connectome showed that ~90% of the synapses made by the interneurons contact pyramidal cells, while the rest contact other interneurons. This is in close agreement with the experimental data and predictions made by Bezaire and Soltesz (2013). Ferraguti et al. (2005, J Neurosci. 25(45):10520-36.), however, analyzed 52 boutons belonging to SO_Tri and reported that 60% target interneurons and 40% pyramidal cells (see Table S11). If we believe that the experimental result is robust, the source of this discrepancy is unclear. For example, our SO_Tri morphological reconstruction may not be representative of the entire class, or there may be factors that favor the connections between SO_Tri and interneurons beyond the chance.

For SR_SCA, the explanation is much simpler. Experimental data come from Pawelzik et al 2002 (see Table S12). The morphology used in the experiment is visible in Fig 10A of Pawelzik et al 2002 and is well confined in SR. Instead, the morphology used in the model spreads also in SP and SO. See the slice image for the morphology 990611HP2 in https://www.hippocampushub.eu/model/experimental-data/neuronal-morphology/?instance=990611HP2&layer=SR&mtype=SR_SCA
We have now included these two points in the figure legend.

Line 1127, please correct typo: Mg^{2+} 2.4mM to 1.4mM.

Thank you for pointing this out. We changed Mg^{2+} 2.4mM to 1.4mM.

Line 1246: Referenced panel Figure 4D does not exist.

Much appreciated. We deleted “(shown in Figure 4D, orange trace)” which was a typo.

Reviewer #2

Reviewer #2 (Frances Skinner): In this 'Methods and Resources' submission, the authors present a full-scale atlas-based reference model of the rat CA1 hippocampus. This massive undertaking clearly required input and expertise from many people. It clearly presents improvements and extensions over existing methods, specifically in its inclusion of the curved shape of CA1 and cell placements within, while taking advantage of previously developed methods and algorithms (e.g., connectome determination and STP). Indeed, the curvature and electrode placement matter a lot for the specific electrical recordings obtained (as we have learned from the many LFPy related papers from Einevoll's group).

While it is clear that this is solid work, I think that there are various aspects that need to be addressed and/or clarified to make it clear to the community how this reference model could best serve as a resource moving forward.

1) "Community-driven" and "community-based" are used heavily, but it is not clear to me that this is a specific aspect of this work that should be emphasized. That is, many (most?) models use experimental data (as published) from many labs (i.e., community-based). Indeed, the recent publication of Hippocampome.org2.0 (elife) allows leveraging of published

experimental data to create Izhikevich type network models. Also, whether this reference model is 'community-driven' is not clear. Indeed, as the authors clearly would know, it has been discussed many times in various contexts (e.g., see Eriksson et al, eLife 2022 and Levenstein et al., J Neurosci 2023 for some viewpoints), that there are many different goal(s) that affect the type and extent of models developed in the community, and approaches that can be used. It seems to me that the community aspect is more about presenting a very detailed CA1 model (reference model) to the community to use as they state. As such,
I think it would more helpful and clear for the community if the work was presented in this context, as well as actually specifying questions it could address, rather than just saying that there is a 'wide range of research questions' (Discussion), or a 'range of scientific questions' (Abstract). As presented, it is about not having the need to adjust parameters (line 79) and having a unifying, multiscale model (line 52) etc.? If so, then the model goal is mainly (only?) about reproducing the biology, but not trying to understand it per se? For example, we clearly know that there is degeneracy in biological systems (consider the extensive work in STG system). In essence, it is in the authors' interest to be clear and specific about what they intend with their reference model, thus providing guidance for potential users and audience etc.

We agree with the reviewer that the community aspects of this work were not sufficiently well explained in the previous manuscript. In shortening the manuscript for submission we removed this explanation, which we have now restored (see lines 622-639). Briefly, "community" has two different aspects: creation and use. In the current study, a community of five labs (BBP, LNMC, UCL, KOKI, CNR), with different expertise and different roles, collaborated to create and share a consensus circuit model that could be used by the wider hippocampus community. This is what we mean by "community-based". Although we sought advice from the wider community during the construction phase through organized workshops, we appreciate from the reviewer's comment that it may be slightly misleading to claim it was a "community-driven" model, so we have replaced each mention of this term with "community-based". To acknowledge previous discussions on community-based modeling and standards in neuroscience, we now cite the Eriksson et al 2022 and Levenstein et al 2023 mentioned in the Introduction (line 101). We thank the reviewer for pointing out this omission and the suggested references.

While the initial goal of data-driven modeling is reproducibility, the ultimate goal is a model that can make accurate predictions that will help explain empirical phenomena. We now appreciate that we need to explain better how the model can be used and adapted by the community. In this regard, we added a paragraph to discuss how users can potentially change the model to incorporate new data or address scientific questions beyond the ones cited in the manuscript (lines 653-673). However, exploring the degeneracy example highlighted by the reviewer would have required systematically varying numerous neuronal and synaptic parameters — an endeavor unfortunately exceeding the current paper's scope and resources, and thus deferred to future investigations.

2) An advantage of their model is that they can largely reproduce experimental conditions (recording sites, extracellular concentrations etc.), and this may be the main usage of their reference model ('reproduce and reconcile')? However, when they state in the Abstract they 'reproduce and reconcile experimental findings', I am not quite sure which part of the paper they intend this to refer to (SC, theta, etc.). I felt confused in how the work was presented.

For example, in reconstructing the SC, they reproduce Sasaki et al - so is considered a validation? Or is this a 'reproduce and reconcile experimental findings' or ?? - later (line 573) they refer to a poor result that was rectified by less sampling(?) to match Sasaki et al. Is it that they heterogeneous cell population (in constructing the model) was too diverse to match experiments? Could the authors explain/expand on this? For Ach, the authors refer to the emergence of three activity regimes and note that
consistency is unclear. Is this something their model could perhaps reconcile? However, in general, the organization and flow of the paper was hard to grasp in their presentation of the overall development and using of the model.

We appreciate the comments made by both reviewers #1 and #2, and do not dispute the fact that our results are not strong enough to achieve a reconciliation between conflicting evidence such as the generation of theta oscillation. Our intention was to show how the reference circuit model could provide a means of testing alternative hypotheses, which we are able to do using theta oscillations as a use case. The model predicts that theta could be generated by some mechanisms, yet apparently not by others, and some of these mechanisms seem to be more compatible with *in vivo* data. At the same time, we acknowledge that more investigation and data are necessary to reconcile conflicting evidence regarding theta.

To improve the flow of the paper, we made some changes. First, we removed the word "reconcile" from the abstract. Second, we made more explicit the scope of our simulations on theta by saying that our goal is to reproduce a subset of experimental data and test alternative mechanisms for theta generation (lines 347-349). We believe that by removing the concept of "reconciling" and improving the introduction on theta, the rest of the paper should be clearer to the readers.

In the case of SC, our aim was to use Sasaki et al. to validate pathway parameters (see line 274, "We functionally validated SC projections reproducing Sasaki et al. (2006)"). In the experiments, 101 spiking cells in response to strong stimulation were randomly sampled to represent 100% response of the circuit and the I/O curve was determined post hoc from the response of these cells to progressively weaker stimulation. What we attempted to explain was that initially we had randomly sampled from all neurons in the model, whether they were activated or not, which gave us poorer results. When we exactly followed the experimental sampling we were able to reproduce the result. The point we were making was that this apparently minor difference in sampling had a big difference in the validation of the model. This was not related to any heterogeneity or diversity of the model. We amended the discussion to be clearer (lines 615-616).

Also in the case of ACh, our intention was to validate the model parameters (see line 306, "After modeling the effect of ACh on neuron excitability and synaptic transmission, we validated the effect of ACh at the network level against available data"). In this instance, the contradictory outcomes of the experiments precluded a quantitative validation. Our analysis suggests the existence of multiple regimes, as evidenced in the literature.

3) The theta section (2.21) was confusing in its intent - intended to be 'reproduce and reconcile'? They initially state that we do not know the trigger that generates theta oscillations in CA1 due to conflicting evidence, and go on to examine intrinsic generation and extrinsic via CA3 and MS (of course, they could all contribute in the biological system in different conditions). For intrinsic, the experimental work as cited is from Goutagny et al, but then they go on to compare with Bezaire et al (model work), and conclude that they could not generate theta in their reference model. Rather, it IS the case that theta occurs intrinsically in the biological system), so because their reference model could not show this, indicates that there is something lacking in the reference model it would seem? In the CA3 input part they say 'contrary to Goutagny…', and talk about circuit scales, and justify only using the cylinder circuit. There is a confusing flow/rationale/justification of what the authors intend in their presentation of the work. At the end of the CA3 part, it is about in vivo and in vitro differences. Perhaps they can present one of their model uses/questions to address as representing in vitro and in vivo differences. Along those lines, is it only extracellular Ca that is changed for these differences? The MS section seems to be about illustrating the theta rhythm via disinhibition can occur in their model? The section showing that delta to low gamma propagated best was interesting - were different Ach levels used? - a potential model usage for experimental interfacing? - are there a specific experiments one would do/point to? Again, in general, the organization/flow/rationale of the work presented is somewhat confusing. I think this all stems back to the first point above.

In taking on board these comments, we have rewritten the start of the theta section to make clearer the intent behind the presentation of the results, removing the reference to 'reconcile' (347-349). Here, our aim was to use the same model to examine the possible contribution of each candidate mechanism separately to see which are capable of generating theta and to compare our results with previous work such as Bezaire et al. We agree with the point that each may contribute to the biological system under different conditions and now mention this (see lines 555-556).

While we cannot exclude the possibility that isolated CA1 does generate and maintain theta oscillations as reported by Goutagny et al (2009), research using the same preparation by the same lab raises some doubts about this interpretation of these results. Fig S6 in Jackson et al (2014, *Nature Neuroscience* 17 (10): 1362–70) shows that inactivating the subiculum (SUB) in an isolated whole mouse hippocampal preparation, as used by Goutagny et al (2009), removes theta (5 Hz) from CA1 and the remaining 2.5 Hz oscillations in CA1 match those of intact CA3. These later results suggest that isolated CA1 may not be able to generate robust theta oscillations but instead its oscillations are induced by SUB and CA3, which is what we found in the circuit model for CA3 input. So the theta oscillations in isolated CA1 observed in Goutagny et al (2009) might be explained if SUB tissue was not fully removed when cutting to isolate CA1 from CA3 (cf Fig S10 of Goutagny et al, 2009). We now explain this point in Discussion lines 545-559.

We have deleted the 'contrary to Goutagny' phrase since this referred to their experimental observation that they found theta in whole hippocampal preparation but not in thick acute slice and we now see a comparison to our results as a non sequitur. The *in vitro* and *in vivo* differences relate only to the effect of different extracellular calcium levels and we have now

made this clearer in lines 370-371. Similarly, we have made it clearer MS results demonstrate theta rhythmic activity via disinhibition in line 431.

We are grateful to the reviewer for the suggestion regarding the propagation results. ACh was absent from these simulations. The doubt is probably derived by the fact that the replication of Zemankovics et al. (2013) required ACh, while the subsequent simulations did not. We amended the text to make it clear (lines 503-504). In addition, we are not aware of any single experiment we can point to that covers the range of stimulation without ACh *in vitro*. However, in some experiments with ACh or CCh, the results suggest that oscillations are generated in CA3 and transmitted in CA1 via SC (e.g. Zemankovics et al., 2013). In addition, Jackson et al (2014) shows that CA3 delta oscillations entrain CA1 delta when SUB is inactivated (mentioned earlier). We have rephrased the sentence in lines 513-514 to better convey the idea that the last simulations represent a prediction, which we can eventually test in ad hoc experiments.

We realized that Zemankovics et al. (2013) was missing from table S21 and we included it now.

4) The website and education MOOC is commendable and addressing the above points would be helpful for usage goals. The authors refer to a 'smaller version' (line 120) - it would be helpful to specify what they mean by this - the cylinder or slice circuit? Or something else? If something else, what could one investigate (relative to the full model)?

We thank the reviewer for these questions so that we can better address them in the manuscript. The MOOC uses a microcircuit which was built in 2020 and uses a simplified volume as described in Markram et al., 2015 (seven prisms with an hexagonal base tiled together). The reason for this difference is historical since the MOOC was built much earlier than our current atlas-based model. However, for education purposes, we also believe that it is a good compromise to have a comparable architecture but a simplified volume, which facilitates spatial analysis. We added a new section in Methods (see lines 1245-1253).

5) The authors refer to simulating a "scalable and reproducible circuit automatically". This sounds excellent but it would be helpful for potential users to provide some more details/examples for what is automated? Adding another cell type?

We appreciate that this is a recurrent point in the review and we added a new paragraph in the discussion session (lines 653-673) to answer these questions. We discuss how users can modify the model using the example of the addition of a new cell type as suggested by the reviewer (see Appendix 1).

6) To help for usage and moving forward, is the authors' intent to keep adjusting model parameters to reflect biology (e.g., dorsal/ventral aspects, deep/superficial pyramidal cell differences and so on that do not currently exist in the model). Maybe focusing on reproducing an experimental population spike or LFP recording output would be helpful to interface with experiment? Especially given the curvature that is modelled. Given that the

existing reference model can (line 318) "simulate slices of a certain thickness, change the extracellular concentration of ions, change temperature, and enable spontaneous synaptic events", maybe one should could design such experiments to go hand in hand with these variations? to help reconcile experimental differences maybe? Again, this all reflects back to the first point.

We thank the reviewer for prompting us to explain better the long-term aims and use of the reference model. The intent is to keep improving the biological accuracy of the model in composition, components, cell properties and connectivity including important differences within layers (e.g. deep-superficial axis) and within regions (e.g. dorsal-ventral axis). We envisage with sufficient data to improve this accuracy so that a closer coupling of experiment and model, as suggested, might then allow experiment results to more easily validate the model and for the model results to make predictions that are testable experimentally. An *in vitro* example could be to apply an extracellular electrical stimulus at different locations in model slices for a range of simulated conditions (e.g. temperature, extracellular ionic concentration, background synaptic activity) that are matched experimentally to understand dorsal and ventral response differences (e.g. Koutsoumpa & Papatheodoropoulos 2019 BMC Neurosci 20:35). An *in vivo* example might be to place a stereotaxically-aligned array of linear probes, oriented perpendicular to hippocampal layers, in the full circuit model to investigate traveling and standing waves phenomena in CA1 (e.g. Lubenov & Siapas 2009 Nature 459:534). In this way, a reference model would then be in a position to reconcile different experimental results. We discussed it in the last paragraph of the Discussion section (lines 653-673).

MINOR

- Bistratified cells seem to be a distinct type (e.g., see Harris et al. Plos Biology 2018 and Gouwens et al. Cell 2020), so not sure why two types of bistratified cells used?

We think the reviewer is referring to SP_BS and SO_BS. We used the convention of Markram et al. (2015) to distinguish morphological types based on the laminar distribution of axon, dendrites and soma. This convention has been followed also by other groups (e.g., see Attili et al. Eur J Neurosci. 2022).

- Table S3 - based on what refs? Estimated from where?

In addressing the reviewer's good question, values in S3 (and S4) came from the combination of different datasets. The details can be found in the subsection 4.14 (and 4.7) of the Methods and we have now made it clearer to the reader in the caption.

- Line 190 onward: "Predictions" of pathways stuff - what experiments are needed? Please clarify, as they seem to say they match data in other stuff later on in same paragraph.
Could you be specific about discrepancies?

Thank you for raising these questions. We realize that the second sentence of the paragraph may have led to confusion without adding salient information ("In the case of pathways for which we had no specific data, we were instead able to offer testable, model-based predictions"). We decided to delete it.

- line 957 - not sentences

We appreciate your careful reading and changed “the were ignored” to “they were ignored”, and “This resulted fewer neurons than expected” to “This resulted in fewer neurons than expected”.

- Fig 5 - caption typo ('back')

We changed “back” to “black”.

Reviewer #3 (Daniele Linaro): In this paper, the authors present a large-scale model of the CA1 region that incorporates a significant number of key features of this brain region. The effort is monumental and follows in the footsteps of Markram et al., 2015. I particularly appreciated the collaborative nature of this project, which will allow the community to operate as "beta-testers" of the model and its many components: indeed, as presented here, it is my opinion that it is very difficult for a reviewer to assess whether this model will be able to accurately predict experimental results outside those that have been described here. For this reason, I have no specific comments or recommendations on the manuscript, except to congratulate the authors for their important work.

Thank you for your thoughtful review and acknowledgment of our manuscript. We appreciate your recognition of the collaborative effort and the importance of our work in modeling the CA1 region.

We understand the challenge in assessing the predictive accuracy of our model beyond the presented experiments. It is indeed a complex computational endeavor, particularly in the context of modeling a subregion in the hippocampus. We share your view that community engagement will be essential in further validating and refining our model, providing new datasets, improvements to the algorithms, and expansions to add essential components to reproduce other experimental results of interest.

Our goal was to foster an open dialogue with the scientific community and provide a next generation model to address the challenges highlighted in your review. Thank you once again for your time and attention to our manuscript.
