# Supplementary material for: Community-based reconstruction and simulation of a full-scale model of the rat hippocampus CA1 region
Source: PLoS Biol. 2024 Nov 5;22(11):e3002861. doi: 10.1371/journal.pbio.3002861 (PMC11537418; doi:10.1371/journal.pbio.3002861)
Supplement: Attachment — Submitted filename: reply_to_reviewers_revision2.pdf [file pbio.3002861.s057.pdf]

Black text = Reviewers' comments
Green text = Authors' responses

Reviewer #1: I appreciate the additions/clarification made by the authors, and I look forward to seeing an efficient uptake of this model by the research community. I only have one minor comment, that does not affect my recommendation for acceptance.

Fig 7B. This previous minor comment was referring to the panel B (not A). It would be informative to add in the figure legend what the black arrows and shaded regions show (for the plots below "Simulation vs in vivo" subtitle in panel B).

We are sorry to have misinterpreted this comment. We made it clear in the legend that the arrows represent the mean phase angle (experimental data = black arrow; simulated = colored arrow) and grey shaded sector the experimental angular deviation.
